# Dynamic altruistic cooperation within breast tumors

**DOI:** 10.1186/s12943-023-01896-7

**Published:** 2023-12-14

**Authors:** Muhammad Sufyan Bin Masroni, Kee Wah Lee, Victor Kwan Min Lee, Siok Bian Ng, Chao Teng Law, Kok Siong Poon, Bernett Teck-Kwong Lee, Zhehao Liu, Yuen Peng Tan, Wee Ling Chng, Steven Tucker, Lynette Su-Mien Ngo, George Wai Cheong Yip, Min En Nga, Susan Swee Shan Hue, Thomas Choudary Putti, Boon Huat Bay, Qingsong Lin, Lihan Zhou, Mikael Hartman, Tze Ping Loh, Manikandan Lakshmanan, Sook Yee Lee, Vinay Tergaonkar, Huiwen Chua, Adeline Voon Hui Lee, Eric Yew Meng Yeo, Mo-Huang Li, Chan Fong Chang, Zizheng Kee, Karen Mei-Ling Tan, Soo Yong Tan, Evelyn Siew-Chuan Koay, Marco Archetti, Sai Mun Leong

**Affiliations:** 1 Department of Pathology, Yong Loo Lin School of Medicine, National University of Singapore, Level 3 NUH Main Building, 21 Lower Kent Ridge Road, Singapore, 119077 Singapore; 2 Department of Anatomy, Yong Loo Lin School of Medicine, National University of Singapore, MD10, 4 Medical Drive, Singapore, 117594 Singapore; 3 NUS Centre for Cancer Research (N2CR), MD6, Centre for Translational Medicine, National University of Singapore, 14 Medical Drive, #12-01, Singapore, 117599 Singapore; 4 Centre for Biomedical Informatics, Lee Kong Chian School of Medicine, Nanyang Technological University, Experimental Medicine Building, NTU Main Campus, 59 Nanyang Drive, Level 4, Singapore, 636921 Singapore; 5 Tucker Medical Pte Ltd, Novena Specialist Centre, 8 Sinaran Drive #04-03, Singapore, 307470 Singapore; 6 Raffles Cancer Centre, Raffles Hospital, 585 North Bridge Road, Singapore, 188770 Singapore; 7 Current address: Curie Oncology Pte Ltd, Mount Elizabeth Novena Specialist Centre, 38 Irrawaddy Road, Level 8, #08-29/30, Singapore, 329563 Singapore; 8 Department of Pathology, National University Hospital, Level 3 NUH Main Building, 21 Lower Kent Ridge Road, Singapore, 119077 Singapore; 9 Institute of Molecular and Cell Biology, Agency for Science, Technology and Research (A*STAR), Proteos, 61 Biopolis Drive, Singapore, 138673 Singapore; 10 Department of Biological Sciences, Faculty of Science, National University of Singapore, 14 Science Drive 4, Singapore, 117543 Singapore; 11 MiRXES Pte Ltd, JTC MedTech Hub, 2 Tukang Innovation Grove #08-01, Singapore, 618305 Singapore; 12 Department of Surgery, Yong Loo Lin School of Medicine, National University of Singapore, 1E Kent Ridge Road, NUHS Tower Block, Level 8, Singapore, 119228 Singapore; 13 Department of Laboratory Medicine, National University Hospital, Level 3 NUH Main Building, 5 Lower Kent Ridge Road, Singapore, 119074 Singapore; 14 CellSievo Pte Ltd, Block 289A, Bukit Batok Street 25, #15-218, Singapore, 650289 Singapore; 15 Department of Biochemistry, Yong Loo Lin School of Medicine, National University of Singapore, 8 Medical Drive, Singapore, 117594 Singapore; 16 Singapore Institute For Clinical Sciences, Brenner Centre for Molecular Medicine, 30 Medical Drive, Singapore, 117609 Singapore; 17 Department of Biology, Pennsylvania State University, W210 Millennium Science Complex, University Park, PA 16802 USA

## Abstract

**Background:**

Social behaviors such as altruism, where one self-sacrifices for collective benefits, critically influence an organism’s survival and responses to the environment. Such behaviors are widely exemplified in nature but have been underexplored in cancer cells which are conventionally seen as selfish competitive players. This multidisciplinary study explores altruism and its mechanism in breast cancer cells and its contribution to chemoresistance.

**Methods:**

MicroRNA profiling was performed on circulating tumor cells collected from the blood of treated breast cancer patients. Cancer cell lines ectopically expressing candidate miRNA were used in co-culture experiments and treated with docetaxel. Ecological parameters like relative survival and relative fitness were assessed using flow cytometry. Functional studies and characterization performed *in vitro* and *in vivo* include proliferation, iTRAQ-mass spectrometry, RNA sequencing, inhibition by small molecules and antibodies, siRNA knockdown, CRISPR/dCas9 inhibition and fluorescence imaging of promoter reporter-expressing cells. Mathematical modeling based on evolutionary game theory was performed to simulate spatial organization of cancer cells.

**Results:**

Opposing cancer processes underlie altruism: an oncogenic process involving secretion of IGFBP2 and CCL28 by the altruists to induce survival benefits in neighboring cells under taxane exposure, and a self-sacrificial tumor suppressive process impeding proliferation of altruists via cell cycle arrest. Both processes are regulated concurrently in the altruists by miR-125b, via differential NF-κB signaling specifically through IKKβ. Altruistic cells persist in the tumor despite their self-sacrifice, as they can regenerate epigenetically from non-altruists via a KLF2/PCAF-mediated mechanism. The altruists maintain a sparse spatial organization by inhibiting surrounding cells from adopting the altruistic fate via a lateral inhibition mechanism involving a GAB1-PI3K-AKT-miR-125b signaling circuit.

**Conclusions:**

Our data reveal molecular mechanisms underlying manifestation, persistence and spatial spread of cancer cell altruism. A minor population behave altruistically at a cost to itself producing a collective benefit for the tumor, suggesting tumors to be dynamic social systems governed by the same rules of cooperation in social organisms. Understanding cancer cell altruism may lead to more holistic models of tumor evolution and drug response, as well as therapeutic paradigms that account for social interactions. Cancer cells constitute tractable experimental models for fields beyond oncology, like evolutionary ecology and game theory.

**Supplementary Information:**

The online version contains supplementary material available at 10.1186/s12943-023-01896-7.

## Background

Cancer is conventionally regarded as a strictly cell-autonomous process in which gene mutations potentiate cell fitness and drive clonal competition and expansions in a selfish, competitive manner [1]. However, a growing body of evidence indicates that different tumor subpopulations may also cooperate as a society [2–4]. Early demonstrations of clonal cooperation between distinct tumor subpopulations in modulating drug sensitivity of cancer cells were first reported in the 1980-90s [5, 6]. A resurgence of interest in clonal cooperation in more recent times led to the uncovering of how interactions between distinct subpopulations can affect tumorigenesis, metastasis and therapeutic outcomes [7–12]. Some of these studies typically describe the mutual exchange of benefits between interdependent subpopulations, or mutualism, to account for observations of intra-tumor heterogeneity [3]. However, as such studies are primarily driven by the need to understand heterogeneity, and not the societal aspects of a tumor, there has been limited progress in identifying other possible types of social interactions beyond mutualism.

In contrast, very little is known in the study of cancer cells about the unilateral production of a collective benefit by a single tumor subpopulation at a fitness cost to self, which is a form of altruism that appears to go against the fitness-defined logic of clonal selection. Here, altruism is defined in an evolutionary sense – i.e. as “biological altruism”, with the cost and benefits measured in terms of reproductive or proliferative fitness [13]. Unlike mutualism which is mostly driven by self-interest [14], altruism entails self-sacrifice leading to societal benefits, which poses a challenge in explaining it within the framework of natural selection. Yet, it has provided the impetus that has driven the study of social behavior since Charles Darwin. Altruism has been widely studied in eukaryotes [15–20], prokaryotes [21–24] and viruses [25], and is thought to underlie the biological success of highly social organisms such as social bees, ants, termites and social wasps, which together account for 75% of the world’s total insect biomass [26]. Despite its ubiquitous observations, little is understood about altruism in the context of cancer cells [27, 28], where its occurrence can be even more puzzling. This is because the typical explanations for the evolution of altruism such as reciprocation [29] and kin selection [30] may not readily apply to rapidly mutating and genetically diverse cancer cells within tumors.

Here, we provide direct experimental demonstrations and mechanistic dissection of altruistic interaction in breast cancer cells and show how such interaction shape cancer processes, such as therapy refractoriness in our study. Combining multiple cell and molecular techniques with sociobiological theory and mathematical modeling based on evolutionary game theory, we uncover the mechanistic basis underlying the manifestation, persistence and social fate organization of altruistic subpopulation within the tumor. These findings indicate the existence of a dynamic social system that may underpin tumor malignancies and underscore the importance of deciphering the nature of social interactions amongst cancer cells for more accurate models of tumor evolution and drug response that account for both cooperative and competitive interactions.

## Methods

### Reagents & cell lines

#### Antibodies

CD45 (MCA87A647; Biorad), EpCAM (324206; BioLegend), digoxigenin (DIG) (11093274910; Roche), GFP (2555; Cell Signaling Technology), polyclonal IGFBP2 (3922; Cell Signaling Technology), monoclonal IGFBP2 (ab109284; Abcam), monoclonal IGFBP2 (MAB6741-SP; R&D Systems), monoclonal CCL28 (ab192600; Abcam), polyclonal CCL28 (ab196567; Abcam), monoclonal CCL28 (MAB717-SP; R&D Systems), monoclonal CCL28 (sc-376654, Santa Cruz Biotechnology), Bak1 (12105; Cell Signaling Technology), beta actin (sc-47778; Santa Cruz), H3ac (61637; Active Motif), GAPDH (sc-137179; Santa Cruz), histone H4ac (39243; Active Motif), PCAF (3378; Cell Signaling Technology), KLF2 (sc-28675X; Santa Cruz), Cas9 (61757; Active Motif), ChIP negative control IgG (53026; Active Motif), luciferase (NB600-307; Novus Biologicals), IGF-1R (AF-305-NA; R&D Systems), integrin α5 (AF1864; R&D Systems), integrin β1 (AF-1778-SP; R&D Systems), integrin αV (ab94704; Abcam), integrin β3 (AF-2266-SP; R&D Systems), integrin α2b (ab63983; abcam), CCR10 (MAB3478; R&D Systems), CCR3 (PAB13065; Abnova), CD24 (311118; BioLegend), CCNA2 (4656, Cell Signaling Technology). CDK2 (2546, Cell Signaling Technology), E2F3 (sc-56665, Santa Cruz), GAB1 (3232, Cell Signaling Technology), AKT(pan) (4691, Cell Signaling Technology), Phospho-AKT(Ser473) (4060, Cell Signaling Technology), Phospho-PI3 Kinase p85 (Tyr458)/p55 (Tyr199) (4228, Cell Signaling Technology), PI3 Kinase p85α (13666, Cell Signaling Technology), Phospho-Rb (Ser780) (8180, Cell Signaling Technology), Phospho-Rb (Ser795) (9301, Cell Signaling Technology), Phospho-Rb (Ser807/811) (8516, Cell Signaling Technology), IKKb (sc-8014, Santa Cruz), Rb (sc-102, Santa Cruz), RELA/NFκB p65 (sc-8008, Santa Cruz). anti-rabbit IgG antibody (7074P2, Cell Signaling Technology) and anti-mouse IgG antibody (sc-2025, Santa Cruz Biotechnology).

#### Cells

Bioware^®^ MDA-MB-231-luc2 and MCF-7-luc-F5 from Perkin Elmer; MCF-7, MDA-MB-231, MDA-MB-415, SK-BR-3, MDA-MB-468, MCF10A and HS578T were obtained from ATCC; HEK293FT from Thermo Fisher Scientific. These cell lines were authenticated using short tandem repeat profiling and checked for mycoplasma contamination using MycoAlert^TM^ Mycoplasma Detection Kit (Lonza).

#### Chemicals

4’,6-Diamidino-2-Phenylindole (DAPI) from Thermo Fisher Scientific; IDEAL miRNA assays from MiRXES; Superscript VILO enzyme from Thermo Fisher Scientific; RPMI and DMEM from Thermo Fisher Scientific; McCoy’s 5A modified medium, Leibovitz L-15 medium and EMEM from Lonza; Trichostatin A (T8552), 5-azacytidine (A2385), curcumin (C1386), anacardic acid (05506), MB-3 (M2449) from Sigma-Aldrich; nitro-blue tetrazolium and 5-bromo-4-chloro-3-indolyl-phosphate (NBT/BCIP) tablets from Roche; Lipofectamine 3000 from Thermo Fisher Scientific; mirVana miR-125b oligonucleotide mimic (4464066) and negative control oligonucleotide mimic (4464058) tagged with fluorescein from Thermo Fisher Scientific; siRNA against PCAF (sc-36198), HDAC3 (sc-35538), PCAF (sc-36198), p53 (sc-29435), Ah receptor (sc-29654), Sp1 (sc-29487), TIP60 (sc-37966), FOXO3 (sc-37887), KLF2 (sc-35818), IKKβ (sc-35644), β-catenin (CTNNB1; sc-29209), SMO (sc-40161), GSK-3β (sc-35527), NFκB p65 (sc-29410), NFκB p50 (sc-29408) and control (sc-37007) from Santa Cruz; azidohomoalanine (AS-63669) from AnaSpe; docetaxel from Sanofi-Aventis; VivoGlo Luciferin (P1043) from Promega; Recombinant IGFBP2 (350-06B-20) and Recombinant CCL28 (300-57-20) from Peprotech; PI3K inhibitor LY294002 (HY-10108) from MedChemExpress,; Akt Inhibitor IV (sc-203809) from Santa Cruz Biotechnology, ; control (339121) and miR-125b miRNA LNA inhibitor (339126) from Qiagen.

### Collection of blood from breast cancer patients

Peripheral blood drawn from breast cancer patients undergoing taxane-based chemotherapy at Tucker Medical Pte Ltd (Singapore) and Raffles Hospital (Singapore) between April 2012 to December 2017 were analyzed prospectively. The blood samples were collected in spray-coated K_2-_EDTA vacutainer tubes (Becton Dickinson) from patients with breast cancer. The blood samples were collected by staff at Tucker Medical Pte Ltd (Singapore) and Raffles Hospital (Singapore), after prior counseling and written informed consent. Both sampling and study protocols were approved by the Institutional Review Board (IRB) committee of the National University of Singapore (NUS).

### Circulating tumor cells (CTCs) isolation and profiling for microRNA (miRNA) expression

Blood collected from breast cancer patients was diluted 1:1 with phosphate-buffered saline (PBS) / bovine serum albumin (BSA, 5 g/L) / 2 mM EDTA and the diluted blood was filtered using a 40 μM cell strainer (Becton Dickinson) directly into the funnel filtration system (CellSievo) and filtered through the microsieve (developed by co-author M.H Li) at a flow rate of 500 μL of blood per min. Four 1-mL washes with PBS/5 g/L BSA/2 mM EDTA were performed, followed by a 30 min incubation with a 100 μL antibody mixture containing 20 μL 100 mg/mL DAPI (Thermo Fisher Scientific), 20 μL AlexaFluor-488 conjugated anti-CD45 antibody (MCA87A647, Biorad), 20 μL PE-conjugated anti-EpCAM (324206, BioLegend), and 60 μL PBS buffer. This was followed by four 1-mL washes with EDTA-free PBS/5 g/L BSA. CTCs were eluted in 500 μL PBS/5 g/L BSA from the microsieve by reversing the flow of the pump and collected in sterile 1.5 mL tubes. After centrifugation at 300*g* for 5 min at room temperature, the cell pellet was resuspended in 5 μL PBS/5 g/L BSA.

The eluate was spotted onto the wells of a MicroWell^TM^ Minitray (452256; Nunc) with 0.5 μL in each well as previously described [31]. Each well was examined under BX61 fluorescence microscope (Olympus). A small piece of FTA^®^ Elute paper (Whatman) was pressed against the bottom of the microwell with moderate force for 5 s to soak up and lyse the cells. The discs were transferred to fresh 1.5 mL Eppendorf tubes and washed in 500 μL 70% ethanol for five min before drying. The dried discs were soaked in 12-20 μL of diethyl pyrocarbonate (DEPC)-treated water. miRNAs were eluted at 95°C for 30 min. During the process, different forceps were used to handle the CTC-containing and CTC-null samples respectively to avoid cross-contamination.

Thirty miRNAs were analyzed for the CTC samples, though only 16 miRNAs showed markedly high expression in at least one patient-derived CTCs (as shown in Fig. S1). These were selected as previously described [31]. Analysis of miRNA expression was performed using IDEAL miRNA assays (MiRXES). Multiplex reverse transcription was carried out in 3.75 μL reaction volume containing 100 nM miRNA specific stem-loop RT primers, 1.3 mM dNTPs (Thermo Fisher Scientific), 1× RT buffer (Thermo Fisher Scientific), 1.07 U Rnase Inhibitor (Thermo Fisher Scientific), 1× Superscript VILO enzyme (Thermo Fisher Scientific), and 2 μL total miRNAs extracted using the FTA^®^ Elute card. The reaction was conducted at 42°C for 1 h using a thermal cycler (Eppendorf).

Real-time qPCR was performed on the 7900HT real-time PCR instrument (Thermo Fisher Scientific) using SYBR Green I. Thermocycling of cDNAs was performed with 10 min of initial denaturation at 95°C, followed by 40 cycles of 30 s denaturation at 95°C and 30 s annealing / extension at 60°C. 1.6 μL of each 1:10 diluted cDNA sample was subjected to real-time qPCR in a total volume of 8 μL in 1x IDEAL miRNA qPCR Master Mix with 1x miRNA specific qPCR assay (MiRXES). Raw threshold cycle (C_q_) values were calculated using the 7500 software v2.0.5 (Thermo Fisher Scientific) with automatic baseline and threshold settings. Each cDNA sample was run in duplicates for the qPCR stage. Mean C_q_ values were normalized to all miRNAs detected. The difference in C_q_ values was calculated for the two fractions (∆C_q_ = C_q_ of CTC-containing wells – C_q_ of CTC-null wells). Fold difference was obtained (2^∆Cq^ ) and this value indicates the extent of miRNA expression due to CTCs only. ∆C_q_ values for all miRNAs assayed for cancer patients were presented as bar chart (Fig. S1A-J).

### Monolayer cell culture

Immortalized cancer cells were cultured in 5% CO_2_ humidified incubator at 37°C using RPMI (for MCF-7 and MCF-7-luc-F5; Thermo Fisher Scientific), EMEM (for MDA-MB-231 and MDA-MB-231-luc2; Lonza), DMEM (for MDA-MB-415 and HS578T; Thermo Fisher Scientific), McCoy’s 5A modified medium (for SK-BR-3; Lonza) and MEM-F12 medium (for MDA-MB-468; Lonza). HEK293FT cells (Thermo Fisher Scientific) used for lentivirus production were cultured using DMEM. Culture medium was supplemented with 10% FBS (Thermo Fisher Scientific) and 1% Penicillin/Streptomycin (Thermo Fisher Scientific).

### Treatment with docetaxel and pharmacological inhibitors

Breast cancer cell lines were seeded to 70% confluency before a 12 h treatment with culture medium containing vehicle control (PBS) or docetaxel (Sanofi-Aventis). A 12 h duration of treatment was chosen as the terminal half-life of docetaxel in human body was reported to be ~12 h [32]. Medium was then replaced with fresh culture medium. Cells were left for 2-4 days before being assayed for various analyses.

For studies using pharmacological inhibitors, breast cancer cells were treated with trichostatin A (TSA; Sigma-Aldrich), 5-azacytidine (5-azaC; Sigma-Aldrich), curcumin (Sigma-Aldrich), anacardic acid (Sigma-Aldrich), MB-3 (Sigma-Aldrich) for 48-h, after which the cells were trypsinized and analyzed by FACS.

For study of effect of PI3K and AKT inhibitor, Mi/EGFP^High^ and Mi/EGFP^Low^ cells were sorted from MDA-MB-231^*miR-125bprom-EGFP*^ and MDA-MB-415 ^*miR-125bprom-EGFP*^ cell lines. 8,000 cells were seeded per well in 96-well plates. Cells were then treated with Ly294002 or AKT IV (MedChemExpress) for 16 h, after which the medium was replaced with fresh medium. 48 h after the addition of inhibitors, cell viability was assessed using CellTiter-Glo (Promega).

### miRNA in situ hybridization

Locked nucleic acid (LNA)^TM^–modified oligonucleotide probes labeled with DIG were obtained from Qiagen. The sequences of the probes are *hsa-miR-125b*: 5’-TCACAAGTTAGGGGTCTCAGGGA-3’ (18022-05), negative control: GTGTAACACGTCTATACGCCCA-3’ (90002) and U6: 5’-CACGAATTTGCGTGTCATCCTT-3’ (90002).

#### For Formalin-fixed paraffin-embedded (FFPE) primary tumor samples

FFPE patient samples from chemotherapy-treated and treatment-naïve patients were obtained from the Department of Pathology, NUS, with prior approval obtained from NUS IRB. 4 μM-thin sections of FFPE tissues adhered to glass slides were deparaffinized in three consecutive xylene baths for 5 min each, followed by 1 min each in serial dilutions of ethanol (99.9%, 96%, 70%) and one change of PBS. Slides were then digested with 30 μg/mL (for *hsa-miR-125b* and negative control probes) or 15 μg/mL (for U6 probe) of proteinase K (Qiagen) at 37°C for 10 min, washed twice with PBS, dehydrate in serial dilutions of ethanol (70%, 96%, 99.9%), and air-dried completely. Slides were then hybridized in incubation chambers for 2 h at 54°C in an oven, using 40 nM (for *hsa-miR-125b* and negative control) or 1 nM (for U6) probes diluted with miRNA ISH buffer (Qiagen). After hybridization, slides were rinsed once in 5x saline-sodium citrate (SSC) at 54°C, twice in 1xSSC at 54°C, twice in 0.2× SSC at 54°C, once in 0.2× SSC at room temperature and rinsed once in PBS. A blocking solution (0.1% Tween 20, 2% sheep serum, 1% BSA in PBS) was applied for 15 min at room temperature, before application of anti-DIG reagent (sheep anti-DIG-AP (Roche) at 1:100 in 0.05% Tween 20, 1% sheep serum, 1% BSA in PBS) overnight at 4°C. This was followed by three washes in PBS/0.1% Tween 20 (PBS-T), 3 min each. Finally, alkaline phosphatase substrate, freshly prepared by dissolving NBT/BCIP tablets (Roche), was added to the slides for 2 h at room temperature, followed by two changes of KTBT buffer (50 nM Tris-HCl, 150 nM NaCl, 10 nM KCl) for 5 min each change to stop the reaction. The slides were dehydrated in serial dilutions of ethanol (70%, 96%, 99.9%) and mounted in a resin-based medium. Sections subjected to miRNA *in situ* hybridization were counter-stained with Fast-red, so as to better visualize the staining of miR-125b. These sections were viewed using light microscope under phase contrast to ensure presence of sections after processing for miRNA *in situ* hybridization*.* Consecutive sections were subjected to haematoxylin and eosin (H&E) staining and analyzed by a board-certified pathologist to verify tumor content. Images of the slides were taken using the Nikon Eclipse TS100 microscope (Nikon) and image acquisitions were made using the NIS-Elements software equipped with DS-FI1C camera (Nikon). Certified pathologist /co-author Nga MN and scientist/co-author Koay ESC assessed and compared the miR-125b/negative/U6 probe-hybridized sections blindly.

#### For immortalized breast cancer cell line

MDA-MB-231^*miR-125bprom-EGFP*^ grown on cover slips were fixed with 4% paraformaldehyde (Sigma-Aldrich) in PBS for 10 min at room temperature, followed by 1 min each in serial dilutions of ethanol (70%, 96%, 99.9% - stepwise dehydration) and air-dried completely. Slides were then hybridized in incubation chambers for 1 h at 54°C in an oven, using 80 nM of *hsa-miR-125b* or negative control probes diluted with mRNA ISH buffer (Qiagen). After hybridization, slides were rinsed three times in 2x saline-sodium citrate (SSC) for 5 min each at 54°C and rinsed once in PBS at room temperature. A blocking solution (0.1% Tween 20, 2% sheep serum, 1% BSA in PBS) was applied for 15 min at room temperature, before application of anti-DIG reagent (sheep anti-DIG-AP; 11093274910; Roche) at 1:100 in 0.05% Tween 20, 1% sheep serum, 1% BSA in PBS) overnight at 4°C. This was followed by three washes in PBS/0.1% Tween 20 (PBS-T), 3 min each. Finally, alkaline phosphatase substrate, freshly prepared by dissolving NBT/BCIP tablets (Roche), was added to the slides for 2 h at room temperature, followed by two changes of KTBT buffer (50 nM Tris-HCl, 150 nM NaCl, 10 nM KCl) for 5 min each change to stop the reaction. The slides were dehydrated in serial dilutions of ethanol (70%, 96%, 99.9%) and immersed in PBS, before proceeding to the next step.

For subsequent immunohistochemical detection of EGFP, heat-induced epitope retrieval was performed with a 10 mM citrate buffer (pH 6) (Sigma-Aldrich) under pressure cooking. Sections were incubated overnight at 4°C with 1:50 dilution of anti-GFP antibody (Cell Signaling Technology) and developed using the EnVision G|2 Doublestain System kit (Dako) as per manufacturer’s protocol. Images of the slides were taken using the Nikon Eclipse TS100 microscope (Nikon) and image acquisitions were made using the NIS-Elements software equipped with DS-FI1C camera (Nikon). Fiji, a distribution of ImageJ with plugins for scientific image analysis, was used to determine the intensity of staining for both miR-125b *in situ* hybridization (purple) and EGFP immunochemical detection (brown).

### Cloning of promoter sequence of miR-125b gene

Based on sequence information downloaded from NCBI (GI number 528476600), we established a PCR reaction to clone the main promoter region (including three CpG islands) of hsa*-miR-125b-1* from human genomic DNA as extracted from MCF-7 cells. The promoter of miR-125b was cloned from human DNA using primers designed to flank a region of ~1500 bp. The primers contain *Cla*I and *Bam*HI restriction sites to facilitate cloning into the lentiviral eGFP-reporter vector, pLU-Jarid1Bprom-eGFP-pBlast, a kind gift from Meenhard Herlyn and Mizuho Kalabis of Wistar Institute, USA (The JARID1B promoter was excised away before cloning in miR-125b-1 promoter). We chose MDA-MB-231, HS578T and MDA-MB-415 cells for stable lentiviral transduction because better read-outs were expected from cell lines with relatively high endogenous miR-125b expression (Fig. S2F). Cell lines with the lentiviral construct integrated into the genome are denoted as MDA-MB-231^*miR-125bprom-EGFP*^, HS578T^*miR-125bprom-EGFP*^ and MDA-MB-415^*miR-125bprom-EGFP*^, ^*EGFP*^ and clones from single cells were established. Fluorescent images of these cells were taken using BX61 fluorescence microscope (Olympus).

Primer pair used:

- miR-125b-1 promoter forward 5’ – AGT**ATCGAT**GATGCCTTAGTGCATCCAGC – 3’ (containing *Cla*I site in bold)
- miR-125b-1 promoter reverse 5’ – ATA**GGATCC**AACAGGTGGTATGGTATTTCTTC - 3’ (containing *Bam*HI site in bold)

While miR-125b (mature miRNA) is transcribed from two different genes: *hsa*-*miR*-*125b*-*1* (chromosome 11) and *hsa*-*miR*-*125b*-*2* (chromosome 21), the transcriptional activity of *miR*-*125b*-*2* has previously been reported to be low in both MCF-7 and MDA-MB-231 cell lines [33]. In line with this evidence, the levels of miR-125b transcripts (as visualized using miRNA *in situ* hybridization) and Mi/EGFP (as visualized using immunohistochemistry using antibody targeting EGFP [Cell Signaling Technology]) were shown to be correlated (Pearson correlation coefficient value *r* = 0.8342 without no treatment, *r* = 0.9051 with docetaxel treatment; Fig. S2D), supporting the notion that the promoter of *hsa-miR-125b-1* gene is very likely responsible for most of the mature miR-125b transcripts in MDA-MB-231^*miR-125bprom-EGFP*^.

### Production of lentivirus and transduction

HEK293FT cells (Thermo Fisher Scientific) were seeded on 10-cm plates till 80% confluency before transfection was performed with 3 μg of the pLU-miR125bprom-eGFP-pBlast vector, 9 μg of ViraPower packaging mix (Thermo Fisher Scientific) and 36 μL of Lipofectamine 3000 (Thermo Fisher Scientific). The plates were incubated overnight at 37°C in 5% CO_2_ before the culture medium was replaced with fresh medium. The eventual virus-containing medium was harvested 48 h post-transfection and subjected to centrifugation at 3,000 rpm for 5 min to remove cell debris. The medium was then passed through a 0.45 μM filter (Sartorius) and added to breast cancer cell lines (MDA-MB-231, HS578T, MDA-MB-415) supplemented with 6 μg/mL Polybrene (Sigma-Aldrich). Four days later, the Mi/EGFP-positive transduced cells were isolated by fluorescence-activated cell sorting (FACS) on the MoFlo XDP cell sorter (for HS578T and MDA-MB-415; Beckman Coulter), or in BD FACSVantage cell sorter (for MDA-MB-231; BD Biosciences). Single cell clones were isolated by limiting dilution and then expanded for further experiments.

### Spheroid culture and experiments

Aliquots of 5,000 MDA-MB-231^*miR-125bprom-EGFP*^ cells were seeded in round-bottomed, low-adhesion 96-well plates (Corning) and subjected to centrifugation at 1,000 *g* for 10 min. The cells were cultured in 5% CO2 humidified incubator at 37°C using EMEM (Lonza). Culture medium was also supplemented with 10% FBS (Thermo Fisher Scientific) and 1% Penicillin/Streptomycin (Thermo Fisher Scientific). Fully-formed spheroids were ready for experimental assays 3-5 days later. The spheroids were treated with fresh culture medium containing vehicle (PBS) or docetaxel (Sanofi-Aventis) for 2 days before being harvested for subsequent processing and immunohistochemical staining.

The harvested spheroids were washed three times with PBS and fixed in 4% paraformaldehyde (Sigma-Aldrich) overnight. Fixed tissues were subjected to tissue processing and then embedded in paraffin wax. The resulting FFPE specimens were sectioned into 7 μM slices. Sections were used for immunohistochemistry to look at Mi/EGFP expression across the cross-section of the spheroids. Heat-induced epitope retrieval was performed with 10 mM citrate buffer (pH 6) (Sigma-Aldrich) using pressure cooking. Sections were incubated overnight at 4°C with 1:50 anti-GFP antibody (Cell Signaling Technology) and developed using the EnVision G|2 Doublestain System kit (Dako) as per manufacturer’s protocol.

### Transfection of miRNA oligonucleotide mimic

Given that taxane-induced high expression of miR-125b appears to be transient as observed in the CTCs (Fig. S1A-J) and breast cancer cell lines MDA-MB-231^*miR-125bprom-EGFP*^ (Fig. S2E), we chose to transfect synthetic miRNA mimics into the breast cancer cell lines (MCF7, SK-BR-3, MDA-MB-468, which harbor relatively lower levels of miR-125b, when compared to MDA-MB-231, HS578T and MDA-MB 415, as previously reported [34] and as shown in Fig. S2F), instead of making cell lines with permanent overexpression. 90 nM of miR-125b oligonucleotide mimic and negative control oligonucleotide mimic tagged with fluorescein (Thermo Fisher Scientific) were transfected into MCF-7 cells using Lipofectamine 3000 (Thermo Fisher Scientific) following the manufacturer’s instructions. Transfection of miR-125b mimic into MDA-MB-231 breast cancer cells reduced the expression of Bak1, a known target of miR-125b [35] (Fig. S5A,M) – this demonstrates that the mimics were functionally active.

### Transfection of siRNA

Cells were plated in six-well plates and were grown to 50- 60% confluency, before transfection. For siRNA inhibition studies, the cells were transfected with siRNA against PCAF (Santa Cruz), p53 (Santa Cruz), Ah receptor (Santa Cruz), Sp1 (Santa Cruz), TIP60 (Santa Cruz), FOXO3 (Santa Cruz), KLF2 (Santa Cruz), IKKβ (Santa Cruz), β-catenin (Santa Cruz), SMO (Santa Cruz), GSK-3β (Santa Cruz), NFκB p65 (Santa Cruz), NFκB p50 (Santa Cruz) and control (Santa Cruz) at a final concentration of 100 nM using Lipofectamine 3000 (Thermo Fisher Scientific) following the manufacturer’s instructions.

### Cell viability assay

Cell viability was assessed using the CellTiter 96 Aqueous One Solution Proliferation Assay (MTS; Promega). Briefly, MTS solution was added to each well of the 96-well plates, the plates were incubated at 37°C in a CO_2_ cell incubator for 2 h, and the absorbance rates were measured at 492 nm using a microplate reader (Tecan).

### Cell culture experiments to derive “Relative Survival” and “Relative Fitness”

**Fig. 1:**
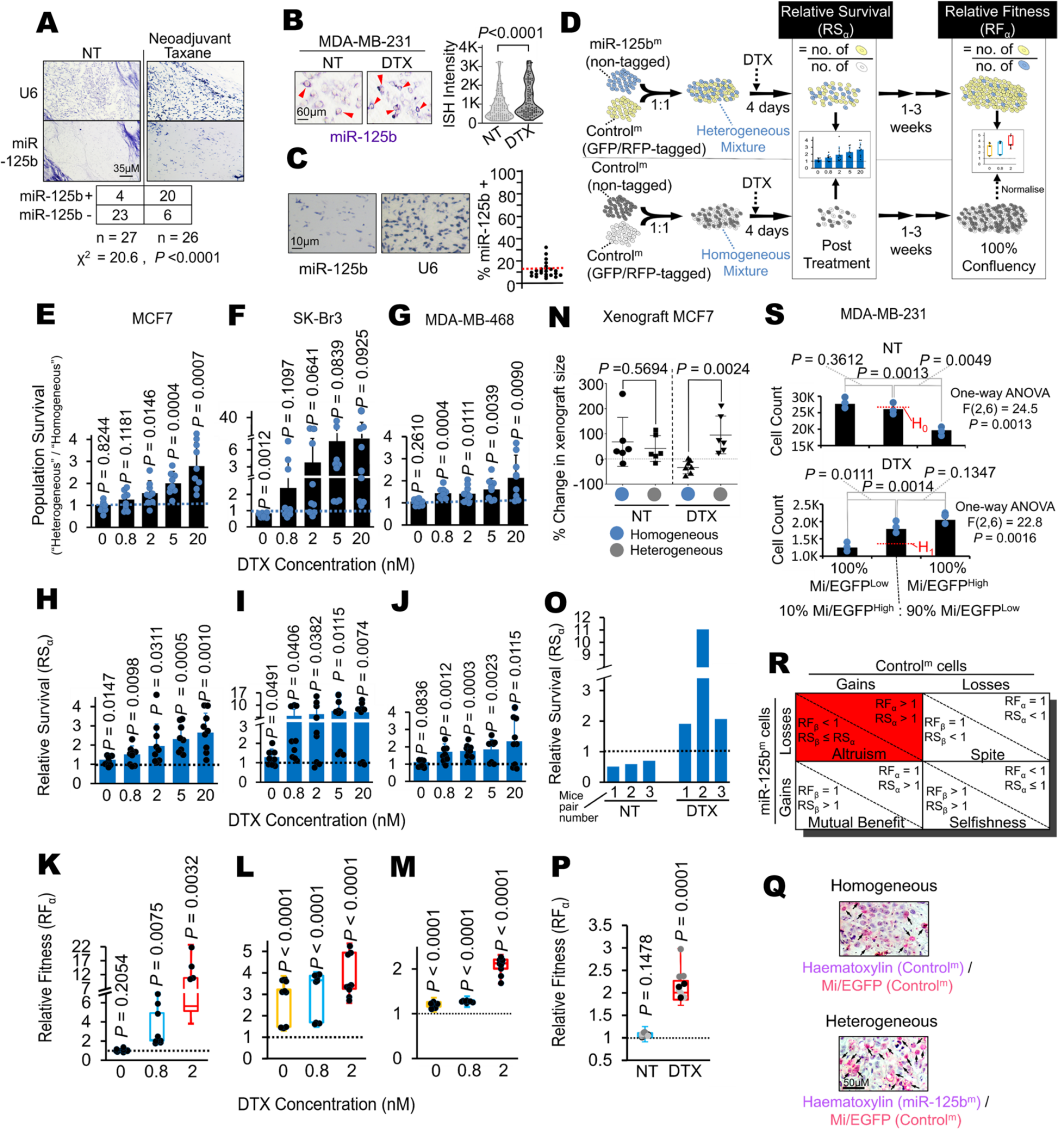
Breast cancer cells exhibit altruistic cooperation. **A ***In situ* hybridization for miR-125b or U6 in FFPE breast cancer tissues from patients with (*n*=26) or without (*n*=27) neoadjuvant taxane treatment. **B** Staining for miR-125b in MDA-MB-231^*miR-125bprom-EGFP*^ cells. Left: stained cells with or without docetaxel exposure. Red triangles: cells with high miR-125b. Right: quantification of miR-125b expression. **C** Left: Higher magnification of (A). Right: Quantification of percentage of cells with miR-125b vs. U6 expression for 24 miR-125b^+^ patient samples from (A). **D** Schema showing how “Relative Survival (RS_α_)” and “Relative Fitness (RF_α_)” were measured (See “Methods” and Supplementary Note 1). **E-G** Bar charts depicting ratio of heterogeneous vs. homogeneous total cell count in early post-treatment for indicated breast cancer cell lines. **H-J** Bar charts depicting RS_α_ ratio for indicated breast cancer cell lines. **K-M** Box plots of RF_α_ for “heterogeneous” group of indicated breast cancer cell lines (except cells exposed to 5 and 20nM docetaxel, which did not survive past 3 weeks). **N** Percentage change in tumor size for MCF-7 xenograft of “homogeneous” or “heterogeneous” study, with or without docetaxel treatment. **O** Bar chart depicting RS_α_ ratio, xenograft mice from (N), with or without docetaxel exposure. **P** Box plot of RF_α_ for “heterogeneous” xenograft tumor, with or without docetaxel treatment. Dot: single section read; dots of same colour: sections imaged from same animal. **Q** Representative images of xenograft tumor biopsy from “homogeneous” and “heterogeneous” group. Black arrows: cells stained positive Mi/EGFP. **R** Matrix for classifying social behaviour based on RS and RF (See Supplementary Note 1). **S** Viability of MDA-MB-231^miR-125bprom-EGFP^ cells of cultures of Mi/EGFP^High^ and Mi/EGFP^Low^ pure and mixed culture. Red dashed lines show the expected cell viabilities of mixed culture under hypothesis H_0_ (Upper, without docetaxel) or H_1_ (Lower, with 5 nM docetaxel). Without docetaxel, cell viability for mixed culture is not significantly different from H_0_ (two-tailed, T= -0.7241, degree of freedom = 2, *P* = 0.5442). With docetaxel, cell viability was significantly greater than H1 (two-tailed, T= 4.9884, degree of freedom = 2, *P* = 0.0379), indicating cooperative effect of minority Mi/EGFP^High^ cells in promoting population-wide survival. Experiment repeated three times, representative set shown (B). Data are mean ± s.d. from three independent biological sets of three technical replicates (E-M). *n* = six independent animals (N), from which three were analyzed for (O), and remaining three for (P,Q). Statistical analysis was performed using Chi-square test (A), two-tailed Mann Whitney *U* test (B), two-tailed one sample *t*-test against 1.0 (E-M, P), two-tailed unpaired *t*-test (N) and one-way ANOVA with post-hoc Tukey HSD (S). NT: no treatment; DTX: docetaxel treatment. Exact *P* values are shown

Immortalized breast cancer cell lines of different molecular subtypes such as MCF-7 (estrogen receptor-positive, progesterone receptor-positive, HER2 receptor-negative), SK-BR-3 (estrogen receptor-negative, progesterone receptor-negative, HER2 receptor-positive) and MDA-MB-468 (estrogen receptor-negative, progesterone receptor-negative, HER2 receptor-negative) tagged with GFP/RFP and non-tagged parental cells were transfected with either negative control or miR-125b mimics tagged with fluorescein (Thermo Fisher Scientific), and FACS-sorted 24 h later on the MoFlo XDP cell sorter (Beckman Coulter) to select for positively transfected cells for downstream experiment. FACS-selected cells were mixed in 1:1 ratio in the following ways: a GFP/RFP-tagged control mimic-transfected cells / non-tagged control mimic-transfected cells mix (the “homogeneous group”), or a GFP/RFP-tagged control mimic-transfected cells / non-tagged miR-125b mimic-transfected mix (the “heterogeneous group”) (Fig. 1D, Supplementary Note 1). For the “heterogeneous group”, the cells were also reciprocally tagged (i.e. non-tagged control mimic-transfected cells mixed with GFP/RFP-tagged miR-125b mimic-transfected mix) to negate away the effect that overexpression of fluorescent proteins might have on the cells’ behavior. A total of 10,000 cells were seeded into each well of a 96-well plate. Cells were then treated with vehicle (PBS) or docetaxel (0.8 – 20 nM; Sanofi-Aventis) for 12 h.

To derive values for the parameter “Relative Survival” (RS), the remaining number of cells (GFP/RFP-tagged vs. non-GFP/RFP-tagged) in each well was determined 4 days after docetaxel treatment using the Attune NxT Flow Cytometer (Thermo Fisher Scientific). The ratio of GFP/RFP-tagged vs. non-tagged cells in “homogeneous” mixture was normalized to 1:1, and the same normalization factor was applied to the corresponding “heterogeneous” mixture for each treatment group. Data acquisition and analysis was performed on the Attune NxT software v 2.5 (Thermo Fisher Scientific). Refer to “Supplementary Note 1” on how RS_α_ and RS_β_ were calculated.

To derive values for the parameter “Relative Fitness” (RF), the cells were allowed to expand over a span of 1-3 weeks, till 100% confluency was achieved in a larger well of a 24-well plate format. The relative number of GFP/RFP-tagged versus non-GFP/RFP-tagged cells in each well was analyzed on a BD LSRFortessa X-20 Cytometer (BD Biosciences). Data acquisition was performed using the FACSDiva 7.0 software (BD Biosciences) and the collected data were analyzed on the FlowJo 9 software (BD Biosciences). The ratio of GFP/RFP-tagged vs. non-tagged cells in “homogeneous” mixture was normalized to 1:1 following clonal expansion in regrown population, and the same normalization factor was applied to the ratio of GFP/RFP-tagged vs. non-tagged cells in the “heterogeneous” mixture. A normalized RF_α_ of 1.0 indicates that there was equal number of Control^m^- and miR-125b^m^-derived daughter cells in the regrown population, while a value of more than one indicates that there were more Control^m^-derived daughter cells than that of miR-125b^m^. A one-sample *t*-test was used to determine if RF_α_ values obtained in our study are significantly different from a reference value of 1.0.

For “fitness rescue” experiments, both “homogeneous” and “heterogeneous” mixture of cells (MCF7 or MDA-MB-468) were established as described above, with prior transfection of the Control^m^ cells with control siRNA (Santa Cruz) and miR-125b^m^ cells with control siRNA or siRNA against control, *CTNNB1, SMO, GSK3β, IKBKB, p50 or p65* (Santa Cruz). After treatment with 0.8 nM docetaxel (Sanofi-Aventis), RF_α_ values were determined as described above. A sublethal drug dose of 0.8 nM was chosen to ensure survival of sufficient cells especially after knocking-down of components of signaling pathway such as NF-κB which is known to support cancer cell proliferation. The percentage rescue in fitness disadvantage was calculated as follows:

$$\%\;\mathrm R\mathrm e\mathrm s\mathrm c\mathrm u\mathrm e\;\mathrm i\mathrm n\;\mathrm F\mathrm i\mathrm t\mathrm n\mathrm e\mathrm s\mathrm s\;\mathrm D\mathrm i\mathrm s\mathrm a\mathrm d\mathrm v\mathrm a\mathrm n\mathrm t\mathrm a\mathrm g\mathrm e=\left(\frac{{RF}_\alpha of\;''heterogeneous\;group''with\;control\;knockdown-{RF}_\alpha of\;''heterogeneous\;group''\;with\;gene\;knockdown}{{RF}_\alpha of\;''heterogeneous\;group''with\;control\;knockdown-{RF}_\alpha of\;''homogeneous\;group''\;with\;control\;knockdown}\right)\ast100\%$$

### Cell culture experiment to determine association between percentage benefit attained and percentage of altruists

MCF-7 breast cancer cells were grown to 70% confluency in 96-well format cell culture plate, and then transfected with either control or miR-125b mimics tagged with fluorescein (Thermo Fisher Scientific), and FACS-sorted 24 h later on the MoFlo XDP cell sorter (Beckman Coulter) to select for positively transfected cells. MiR-125b mimics-transfected (miR-125b^m^) cells were added to control-mimics transfected cells (Control^m^) at ratio of 0:100 (0% of total population after mixing), 1:99 (1%), 1:9 (10%), 3:7 (30%) and 1:1 (50%). The mixture was allowed to settle for 48 h, before they were subjected to treatment with 2 nM docetaxel (Sanofi-Aventis) for 12 h. Four days after treatment, the cells were assessed for cell viability using MTS assay kit (CellTiter 96 Aqueous One Solution Proliferation Assay; Promega), as described above. Percentage benefit attained for each altruist (miR-125b^m^ cells) percentage (denoted as x %) studied was derived using the following formula:

$$Percentage\;Benefit\;for\;x\%\;of\;altruist=\frac{MTS\;reading\;for\;x\%-MTS\;reading\;for\;0\%}{MTS\;reading\;for\;50\%-MTS\;reading\;for\;0\%}$$

We performed linear and polynomial (quadratic) regression analyses to determine the best-fit shape of the percentage benefit–percentage altruist relationship. These analyses were performed using R package *drc*.

To measure the change in the percentage benefit per percentage altruist increase, as we move from one altruist percentage (x %) point studied to another (y %) (marginal benefit), we used the following formula:

$$Marginal\;benefit=\frac{Percentage\;benefit\;for\;y\%of\;altruists-Percentage\;benefit\;for\;x\%\;of\;altruist}{\left(y\%-x\%\right)+1\%}$$

The category value used would be (x + y)/2. Plotting the marginal benefit against its category value gave us the marginal benefit plot that showed us the rate of change in percentage benefit with increasing altruist percentage.

### Drug screen

Inhibitors and agonists were obtained from MedChem Express (USA). 3,000 cells from miR-125b^m^ or Control^m^ MDA-MB-231 cell line were seeded in 96-well plates in triplicate wells 24 h before the addition of drugs. Two different drug concentrations were used for individual inhibitor/agonist from the NF-κB library (100 and 1,000 nM) and Wnt/Hedgehog/Notch library (10 and 100 nM). Cell viability was assayed 6 days after drug treatment with MTS assay. The percentage change in cell viability was calculated as follows:

$$\%\;change\;in\;cell\;viability=\left[\left(\frac{MTS\;read\;for\;miR-125b^mcells\;with\;drug-MTS\;read\;for\;miR-125b^mcells\;without\;drug}{MTS\;read\;for\;miR-125b^mcells\;without\;drug}\right)-\left(\frac{MTS\;read\;for\;{Control}^mcells\;with\;drug-MTS\;read\;for\;{Control}^m\;cells\;without\;drug}{MTS\;read\;for\;{Control}^m\;cells\;without\;drug}\right)\right]\ast100\%$$

### Luciferase reporter assays

For study of miR-125b binding to mRNA, HEK293FT cells were seeded at 30,000 cells per well on a 96-well plate. The luciferase reporter plasmids with the 3'-UTR sequences for E2F3 (Active Motif), CDK2, GAB1 and CCNA2 (Genecopeia), or their mutagenized versions, were co-transfected with negative control microRNA mimic or miR-125b mimic using the Lipofectamine 3000 reagent (ThermoFisher Scientific). After 48 h of incubation, cells were assayed using the Dual-Luciferase Assay (Promega) or the LightSwitch Luciferase Assay (for E2F3, Active Motif). Mutagenesis of miR-125b binding sites on the 3'-UTR sequences was performed with the Q5 Site-Directed Mutagenesis Kit (New England Biolabs).

For study of NF-κB activity, miR-125b^m^ or Control^m^ cells (MCF7 or MDA-MB-468) were established as described above, with prior transfection of the Control^m^ cells with control siRNA (Santa Cruz) and miR-125bm cells with siRNA against control, IKBKB, p50 or p65 (Santa Cruz). 3,000 cells were seeded in a 96-well plate 24 h before performing transfection for NF-kB or reporter plasmids. The NF-kB reporter assay was conducted using the Cignal NF-κB Reporter Assay Kit (Cat CCS-013L; Qiagen) following the manufacturer’s instructions. Normalization was performed for all experiments. Percentage change in NF-kB activity was calculated as follows:

$$\%\;\mathrm R\mathrm e\mathrm d\mathrm u\mathrm c\mathrm t\mathrm i\mathrm o\mathrm n\;\mathrm i\mathrm n\;\mathrm m\mathrm i\mathrm R-125\mathrm b-\mathrm{induced}\;\mathrm{NF}-\mathrm{kB}\;\mathrm{Reporter}\;\mathrm{Activity}=\left(\frac{Reading\;for\;miR-125b^m\;cells\;with\;control\;knockdown-Reading\;for\;miR-125b^mcells\;with\;gene\;knockdown}{Reading\;for\;miR-125b^m\;cells\;with\;control\;knockdown-Reading\;for\;{Control}^mcells\;with\;control\;knockdown}\right)\ast100\%$$

### Isobaric tags for relative and absolute quantitation (iTRAQ)

The secretome profiles for cancer cells with high and basal level of miR-125b expression were determined using Click–SILAC method. Briefly, MCF-7 cells transfected with either miR-125b or negative control mimics were seeded at a density of 5.0 x 10^6^ cells in 10-cm cell culture dishes with 10 mL of methionine-depleted RPMI (Thermo Fisher Scientific) supplemented with 10% FBS and 0.1 mM azidohomoalanine (AHA; AnaSpe). After 2 days of incubation, the conditioned media were then harvested and the newly synthesized proteins were coupled to alkyne-functionalized agarose resin by 1,3-cycloaddition using the Click-iT Protein Enrichment Kit (Thermo Fisher Scientific) and the secreted protein were quantified at the Protein and Proteomics Centre at National University of Singapore, using mass spectrometry after on-bead digestion and peptide fractionation.

### Human chemokine proteome profiler

The chemokines secretion profiles for cancer cells with high and basal level of miR-125b expression were determined using Human Chemokine Array Kit (R&D Systems) as per manufacturer’s protocol. Briefly, MCF-7 cells transfected with either miR-125b or negative control mimics were first seeded at a density of 5.0 x 10^6^ cells in 10 cm cell culture dishes with 10 mL of media. After two days of incubation, the conditioned media were then harvested and concentrated using Amicon Ultra 3K Centrifugal Filter Devices (Merck Milipore) by centrifuging at 4,000 *g* for 60 min in a swinging bucket rotor. The soluble protein concentrations in the concentrated media were estimated using Bradford’s assay with BSA as protein standard. Media with equal amount of protein were then mixed with antibody cocktail and loaded onto the array membranes for overnight incubation at 4°C. After several washing steps, the membranes were incubated with secondary antibodies conjugated with horseradish peroxidase (HRP) and were exposed to HRP substrate.

### Testing of neutralizing antibodies with docetaxel

**Fig. 2:**
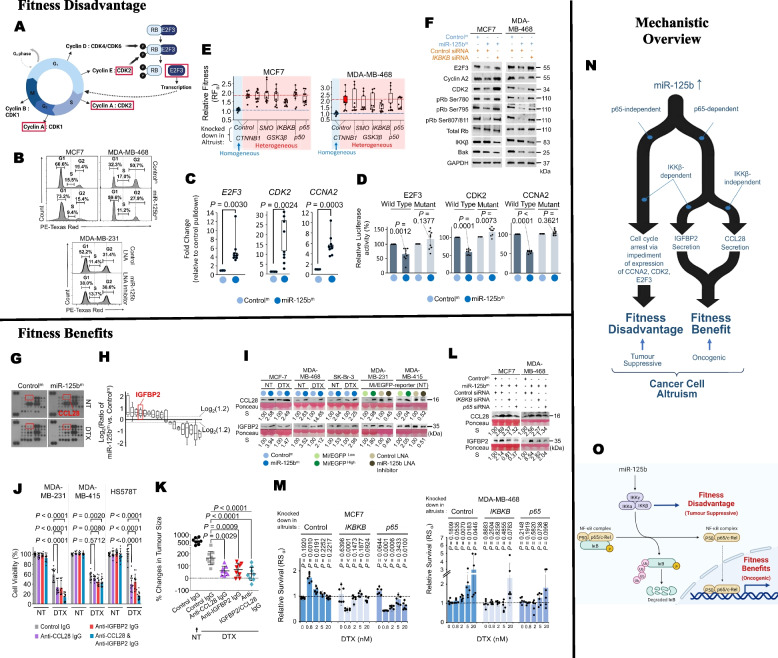
miR-125b dichotomizes via NF-κB signaling into altruistic fitness benefits and disadvantage. **A** Schema showing cell cycle process and regulators. Those indicated with red boxes are involved in G1-S transition and which were selected for further investigation as inhibitory targets of miR-125b. **B** Cell cycle analysis of miR-125b^m^ or Control^m^-transfected MCF7 or MDA-MB-468 cells, or control LNA or miR-125b LNA inhibitor-transfected MDA-MB-231 cells. **C** Box plots of fold change in enrichment of indicated gene transcripts pulled-down using biotinylated miR-125b. **D** Relative luciferase activities of HEK293T cells following transfection of wild-type or mutant reporter construct for indicated genes and mimics. **E** Homogeneous and heterogeneous mixtures of MCF7 (Left) or MDA-MB-468 (Right) were transfected with siRNA against indicated genes, exposed to sublethal 0.8 nM docetaxel, and the RF_α_ measured. Blue or red dotted line/box plot indicate respective levels of RF_α_ for homogenous mixture or heterogenous mixture with control KD. *IKBKB* gene codes for IKKβ. **F** Immunoblotting to detect for indicated proteins extracted from indicated cell lines transfected with combination of indicated mimics and siRNAs for *IKBKB*. **G, H** Conditioned media was harvested from cell culture of miR-125b^m^ or Control^m^-transfected MCF-7 cells and analyzed using cytokine array kit (G) and iTRAQ (H). **I** Immunoblotting to detect CCL28 and IGFBP2 in conditioned media from mimic or LNA inhibitor-transfected, or EGFP reporter-sorted cell fractions of indicated cell lines. Ponceau S was used to visualize protein load. Quantification of band intensities (relative to Control^m^/ Control LNA / Mi/EGFP^Low^) is shown. See Fig. S6F for results of plasmid-transfected MCF7 cells. **J** Viability of indicated cell lines with indicated exposure to docetaxel and/or neutralizing antibodies to IGFBP2 and/or CCL28. **K** Percentage change in size of MDA-MB-231 xenografted tumors in NSG mice with indicated treatment of docetaxel and/or neutralizing antibodies to IGFBP2 and/or CCL28. **L** Immunoblotting to detect for CCL28 and IGFBP2 in conditioned media from indicated cell lines transfected with combination of indicated mimics and siRNA. Ponceau S for protein load normalization. Quantification of band intensities (relative to first band of each set) is shown. **M** RS_α_ of homogeneous and heterogeneous mixture of MCF7 (Left) or MDA-MB-468 (Right) cells transfected with indicated siRNA and exposed to indicated docetaxel concentration. **N** Schema showing proposed mechanism of how miR-125b dichotomizes into fitness benefit and disadvantage of cancer cell altruism. **O** Schematic showing the different components of the NF-κB signaling pathway and how the oncogenic (fitness benefits) and tumor suppressive (fitness disadvantage) events may be mediated by different parts of the same signaling pathway. Experiment repeated two times, representative result shown (B,F,L). Data are mean ± s.d. from three independent biological sets of triplicates (C,D,E,M). Experiment performed once (G), and results validated in (I). Representative blots from three independent replicates (I). Data are mean ± s.d. from three independent biological duplicates (H) or quadruplicates (J). *n* = 8-9 independent animals (K). Statistical analysis was performed using two-tailed one sample *t*-test against 1 (C,M) or 100 (D) or one-way ANOVA with post-hoc Tukey HSD (J,K). NT: no treatment; DTX: docetaxel treatment. Schematic created using Biorender (A,O). Exact *P* values are shown.

MDA-MB-231, MDA-MB-415 and HS578T cells were seeded in 96-well microplates at cell density of 7,000 cells / well in 100 μL culture media and allowed to adhere overnight. Cells were then incubated with antibodies as indicated in Fig. 2F, S6H and S6I for 1 h, followed by addition of different concentrations (0.8 – 5 nM) of docetaxel (Sanofi-Aventis) for 12 h. After 7 days of continuous antibodies incubation, cell viability was determined using the MTS assay. In the control experiments, different concentrations of immunoglobulin G (IgG) isotype control (Cell Signaling Technology) of matched animal origin were added in combination with docetaxel.

### Enzyme-linked immunosorbent assay (ELISA)

The concentrations of IGFBP2 and CCL28 in the plasma samples of breast cancer patients were quantified using Human IGFBP2 ELISA kit (ab100540; Abcam) or CCL28 ELISA kit (ab99988; Abcam). For each breast cancer patient, serial blood samples were collected during each routine clinical follow-up and were centrifuged at 3,000 *g* for 10 min to separate out the plasma for collection. Then, 100 μL of the collected plasma samples was loaded onto pre-coated 96-well ELISA plates and the ELISA was carried out according to the manufacturer’s instructions. Both sampling and study protocols were approved by the Institutional Review Board (IRB) committee of the National University of Singapore (NUS).

### Western immunoblotting

Conditioned media or whole cell lysate were fractionated by SDS-PAGE and transferred to a nitrocellulose membrane using a Trans-Blot® Turbo (Biorad) according to the manufacturer’s protocols. Transferred protein was visualized by staining membrane with Ponceau S (Sigma-Aldrich), and subsequently washed away using distilled water. After incubation with 5% non-fat milk in TBS-T (10 mM Tris, pH 8.0, 150 mM NaCl, 0.5% Tween 20) for 60 min, the membrane was washed once with TBS-T and incubated with antibodies against IGFBP2 (polyclonal; 1:1000; Cell Signaling Technology), CCL28 (polyclonal; 1:500; Abcam), Bak1 (1:1000; Cell Signaling Technology), Cas9 (1:1000; Active Motif), KLF2 (1:1000; Santa Cruz), GAPDH (1:1000; Santa Cruz), CCNA2 (1:1000, Cell Signaling Technology). CDK2 (1:2000, Cell Signaling Technology), E2F3 (1:1000, Santa Cruz), GAB1 (1:1000, Cell Signaling Technology), AKT(pan) (1:3000, Cell Signaling Technology), Phospho-AKT(Ser473) (1:1000, Cell Signaling Technology), Phospho-PI3 Kinase p85 (Tyr458)/p55 (Tyr199) (1:1000, Cell Signaling Technology), PI3 Kinase p85α (1:1000, Cell Signaling Technology), Phospho-Rb (Ser780) (1:1000, Cell Signaling Technology), Phospho-Rb (Ser795) (1:1000, Cell Signaling Technology), Phospho-Rb (Ser807/811) (1:1000, Cell Signaling Technology), IKKb (1:1000, Santa Cruz), Rb (1:2000, Santa Cruz) or RELA/NFκB p65 (1:1000, Santa Cruz) at 4 °C for 12 h. Membranes were washed three times for 10 min and incubated with a 1:2000 dilution of HRP-conjugated anti-mouse (Santa Cruz) or anti-rabbit antibodies (Santa Cruz) for 2 h. Blots were washed with TBS-T three times and developed with the ECL system (Amersham Biosciences) according to the manufacturer’s protocols. ImageJ was used to quantify intensity of bands for immunoblots.

### FACS analysis

For isolation and/or detection of miR-125b promoter-driven EGFP signals, adherent MDA-MB-231^*miR-125bprom-EGFP*^, HS578T^*miR-125bprom-EGFP*^ or MDA-MB-415^*miR-125bprom-EGFP*^ cells were harvested with 0.05% trypsin. FACS analysis was performed at (i) Flow Cytometry Laboratory at Life Science Institute (Singapore) on MoFlo Legacy cell sorter (for isolation of HS578T^*miR-125bprom-EGFP*^ or MDA-MB-415^*miR-125bprom-EGFP*^; Beckman Coulter), BD LSRFortessa X-20 Cytometer (for detection of EGFP in various cell lines, BD Biosciences) and Attune NxT Flow Cytometer (for cell enumeration of various cell lines, Thermo Fischer Scientific), and (ii) Flow Cytometry Laboratory Unit at Yong Loo Lin School of Medicine, National University of Singapore on BD FACSVantage SE (for isolation of MDA-MB-231^*miR-125bprom-EGFP*^; BD Biosciences) and BD LSRFortessa X-20 Cytometer (for detection of EGFP in various cell lines, BD Biosciences). Sorted cells were visually inspected under microscope and cultured for a short time to exclude disproportional enrichment of debris or apoptotic cells.

For cell cycle analysis, cells were collected 48 h post-transfection, washed with PBS and fixed in 70% ethanol overnight at 4°c. The next day, cells were washed with PBS, stained with a solution comprising PBS, 0.1% Triton-X (Sigma), 0.2mg/mL RNAse A (Qiagen) and 20ug/mL PI (Sigma), and incubated at 37 °C for 15 min. Cells were then analyzed on a BD Fortessa flow cytometer (Becton Dickinson).

### Chromatin immunoprecipitation (ChIP)

ChIP was performed using ChIP-IT® Express Enzymatic kit (Active Motif) as per manufacturer’s protocols, using 1-3 μg of antibodies against histone H3ac (Active Motif), histone H4ac (Active Motif), PCAF (Cell Signaling Technology), KLF2 (Santa Cruz), RNA polymerase II (Active Motif) or negative control IgG (Active Motif). Eluted DNA was used as a template in PCR. Image J was used to quantify intensity of for PCR bands as visualized using agarose gel electrophoresis. Sequences for ChIP primers are as follows:

#### For KLF2 Binding site 1 (KBS-1; Fig. S9D-E)

Forward 5′-CCTCCACTCCCACCCAACTG-3′

Reverse 5′-GCTGGGCGAGGTGTTTCAATA- 3′

These primers were used to amplify a 146-base pair fragment.

#### For KLF2 Binding site 2 (KBS-2; Fig. S9D)

Forward 5′- AGGTGTGATGATGACAGACTAGC-3′

Reverse 5′- CACCTCTGTGGGTTCTTCTCA- 3′

These primers were used to amplify a 116-base pair fragment.

#### For TSS proximal site (Fig. S9B)

Forward 5′- GCCCAAATCTTGAAAGAGTTTTCT-3′

Reverse 5′- ACATACGCAGTATCTGGGGG – 3′

These primers were used to amplify a 90-base pair fragment.

#### For site distal to promoter of hsa-miR-125b-1 (Fig. S9B,D,E)

Forward 5′- TAGTCACCAACTCCTGGCAAC-3′

Reverse 5′- CTGACTGTGAGACCTGCAGAA- 3′

These primers were used to amplify a 233-base pair fragment.

For KBS-1 and KBS-2, the primers were designed (i) to amplify part of the promoter that encompasses the binding site itself (e.g. KBS-2), or (ii) to lie within ±200-bp from the binding site under study (e.g. KBS-1, when it is not possible to design primers to successfully amplify region of the promoter satisfying (i) ). The size of the smallest and most abundant fragments produced by the enzymatic shearing method employed herein is ~ 200-bp, thus making it essential to design primer sites that lie within 200-bp from the antibody-targeted site.

### mRNA:miRNA Pull-Down

MCF7 cells were seeded into 6-well cell culture plate with a density of 200,000 cells/well. Cells cultured in antibiotic-free RPMI were transfected using Lipofectamine 3000 (Thermo Fisher Scientific) with 75 picomoles of biotinylated miRCURY LNA Premium miRNA mimics of *hsa-miR-125-5p* (Qiagen, YM00473299-BDI) and negative control (Qiagen, YM00479903-BDI). RNA was extracted using RNeasy Plus Mini Kit (Qiagen) 48 h post-transfection. Pull-down of target mRNA:miRNA complexes was performed as described by Dash *et. al* [36]. Paired-end 150 cycles next-generation sequencing was performed on NovoSeq 6000 (Illumina) at Novogene AIT (Singapore) or Azenta Life Sciences (United States). BAM files generated by aligning FASTQ files to Homo sapiens hg38 Genome Reference were subject to RNA-Seq Analysis workflow using CLC Genomics Workbench 20.0.4 (Qiagen).

### Conditioned medium experiment

MDA-MB-231^miR-125b-prom^ and MDA-MB-415^miR-125b-prom^ cells were sorted into Mi/EGFP^High^ and Mi/EGFP^Low^ fractions and seeded at 200,000 cells per well on a 24-well plate. Conditioned media were harvested 2 days later. 20,000 freshly sorted Mi/EGFP^High^ and Mi/EGFP^Low^ cells were then seeded on a 24-well plate with the conditioned medium, in the presence of different combinations of treatments involving chemical inhibitors (LY294002, 5μM or Akt Inhibitor IV, 200nM) or recombinant proteins (CCL28 or IGFBP2, 250ng/mL). The cells were then harvested after 3 days (for MDA-MB-231) or 4 days (for MDA-MB-415) for flow-cytometry analysis (Becton Dickinson) or western immunoblotting.

For the experiment investigating the role of GAB1, 20,000 freshly sorted cells were first transfected with control siRNA or *GAB1* siRNA for 4 h before the medium was replaced with conditioned medium, in the presence of either chemical inhibitors or recombinant proteins as mentioned above.

### CRISPR interference (CRISPRi)

Plasmid lentiviral sgRNA/dCas9 constructs were custom-made by Cellecta. Sequences of the sgRNA inserts within the construct pRSGdCCN-U6-sg-CMV-dCas9-2A-Neo are as follows:

- *To target part of KLF2 binding site 1:*
  - Sequence (1) 5’-TAGAGGACGGAGAACGGGGG-3’ (Sequence underlined is part of KLF2 binding site 1)
  - Sequence (2) 5’-GAGGACGGAGAACGGGGCG-3’ (Sequence underlined is part of KLF2 binding site 1)
- *To target part of KLF2 binding site 2:*
  - Sequence (3) 5’-AGTTGTCTTGAAGGTGGGGG-3’ (Sequence underlined is part of KLF2 binding site 2)
  - Sequence (4) 5’-TCTTGAAGGTGGGGGTGGGG-3’ (Sequence underlined is part of KLF2 binding site 2)
- *Control construct:*
  - Sequence (5) 5’-AAGATCGAGTGCCGCATCAC-3’ (Sequence target copGFP)

**Fig. 3:**
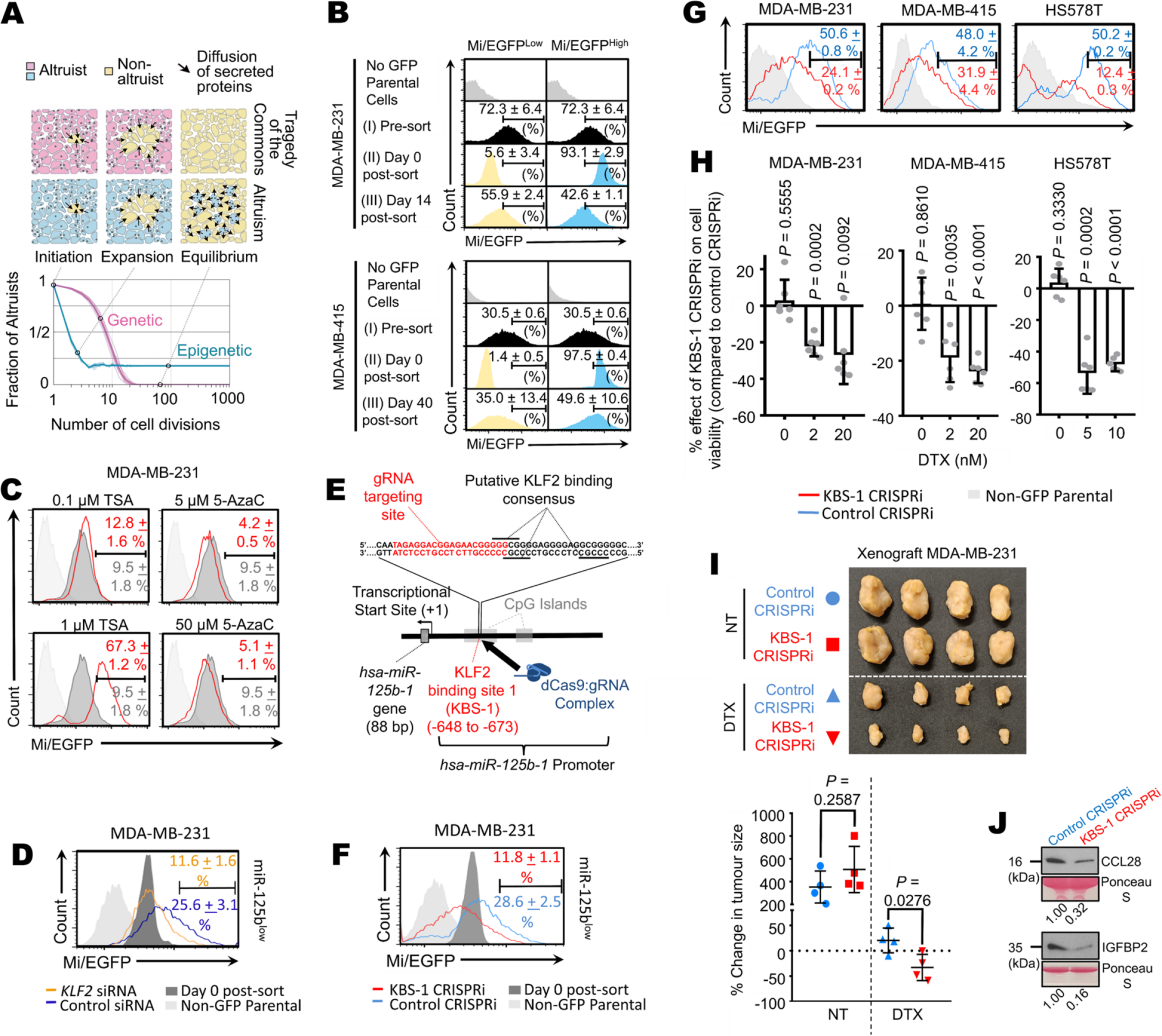
Altruistic cancer cells regenerate via epigenetic mechanism. **A** Mathematical model explains the persistence of altruistic subclone in breast cancer cell population (Upper), and a spatial model simulates changes in the percentage of altruistic cells over time for hypothesized genetic- and epigenetics-mediated altruism (Lower). Dotted lines link events in upper panel to corresponding points in lower panel. See Supplementary Note 2 & 3. **B** Indicated cancer cell lines were sorted according to Mi/EGFP levels and the fluorescence monitored over indicated time. **C** Mi/EGFP fluorescence of non-sorted MDA-MB-231^miR-125bprom-EGFP^ cells, treated as indicated, as determined by FACS. **D** Mi/EGFP^Low^ cells were transfected with indicated siRNAs and the Mi/EGFP fluorescence determined after four days. **E** Schema showing putative consensus sites for KLF2 binding along the *hsa-miR-125b-1* promoter and gRNA targeting sequence of CRISPRi. **F** Mi/EGFP^Low^ cells were transduced with lentivirus for expression of CRISPRi targeting KBS-1 or non-specific sequence, and Mi/EGFP fluorescence determined after four days. **G,H** Changes in Mi/EGFP fluorescence as treated in (F) were monitored in indicated cell lines (G). Percentage effect of KBS-1 CRISPRi expression on cell viability relative to control CRISPRi was also measured. Cancer cells were exposed to indicated concentration of docetaxel (H). **I** MDA-MB-231^miR-125bprom-EGFP^ cells, as treated in (F), were grown as xenografts in NSG mice. Upper: excised tumors at end point of monitoring period. Lower: Percentage change in tumor size with and without docetaxel treatment. **J** Immunoblotting to detect for IGFBP2 and CCL28 in conditioned media from MDA-MB-231^miR-125bprom-EGFP^ cells as treated in (F) and used to establish xenograft model for (I). Ponceau S was used to visualize protein load. Quantification of band intensities (relative to Control CRISPRi) is shown. Experiments repeated two times (B, C, D, F, G, H, J). Representative data are shown for (J). Mean percentage ± s.d. cells for technical triplicates of representative set are shown in same colours as corresponding histograms (C, D, F, G) or in black only (B). Data are mean ± s.d. from two independent biological sets of triplicates (H). *n* = 4 independent animals per group (I). Statistical analysis was performed using two-tailed one sample *t*-test against 0 (H) and two-tailed unpaired *t*-test (I lower). NT: no treatment; DTX: docetaxel treatment. Exact *P* values are shown

HEK293FT (Thermo Fisher Scientific) cells were seeded on 10-cm plates till 80% confluency was reached. Transfection was performed using 3 μg of the lentiviral constructs, 9 μg of ViraPower packaging mix (Thermo Fisher Scientific) and 36 μL of Lipofectamine 3000 (Thermo Fisher Scientific). The plates were left overnight at 37°C in 5% CO_2_ before the culture medium was replaced with fresh medium. The eventual virus-containing medium was harvested 48 h post-transfection and subjected to centrifugation at 3,000 rpm for 5 min to remove cell debris. The medium was then passed through a 0.45 μM filter (Sartorius) and added to cells seeded in 6 well plates, supplemented with 6 μg/mL Polybrene (Sigma-Aldrich). Two rounds of lentiviral transduction were performed. The effective MOI for CRISPR inhibition was determined to be 25 for MDA-MB-231 and HS578T, and 50 for MDA-MB-415. Four days later, transduced cells were analyzed using FACS. Transduced cells were then expanded and harvested for multiple experiments, not exceeding more than 5 passages from the lentiviral transduction stage. ChIP was performed as described above, using antibodies against KLF2 (Santa Cruz), cas9 (Active Motif) or negative control IgG (Active Motif), and PCR of the ChIP eluate performed for KLF2 Binding site 1. Results show binding of target-specific dCas9 to KLF2 binding site 1, with reduced binding of KLF2 (Fig. S9E). The percentage effect due to KBS-1 CRISPRi expression (Fig. 3H) and docetaxel treatment is calculated using the formula: $\lbrack\left(\text{viability of cells with KBS-1 CRISPRi - viability of cells with control CRISPRi}\right)/\text{viability of cells with control CRISPRi}\rbrack\ast100\%$.

### ChIP-Seq

ChIP-Seq was performed using MDA-MB-231 cells transduced with lentivirus containing the control or KBS1-targeting CRISPRi construct. ChIP was performed using ChIP-IT® Express Enzymatic kit (Active Motif) as per manufacturer’s protocols, using 1-3 μg of antibody against dCas9 (Active Motif). Eluted DNA was used as a template for ChIP-Seq. Eluted DNA was used to prepare libraries using the TruSeq ChIP Library Preparation kit (Illumina). Sequencing was performed on an Illumina HiSeq platform with 101-bp paired-end reads (Macrogen). Our results demonstrate binding of dCas9 protein to region of miR-125b’s promoter harboring KLF2- binding site 1 (Fig. S9F).

### Single cell RNA Sequencing

MDA-MB-231^miR-125bprom-EGFP^ cells were sorted according to Mi/EGFP levels using maximum and minimum thresholds set at 10%. Cells were immediately lysed and whole-transcriptome amplified using SMARTer Ultra Low RNA kit for Illumina (Clontech). Single-cell cDNA libraries were constructed with NEBNext Ultra RNA Library Prep Kit for Illumina (NEB, USA) according to manual instructions and sequenced on an Illumina HiSeq 2000. Reads containing adapter or low-quality sequences were removed, and the quality of the reads was determined by their error rate, Q20, Q30, and GC-contents. The clean reads were mapped with the TopHat package (v.2.1.1) against the UCSC hg19 human DNA reference. Alignments were sorted with Integrative Genomics Viewer (IGV v.2.4). The read counts of each gene were summarized by HTSeq v0.6.1 and DESeq2 R package (v.3.7) was used to detect differentially expressed genes (DEGs). Significant differentially expressed genes between MI/EGFPHigh and MI/EGFPLow cells were selected using the following criteria: |log2 (Fold change)|≥ 2 and adjusted P-values ≤ 0.05. Gene ontology (GO) enrichment analysis of DEGs was performed using Cytoscape plugin ClueGO (v.2.5.1). GO terms with a Benjamini-Hochberg false discovery rate (FDR) corrected P-values ≤ 0.05 were considered significantly enriched by DEGs. The principal component analysis (PCA) and hierarchical clustering were performed using ClustVis.

### Differentially expressed genes (DEGs) between miR-125b^High^ vs miR-125b^Low^ breast cancer cells

Significant DEGs (FDR<0.05) were determined for miR-125b^High^ vs miR-125b^Low^ fractions of four different breast cancer cell lines. Significant DEGs (FDR < 0.05) were also determined for ER+ breast cancer tissue vs corresponding normal adjacent tissue, and triple negative breast cancer (TNBC) tissue vs corresponding normal adjacent tissue in the public dataset GSE58135. The DEG status of all genes were classified as “not sig” for no significance in the comparison, “high” for over-expression and “low” for under-expression in tumor versus normal adjacent control or miR-125b^High^ vs miR-125b^Low^. The DEG status of the cell lines were then compared to the corresponding tumor comparison using a Chi-square test to identify if there is an association of the DEG states between the two comparisons. Bubble plots were then plotted using R version 4.2.1 using the package ggplot2. Heat maps of the DEGs were plotted using the ComplexHeatmap package in R version 4.2.1.

### Mouse xenograft experiments

#### To monitor EGFP expression with or without docetaxel treatment

After typsinization, MDA-MB-231^miR-125b-prom-GFP^ cells were counted and resuspended in PBS. Subcutaneous injections of the cells were performed at the fourth inguinal mammary region of 6-8 weeks old female NOD.CB17-Prkdc^scid^ Il2rg^tm1Wjl^/SzJ (NSG) mice (Invivos Singapore). Each mouse was inoculated with 1.5x10^6^ cells in a final volume of 100 μL. Upon reaching the size of about 100 mm^3^, docetaxel (10mg/kg; Sanofi-Aventis) or vehicle PBS was injected intra-peritoneally every 2 days for 4 times. At the end of the experiment or when the tumors reached 10% of body weight, the mice were euthanized and the xenograft tissue harvested.

The harvested tissues were washed three times with PBS and fixed in 4% paraformaldehyde (Sigma-Aldrich) overnight. Fixed tissues were subjected to processing and then embedded in paraffin wax. The resulting FFPE specimens were sectioned into 5 μM slices. Sections were used for immunohistochemical detection for Mi/EGFP expression. Heat-induced epitope retrieval was performed with 10 mM citrate buffer (pH 6) (Sigma-Aldrich). Sections were incubated overnight at 4°C with 1:50 anti-GFP antibody (Cell Signaling Technology) and developed using the EnVision G|2 Doublestain System kit (Dako) as per manufacturer’s protocol.

#### To derive “Relative Survival” and “Relative Fitness”

MCF-7-luc-F5 cells stably expressing the luciferase gene (MCF-7^luc^) and MCF-7^luc^ stably expressing GFP MCF-7^luc+GFP^) were used. MCF7^luc^ cells were transfected with either control or miR-125b mimics, whereas MCF7^luc+GFP^ cells were transfected with control mimics using Lipofectamine 3000 (Thermo Fisher Scientific). The combination of MCF7^luc^ and MCF7^luc+GFP^ would allow for discrimination of the cells by fluorescence in subsequent analysis. The transfected MCF-7 cells were then mixed in 1:1 ratio in two different combinations: control mimic-transfected MCF-7^luc^/ control mimic-transfected MCF-7^luc+GFP^ (the “homogeneous” mixture) or a miR-125b mimics-transfected MCF-7^luc^ /control mimics-transfected MCF-7^luc+GFP^ (the “heterogeneous” mixture). Subcutaneous injections of the cell mixture were performed at the fourth inguinal mammary region of 6-8 weeks old female NSG mice (Invivos Singapore). 17 β-estradiol pellets (0.72mg dose, 90-day release) (Innovative Research of America) were subcutaneously implanted into the flanks of each mouse at least 2 days prior to the start of treatment regimen. Each mouse was inoculated with 1x10^6^ cells in a final volume of 100 μL, in a 1:1 mix with ECM gel (Sigma-Aldrich). Sub-lethal dose of docetaxel (1mg/kg, Sanofi-Aventis) or vehicle PBS was injected intra-peritoneally for 2 consecutive days on 4^th^ and 5^th^ day post-xenotransplantation – this sub-lethal dose was used to ensure subsequent regrowth of docetaxel-treated tumors, which could then be harvested to determine the relative proportion of EGFP vs. non-EGFP cells for determination of Relative Fitness. Treatment at early stage of xenograft implantation also ensures that transfected mimics (miR-125b or control mimics) within the injected cancer cells would not be markedly diluted due to cell divisions. Use of higher concentration of docetaxel (e.g. 10 mg/kg) for early tumor implants led to complete regression of xenografted tumor and made it impossible to assess Relative Fitness. To derive the percentage change in tumor size (Fig. 1N) and Relative Survival (Fig. 1O), photon counts indicating tumor size were monitored on day of first treatment, and 2 days after last treatment, using fluorescence imaging on the IVIS Spectrum imaging machine (Perkin Elmer), with intraperitoneal injections of d-Luciferin (Promega). For three of the six mice injected per treatment group, the tumor was harvested 2 days after docetaxel exposure. The harvested tissues were washed three times with PBS and then disaggregated using 5mg/mL Collagenase IV (Thermo Fisher Scientific) in HBSS for 2 h at 37°C. The harvested cells were then subjected to FACS analysis using Attune NxT Flow Cytometer, to determine the proportion of GFP-tagged Control^m^ vs. non-tagged miR-125b^m^ MCF-7 cells. APC-tagged human CD24 antibody (Biolegend) was added to select for human cells during FACS analysis. The ratio of GFP-tagged : non-tagged cells in the “homogeneous” mixture was normalized to 1:1, and the same normalization factor was applied to the corresponding “heterogeneous” mixture for each treatment group. This data, together with the photon counts obtained 2 days post-treatment, were used to derive the Relative Survival for MCF-7 xenograft study. The number of Control^m^ cells in each xenograft tumor is estimated using the following formula: (proportion of Control^m^ cells as estimated using FACS x photon counts 2 days post-treatment). RS_α_ was then calculated using the following:

$${\mathrm{RS}}_{\mathrm\alpha}=\frac{proportion\;of\;{Control}^m\;cells\;in\;heterogeneous\;xenograft\;x\;photon\;counts\;2\;days\;post-treatment\;of\;heterogeneous\;xenograft}{proportion\;of\;{Control}^m\;cells\;in\;homogeneous\;xenograft\;x\;photon\;counts\;2\;days\;post-treatment\;of\;homogeneous\;xenograft}$$

At the end of 8 weeks or when the tumors reached 10% of body weight, the mice were euthanized and the xenograft tissue harvested for further analysis. The harvested xenograft tissues were washed three times with PBS and fixed in 4% paraformaldehyde (Sigma-Aldrich) overnight. Fixed tissues were subjected to tissue processing at the Department of Pathology, NUS. The resulting FFPE specimens were sectioned into 7 μM slices, that were adhered to glass slides and deparaffinized in three consecutive xylene baths for 5 min each, followed by 1 min each in serial dilutions of ethanol (99.9%, 96%, 70%) and one change of PBS. Heat-induced epitope retrieval was performed using a 10 mM citrate buffer (pH 6) (Sigma-Aldrich) using a pressure cooker. Consecutive sections were incubated overnight at 4°C with either 1:50 dilution of anti-GFP antibody (Cell Signaling Technology) or 1:200 dilution of anti-luciferase antibody (Novus Biologicals), developed using the EnVision G|2 Doublestain System kit (Dako) as per manufacturer’s protocol and counterstained using haematoxylin. Images of the slides were taken using the Nikon Eclipse TS100 microscope (Nikon) and image acquisitions were made using the NIS-Elements software equipped with DS-FI1C camera (Nikon). We selected regions of consecutive sections where all cells were observed to be brown (for luciferase, due to staining with DAB+) for further analysis. To determine the number of cells with pink (for EGFP, due to staining with Permanent Red) and/or purple (for all cells, due to H&E staining), we used Fiji. Briefly, each image was decomposed into red (for GFP staining) and blue (for H&E staining) channel using the “color deconvolution” ⇨ “H&E DAB” option. The threshold for the red and blue channels was adjusted to values of 70 (blue) and 170 (red). The “Analyze Particle” option was then activated, and the particle size limit indicated as 300 (for blue channel) and 150 (for red channel). This gave us the total number of cells (blue channel) or number of GFP-tagged cells (red channel) in regions of the sections selected for analysis. The number of non-GFP cells was derived by deducting the number of GFP cells from total number of cells detected. The ratio of GFP : non-GFP cells in “homogeneous” mixture was normalized to 1:1, and the same normalization factor was applied to the corresponding “heterogeneous” mixture for each treatment group. RF_α_ was then calculated using the following:

$${\mathrm{RF}}_{\mathrm\alpha}=\frac{Normalized\;number\;of\;GFP\;cells\;in\;the\;heterogeneous\;xenograft}{Normalized\;number\;of\;non-GFP\;cells\;in\;the\;heterogeneous\;xenograft}$$

#### For xenograft experiments to study “Fitness Disadvantage”

MCF-7-luc-F5 cells stably expressing the luciferase gene (MCF-7^luc^) and MCF-7^luc^ stably expressing GFP (MCF-7^luc+GFP^) were used. MCF7^luc+GFP^ cells were transfected with mimic/siRNA in three different combinations (control mimic with control siRNA, miR-125b mimic with control siRNA, miR-125b mimic with IKBKB siRNA), and then mixed in 1:1 ratio with MCF7^luc+GFP^ transfected with control mimic and control siRNA.

Subcutaneous injections of the cell mixture were performed at the fourth inguinal mammary region of 6-8 weeks old female NSG mice (Invivos Singapore). 17 β-estradiol pellets (0.72mg dose, 90-day release) (NE-121; Innovative Research of America) were subcutaneously implanted into the flanks of each mouse at least 2 days prior to the start of treatment regimen. Each mouse was inoculated with 1x10^6^ cells in a final volume of 100 μL, in a 1:1 mix with ECM gel (Sigma-Aldrich). Sub-lethal dose of docetaxel (1mg/kg, Sanofi-Aventis) was injected daily for 2 consecutive days and intra-peritoneally from the 4^th^ day post-xenotransplantation. At the end of 8 weeks or when the tumors reached 10% of body weight, the mice were euthanized and the xenograft tissue harvested for further analysis, as per the protocol for xenograft experiments to derive “Relative Fitness”.

#### To monitor EGFP expression of xenograft established from purified miR-125b^High^ and miR-125b^Low^ MDA-MB-231^miR-125b-prom-GFP^ cells

Sub-lethal dose (2 nM) docetaxel-treated MDA-MB-231^*miR-125bprom-EGFP*^ cells were sorted according to EGFP levels using maximum and minimum thresholds set at 10% and resuspended in PBS. Subcutaneous injections of the cells were performed at the fourth inguinal mammary region of 6-8 weeks old female NSG mice (Invivos Singapore). Each mouse was inoculated with 1.5x10^6^ cells in a final volume of 50 μL. Changes in tumor size were monitored via physical measurement using vernier calliper. After tumor size reached 150 mm in diameter (~8 weeks after inoculation of mice with cells), tumors were excised from mice for further analysis.

The harvested tissues were washed three times with PBS and then disaggregated using 5mg/mL Collagenase IV (Thermo Fisher Scientific) in HBSS for 2 h at 37°C. Released cells were collected, subjected to centrifugation at 2,000 rpm for 10 min at 4°C, seeded for 24-h before being resuspended in 300 μL of PBS for FACS analysis using X-20 Cytometer (BD Biosciences) for detection of EGFP.

#### To study the effect of CRISPRi with or without docetaxel treatment

MDA-MB-231^miR-125b-prom-GFP^ cells transfected with either control CRISPRi or CRISPRi targeting KBS-1 were counted and resuspended in PBS. Subcutaneous injections of the cells were performed at the fourth inguinal mammary region of 6-8 weeks old female NSG mice (Invivos Singapore). Each mouse was inoculated with 4x10^6^ cells in a final volume of 100 μL. Upon reaching the size of about 100 mm^3^, 10 mg/kg dose docetaxel (Sanofi-Aventis) or vehicle PBS was injected intra-peritoneally every 2 days for 8 times. Changes in tumor size were monitored via physical measurement using vernier calipers. When tumors reached 1.5 cm in length, the mice were euthanized and the xenograft tissue harvested.

#### For all mice xenograft experiments

Protocols for *in vivo* experiments were approved by Institutional Animal Care and Use Committee (IACUC) at the National University of Singapore (protocol number 009/12 & R16-0019). All animals were maintained in accordance to IACUC guidelines. Xenografted tumors were allowed to grow to a maximum size of 150 mm in diameter and in none of the experiments were these limits exceeded.

### Correlation of gene expression

Data from GEO Dataset GSE58212 consisting of bot mRNA and microRNA expressions was downloaded and analyzed. Specifically, the correlation between the co-expression of miR-125b or *IKβKβ* with the three cell cycle regulators (*CCNA2, CDK2, E2F3*) was determined by calculating the Pearson correlation coefficients.

### Survival analysis

Survival was estimated by the Kaplan–Meier method using Kaplan Meier plotter (www.kmplot.com). For Fig. S3A-D, analyses were limited to breast cancer patients with chemotherapy-treated (and without endocrine therapy) (Fig. S3A,B) or with no treatment (Fig. S3C,D) from the METABRIC dataset. For Fig. S3E-H, Gene ID 202718_at (IGFBP2) and 224240_s_at, 238750_at (CCL28) were selected for analyses, that were limited to breast cancer patients with neoadjuvant treatments (and without endocrine therapy), from the following datasets from GEO and EMBL-EBI: GSE16391, GSE16446, GSE31519, GSE20194, GSE20271, GSE32646, GSE18728, GSE23988, GSE41998, GSE16716, E-TABM-43, GSE37946, GSE4611, GSE25066, GSE22093 and GSE50948. For Fig. S3I, analysis was limited to breast cancer patients with docetaxel chemotherapy from the TCGA-RPPA dataset. Survival curves were generated, along with log-rank *P* values and hazard ratios with 95% confidence intervals.

Patients who had undergone endocrine therapy were excluded from our analyses. We observed that stratification survival analyses for breast cancer patients treated with endocrine therapy but not chemotherapy did not reach statistical significance (for OS & DMFS for IGFBP2) or showed a reverse trend (high CCL28 expression correlates with longer DMFS) (Results not shown).

### Gene expression profiling using microarrays and data analysis

MDA-MB-231^miR-125bprom-EGFP^ and MDA-MB-415^miR-125bprom-EGFP^ cells were sorted for lowest, middle or highest 10% Mi/EGFP levels to derive the Mi/EGFP^Low^, Mi/EGFP^Mid^ and Mi/EGFP^High^ fractions respectively. RNA extraction was performed on Days 0 and 14 for MDA-MB-231^miR-125bprom-EGFP^cells, and Days 0 and 40 for MDA-MB-415^miR-125bprom-EGFP^ cells. Gene expression profiling was performed at Research Support Centre (RSC), Agency of Science, Technology and Research (A*STAR) using GeneChip Human Genome U133 Plus 2.0 (Affymetrix). Gene expression data were normalized, and PCA plots were analyzed and visualized using the Transcriptome Analysis Console (TAC) software (ThermoFisher Scientific).

### Data collection

Power calculation was used to determine the sample size for breast cancer biopsies for miRNA *in-situ* hybridization, with the effect size estimated based on proportion of patients with CTCs showing miR-125b expression during taxane chemotherapy. For other experiments, no statistical method was used to pre-determine sample size. The number of mice was determined based on previous experiences working with breast cancer xenograft mice models and other published literatures. The experiments were not randomized, and the investigators were not blinded to allocation during experiments and outcome assessment, unless otherwise stated.

### Statistics

No samples or animals were excluded from analysis, sample size estimates were not used, and replicate measurements were taken from distinct samples. For xenograft mice experiments, animals were randomly assigned into a treatment group with the constraint that the starting tumor burden in the treatment and control groups was similar. Comparisons between two groups were performed using a two-tailed Mann–Whitney U-test (unpaired samples), two-tailed Wilcoxon signed rank test (paired samples), one sample *t*-test and two-tailed Student’s *t-*test. Multiple samples were compared using one-way ANOVA with post-hoc Tukey HSD correction for multiple comparisons. Survival curves were compared using a log-rank test and hazard ratios with 95% confidence intervals (generated via www.kmplot.com). Categorical variables were compared using a χ2 test. Pearson’s correlation was calculated to study the association between staining intensities. Linear and polynomial (quadratic) regression analyses were performed to determine the best-fit shape of the percentage benefit–percentage altruist relationship for Fig. S6A. Data analysis was performed using XLSTAT for Excel, R programming and online statistical calculator such as:

- https://www.danielsoper.com/statcalc/calculator.aspx?id=98,
- http://www.statskingdom.com/180Anova1way.html
- http://astatsa.com/OneWay_Anova_with_TukeyHSD/.

Studies were not conducted blinded except for analysis of miR-125b staining in FFPE breast cancer biopsies, and immunohistochemical analysis of EGFP vs. luciferase in xenograft tumors.

## Results

### Breast cancer cells exhibit altruistic cooperation

We began our study by identifying microRNA (miRNA) biomarkers indicating emergence of chemo-refractoriness. Analysis of circulating tumor cells (CTCs) was used as a means to monitor dynamic changes in tumor clonal composition during therapy. In longitudinal monitoring of miRNA expression profiles of CTCs from breast cancer patients undergoing taxane-based chemotherapy, we observed moderate to high miR-125b expression in 7/10 cases of different breast cancer subtypes, monitored over 2-13 months (Fig. S1A-J, Table S1). Our observations appeared to contradict previous reports of miR-125b being down-regulated in breast cancer tissue as compared to normal adjacent tissue [37, 38]. We thus hypothesized that miR-125b expression was increased in breast cancer cells specifically in response to taxane exposure. Consistent with this hypothesis, we observed that significantly more breast cancer patients with taxane-based neoadjuvant chemotherapy showed heightened miR-125b expression, as compared to patients without (Fig. 1A, Table S2). We also demonstrated that docetaxel, a taxane agent in clinical use, increased miR-125b expression and promoter activity in adherent (Fig. 1B, Fig. S2A), spheroidal (Fig. S2B) and tumor xenograft (Fig. S2C) forms of the endogenously high miR-125b-expressing human breast cancer cell line MDA-MB-231^miR-125bprom-EGFP^, which harbors a lentiviral construct that expresses EGFP, driven by the *hsa-miR-125b-1* promoter sequences (abbreviated Mi/EGFP; miR-125b expression is shown to correlate with Mi/EGFP level; Fig. S2D).

Notably, we found that miR-125b was highly expressed in only a minority of breast cancer cells even after taxane treatment (Fig. 1, B-C, Fig. S2A-D). Overexpression of miR-125b has been shown to confer treatment resistance to cancer cells [35, 39] and correlates with disease progression in neoadjuvant therapy-treated breast cancer patients [40, 41] and with shorter overall survival (OS) in chemotherapy-treated patients (Fig. S3A-D). This raised the question of why the heterogeneous tumor population would not become enriched with cells high in miR-125b expression (miR-125b^High^) post-treatment: the apparently obvious advantage that resistance confers to this subpopulation should enable it to spread and dominate, given the traditional “survival of the fittest” paradigm of clonal selection. To answer this question, we used low miR-125b-expressing human breast cancer cell lines (MCF-7, SK-BR-3, MDA-MB-468; Fig. S2F) and reconstituted this heterogeneity *in vitro* by mixing miR-125b mimic-transfected (miR-125b^m^) cells with control mimic-transfected (Control^m^) cells in a 1:1 ratio (“heterogeneous” mixture). One of the two fractions was tagged with fluorescent protein to track the origin of the daughter cells post-treatment. In the control experiment (“homogeneous” mixture), all cells were transfected with control mimic, with half of the population being fluorescence-tagged (Fig. 1D). With docetaxel treatment, we hypothesized that: (i) the “heterogeneous” mixture would survive better than the “homogeneous” mixture during early post-treatment, and (ii) progenies from miR-125b^m^ cells would dominate the population after allowing the survivors of the “heterogeneous” mixture to recover from drug treatment and regrow to confluency.

In our study, we observed only (i) to be true (Fig. 1E-G, Fig. S4A-C). Notably, the partner Control^m^ cells in “heterogeneous” mixture survived significantly better than their Control^m^ counterparts in the “homogeneous” mixture (Relative Survival, RS_α_ >1; Fig. 1H-J). However, unexpectedly and contrary to our hypothesis in (ii), we observed in the “heterogeneous” mixture that it was the Control^m^ cells that dominated the regrown population while miR-125b^m^ cells showed reduced fitness, as defined by the proportion of cell progenies that a specific fraction could give rise to (Relative Fitness, RF_α_ >1; Fig. 1 K-M). We observed similar trends for murine xenografts, with “heterogeneous” tumors showing a survival advantage over “homogeneous” tumors after docetaxel treatment (Fig. 1N, Fig. S4D-E), and RS_α_ and RF_α_ >1 (Fig. 1O-Q). Our data indicated that miR-125b^m^ cells enabled their Control^m^ partners to better survive chemotherapy, at the expense of their own fitness. We observed the same trend of both RS_α_ and RF_α_ >1 when miR-125b-expressing plasmid was used instead of miR-125b mimics (Fig. S4F-G), indicating that the phenomenon observed is agnostic to the methods of expression of miR-125b. We also demonstrated that docetaxel induced a reversible increase in *hsa-miR-125b-1* promoter activity in MDA-MB-231^miR-125bprom-EGFP^ cells (Fig. S2E), suggesting that miR-125b^m^ cells led to transient drug tolerance rather than permanent resistance in Control^m^ cells.

Overall, we observed the nature of the interactions between these two types of cells to be reminiscent of altruism as defined in social evolution theory [13, 30] (Fig. 1R), with miR-125b^m^ cells being altruistic (Supplementary Note 1). This conclusion is further supported by observation of growth of purified Mi/EGFP^High^ and Mi/EGFP^Low^ MDA-MB-231^miR-125bprom-EGFP^ in isolation or in-co-culture, a gold standard test for presence of cooperator versus cheater [42] and protective communal interaction [22]. As expected of the miR-125b-mediated fitness disadvantage, Mi/EGFP^High^ cells showed significantly lower growth rate as compared to Mi/EGFP^Low^ cells in the absence of drug treatment. Notably, a mixed population with a mere 10% Mi/EGFP^High^ survived significantly better than the expected viability with docetaxel treatment (Fig. 1S), indicating that the minority Mi/EGFP^High^ conferred a communal protective effect.

Taken together, these results support an altruistic role of miR-125b^High^ breast cancer cells: a minority of these cells confer a chemoprotective effect to the whole cell population, at a significant cost to self.

### Cell cycle inhibition underlies fitness disadvantage suffered by the altruists

To further characterize this altruistic phenotype, we investigated the nature and signaling mechanism underlying both the fitness benefits and cost, which are the two defining features of altruism.

Communal chemoprotection afforded by the altruistic miR-125b^High^ cancer cells comes at a fitness disadvantage to self (RF_α_ >1; Fig. 1K-M,P). We therefore set out to understand the mechanism of this self-sacrifice. In multiple cancers, miR-125b has been implicated in the inhibition of cell cycle progression [43], leading to growth inhibition and fitness reduction. miR-125b is known to directly inhibit *CDK2* [44] and *E2F3* [45], both of which are regulators of progression though G1-S phase, in non-breast cells. Using pull-down of biotinylated microRNA, we further identified *CCNA2* as another potential inhibitory target of miR-125b (Table S3). These cell cycle regulators are known to regulate G1-S transition (Fig. 2A) and were selected for further investigation as potential regulators of miR-125b-mediated fitness disadvantage. We first showed that heightened miR-125b expression indeed led to G1-S arrest as previously demonstrated, though the magnitude of the arrest differs between the two cell lines, likely due to e.g. differences in PTEN expression and consequently, level of activation of the  PI3K/AKT signaling pathway [46], which is known to affect G1-S transition [47] (Fig. 2B). Conversely, we also showed that inhibiting miR-125b using LNA inhibitor resulted in reduced G1-S arrest in MDA-MB-231 cells with endogenously high miR-125b expression (Fig. 2B). We next showed that heightened miR-125b expression via mimic transfection led to downregulation of protein expression of E2F3, CDK2 and CCNA2, and a known miR-125b target Bak1 [35], as well as reduced phosphorylation of retinoblastoma (Rb) (a protein whose phosphorylation is required for G1-S phase transition to proceed), and that this down-regulation was exacerbated with exposure to docetaxel (Fig. S5A). We likewise observed downregulation of *E2F3*, *CDK2, CCNA2* and *BAK1* using miR-125b-overexpressing plasmid in MCF7 cells (Fig. S5B), indicating that the inhibitory effect is not specific to the methods of miR-125b expression used. Meanwhile, inhibiting miR-125b using LNA inhibitor in the miR-125b-high MDA-MB-231 and MDA-MB-415 breast cancer cells resulted in increased E2F3, CDK2, CCNA2 and Bak1 protein expression and increased phosphorylation of Rb, which is indicative of increased G1-S progression with miR-125b inhibition (Fig. S5C).

We further showed that the expression of *E2F3*, *CDK2* and *CCNA2* transcripts are negatively correlated with expression of miR-125b but not that of the homologous miR-125a in breast cancer patients (Fig. S5D), supporting an inhibitory relationship between miR-125b and these cell cycle regulators. We also observed this inverse correlation using the MDA-MB-231^miR-125bprom-EGFP^ and MDA-MB-415^miR-125bprom-EGFP^ cells, whereby lower E2F3, CDK2 and CCNA2 protein expression was observed for purified Mi/EGFP^High^ cell fraction as compared to the Mi/EGFP^Low^ fraction and vice versa (Fig. S5E). We demonstrated, using RT-qPCR following microRNA pull-down assay, of significant enrichment of *E2F3*, *CDK2* and *CCNA2* transcripts with biotinylated miR-125b mimics transfected into MCF7 cells (Fig. 2C). We further confirmed the interaction using luciferase assay, where significant decreases in luciferase activities were detected when HEK293FT cells were co-transfected with miR-125b mimics and 3’UTR-containing reporter plasmid of *E2F3*, *CDK2* or *CCNA2* genes, and mutation to the putative microRNA binding sites on the respective 3’ UTRs rescued the decreases in luciferase activities (Fig. 2D, Fig. S5F).

Collectively, our data indicate the repression of multiple G1-S regulators of cell cycle by miR-125b as the mechanism underlying fitness reduction in the altruistic cancer cells.

### Non-cell-autonomous mechanism underlies fitness benefits conferred by the altruists

We observed near maximal benefits at only 30% miR-125b^m^ cells in docetaxel-treated MCF-7 population (Fig. S6A), with the highest rate of increase in benefits occurring when only 1-10% of the population was miR-125b^High^/altruistic (Fig. S6B). As aforementioned, we observed that docetaxel-exposed population survived significantly better than expected, when only 10% Mi/EGFP^High^ MDA-MB-231^miR-125bprom-EGFP^ cells were mixed with 90% Mi/EGFP^Low^ cells (Fig. 1S). Our data thus suggest that population-wide benefits may be attained when a small fraction of cells pay a cost to produce a collective benefit.

We postulated that this benefit may be rendered non-cell-autonomously, via sharing of extracellular trophic factor(s), as with the case of indole-secreting *E. coli* altruists [22]. RNA-seq analysis of Mi/EGFP^High^ versus Mi/EGFP^Low^ MDA-MB-231^miR-125bprom-EGFP^ cells revealed cytokine/chemokine production as a significantly enriched biological process in the former, thus implicating chemokines as such factor(s) (Fig. S6C-E). By comparing conditioned media from miR-125b^m^ versus Control^m^ MCF-7 cells using antibody array, we observed increased amount of chemokine CCL28 in the conditioned medium of the altruistic subpopulation (Fig. 2G). Using proteomics, we further identified IGFBP2, a component of the insulin signaling pathway, as being up-regulated in the conditioned media from miR-125b^m^ cells (Fig. 2H, Table S4). These results were further validated, using western immunoblotting, in breast cancer cell lines with perturbed expression of miR-125b (via transfection with mimics, overexpressing plasmid or LNA inhibitor) or are heterogeneous in miR-125b expression, whereby increased protein expressions of both IGFBP2 and CCL28 was observed in the conditioned media from cells with high miR-125b expression or Mi/EGFP^High^ subpopulations, and the protein expression reduced when miR-125b was inhibited or in Mi/EGFP^Low^ subpopulations (Fig. 2I, Fig. S6F). These experiments identified CCL28 and IGFBP2 as potential mediators of altruistic fitness benefits, with their secretions being increased with heightened miR-125b expression.

We further demonstrated that plasma levels of CCL28 and IGFBP2 proteins were significantly higher in breast cancer patients during taxane-based chemotherapy than post-chemotherapy (Fig. S6G, Table S5). High expression of both genes in neoadjuvant chemotherapy-treated breast tumor biopsies correlates with reduced OS and distance metastasis-free survival (DMFS) (Fig. S3E-I), supporting their role in chemoprotection. We therefore postulated that blocking these diffusible trophic factors would sensitize the resultant population/tumor to chemotherapy. Indeed, antibodies against these proteins markedly augmented docetaxel’s cytotoxic effect in breast cancer cell lines with endogenously high and heterogeneous miR-125b expression (MDA-MB-231, MDA-MB-415, HS578T; Fig. 2J, Fig. S6H, Fig. S2F-G) and in murine xenografts (Fig. 2K).

We further identified CCR10 as a possible receptor for CCL28 in miR-125^Low^ recipient cells, as antibodies against CCR10 significantly augmented docetaxel’s cytotoxicity (Fig. S6I). CCR10 is expressed at comparable levels in both miR-125^high^ and miR-125^Low^ cells (Fig. S6J, Supplementary Note 1) – it was previously shown in a “secrete-and-sense” model that at high cell density, it is heightened secretion rate, rather than a reduced receptor expression, of the secreting cells that promotes neighborly communication over self-communication [48]. This may explain why CCR10 expression was not down-regulated in miR-125^high^ altruists to minimize self-communication and self-benefit. We have yet to identify the receptor(s) for binding secreted IGFBP2, though the latter has been postulated to be able to directly translocate from extracellular milieu into cells [49].

Overall, our results implicate the trophic factors CCL28 and IGFBP2 in mediating the fitness benefits conferred by the miR-125b^High^ altruistic cancer cells.

### miR-125b dichotomizes via NF-κB signaling into altruistic fitness benefits and disadvantage

The secretion of IGFBP2 and CCL28 by miR-125b^High^ altruists to induce tumor-wide chemorefractoriness is an oncogenic process, while cell cycle inhibition by high miR-125b expression in the altruists is a tumor suppressive event. The activation of both oncogenic and tumor suppressing processes in the same cancer cells is hitherto a puzzle [50]. MicroRNAs are known to be able to trigger both processes concurrently due to the large number of genes modulated by each microRNA [50]. Through RNA sequencing of miR-125b^High^ and miR-125b^Low^ breast cancer cell lines, we further observed increased expression of multiple known oncogenes and tumor suppressors, as well as genes markedly up- and down-regulated in breast cancer tissues versus normal adjacent tissues, in the miR-125b^High^ altruistic subpopulations when compared to the miR-125b^Low^ counterparts (Fig. S7A-H), implying that co-activation of seemingly competing cancer processes might be pivotal for manifestation of miR-125b-driven cancer cell altruism. On this ground, we wondered how heightened miR-125b expression may lead to concurrent activation of both processes. We began the investigation by deciphering the key signaling pathway(s) operating downstream of miR-125b in the altruists. Since miR-125b is known to activate Wnt and NF-κB pathways [39, 51], we further investigated if these signaling pathway(s), parts thereof, may regulate fitness disadvantage and benefits.

Through preliminary screening using inhibitor/agonist libraries (Fig. S5G-H) and subsequent validation using siRNA-mediated gene knock-down (Fig. 2E, Fig. S5I-J), we identified IKKβ, a component of the NF-κB signaling, which, when knocked-down in miR-125b^m^ altruists, resulted in significant rescue of miR-125b-driven fitness disadvantage using cell lines (Fig. 2E, Fig. S5I-J) and tumor xenograft models (Fig. S5K-L). We further showed that knockdown of *IKBKB* (coding for IKKβ) but not *p65* rescued miR-125b-inducd reduction in E2F3, CDK2 and CCNA2 protein expression, and Rb phosphorylation, which is indicative of increased G1-S phase progression (Fig. 2F, Fig. S5M). Notably, IKKβ knockdown did not rescue miR-125b-induced reduction in Bak1 expression (Fig. 2F). This, together with our demonstration of a negative correlation between gene expressions of *E2F3*, *CDK2* and *CCNA2* with *IKBKB* expression in breast cancer patients (Fig. S5D), implicates IKKβ in regulating cell cycle progression in cooperation with miR-125b. In contrast, knocking-down of both *IKBKB* and *p65*, a more downstream component of NF-κB signaling pathway, attenuated miR-125b-induced NF-κB activation (Fig. S5N-O). Collectively, these results suggest that the fitness disadvantage suffered by altruistic miR-125b^m^ cancer cells is not mediated through p65-mediated NF-κB signaling but rather, via an NF-κB-independent function of IKKβ. The latter is known to have specific pro-tumorigenic functions that are not dependent of its involvement in NF-κB signaling [52].

Meanwhile, we showed that knockdown of *p65* negated miR-125b-induced increased in secretion of both IGFBP2 and CCL28 into the conditioned media of MCF7 and MDA-MB-468, while knockdown of *IKBKB* negated the increased in secretion of IGFBP2 but not CCL28 (Fig. 2L). Functionally, knock-down of both *IKBKB* and *p65* led to reduction in the RS_α_ with docetaxel exposure (Fig. 2M). Together, these results indicate that the fitness benefits induced by miR-125b expression is mediated through p65-mediated NF-κB signaling, though the requirement for IKKβ differs between the two secreted factors.

Our results point to the involvement of differential NF-κB signaling in mediating the dichotomization of upstream miR-125b signaling, resulting in concurrent manifestation of the oncogenic and tumor suppressive processes that underlie altruistic fitness benefits and disadvantage respectively (Fig. 2N-O).

### Modeling based on evolutionary game theory predicts persistence of altruistic subpopulation

Our observations thus far reveal a system in which a few cells induce a community benefit by secreting trophic factors (IGFBP2 and CCL28) that act as public goods for the whole tumor, at a significant cost to the secreting cells themselves, due to arrest of their own cell cycle - an example of altruism among cancer cells. This raises a conceptual issue: how can a subpopulation with a significant fitness cost be maintained in a population of cells with apparently higher fitness? In a previous mouse model of clonal heterogeneity, minor cell subpopulation driving tumor growth non-cell-autonomously was shown to be driven to extinction by faster growing competitor subclones [10], a situation akin to the concept known as “tragedy of the commons” [53] - free-riding on the contributions of other group members enables selfish individuals to benefit at the expense of altruistic individuals, leading to the spread of free-riding defectors and the collapse of cooperation.

Given the fitness cost (Fig. 1K-M,P) associated with high miR-125b expression, and the competition from defector cells, the standard paradigm of clonal selection predicts that the altruistic trait cannot persist in population. When fitness depends on diffusible factors, however, selection is frequency-dependent (the fitness of the defectors decreases as their frequency increases), and theory predicts (Supplementary Note 2) that, because the effect of the diffusible cooperative factors is concave (saturating), the population will evolve to a stable polymorphic equilibrium in which miR-125b^High^ altruistic cells coexist at low frequencies with non-producer cells, if a population is well-mixed. Theory also predicts (Fig. 3A, Supplementary Note 3), however, that in spatially structured populations such as cancer cells *in vitro* and *in vivo*, the type of saturating benefits we observed would lead to the extinction of the altruistic cancer cells, unless the altruists can be readily regenerated from defectors in a type of “best response” dynamics” [54] that generally occurs in interactions among rational payoff-maximizing agents (such as humans) [55]. We hypothesized that such dynamics is possible in cancer cells only if it is driven by an epigenetic mechanism (Fig. 3A), which would enable rapid interconversion of cells from one type to the other.

### Altruistic cancer cells regenerate via epigenetic mechanism

Epigenetic mechanisms are already known to regulate behavioral plasticity in eusocial insects with altruistic worker castes, such as carpenter ants [56] and honeybees [57]. We further demonstrated that Mi/EGFP^High^ altruists reappeared when isolated Mi/EGFP^Low^ cancer cells were allowed to grow (and vice versa) for both MDA-MB-231^miR-125bprom-EGFP^ and MDA-MB-415^miR-125bprom-EGFP^ cells (Fig. 3B) and murine xenograft (Fig. S8A). We showed that sorted Mi/EGFP^High^ and Mi/EGFP^Low^ fractions returned to pre-sort Mi/EGFP^High^–minority:Mi/EGFP^Low^–majority equilibrium, with levels of extracellular CCL28 and IGFBP2 proteins (Fig. S8B), and transcriptome profiles (Fig. S8C) of the two sorted fractions converging over time. Regenerated populations retained the ability to increase Mi/EGFP level upon docetaxel exposure (Fig. S8D).

The dynamic nature of the regeneration of Mi/EGFP^High^ cells from Mi/EGFP^Low^ cells that we observed, coupled with our mathematical model, points to an epigenetic mechanism in maintaining a stable equilibrium of miR-125b^High^ altruists and miR-125b^Low^ non-altruists in the population. Treatment of MDA-MB-231^miR-125bprom-EGFP^ cells with pharmacological inhibitors and siRNA implicated the histone acetyltransferase P300/CBP-associated factor (PCAF) and the transcription factor KLF2 in the regulation of *hsa-miR-125b-1* promoter via acetylation during regeneration and taxane-induced increased expression (Fig. 3C, Fig. S8E-F). The promoter region is shown to be enriched in H3K27Ac histone mark, as determined via ChIP-seq assays in mammary epithelial cells and non-breast cell lines from ENCODE (Fig. S9A). Using chromatin immunoprecipitation (ChIP), we observed acetylation of histone H3 and H4 on the promoter for Mi/EGFP^Low^ cells, but not for Mi/EGFP^High^ cells (Fig. S9B), indicating that histone acetylation may underlie epigenetic rewiring of the miR-125^Low^ cells to regenerate into the miR-125^High^ fraction.

We subsequently demonstrated that knockdown of *KLF2* and *PCAF* retarded the regeneration of Mi/EGFP^High^ cells from isolated Mi/EGFP^Low^ cells (Fig. 3D, Fig. S9C). We identified two KLF2 consensus binding sites, KBS-1 and KBS-2, located in parts of the promoter known to be enriched in H3K27Ac histone mark (Fig. S8G). ChIP performed on *KLF2* knocked-down cells confirmed KLF2 binding to promoter regions harboring these consensus sites and demonstrated reduced H3/H4 acetylation on KBS-1 and reduced PCAF binding on KBS-2 with KLF2 knock-down (Fig. S9D). We designed CRISPR interference (CRISPRi) to block KLF2 binding to either sites, and showed that one (for KBS-1; Fig. 3E, Fig. S9F) decreased overall Mi/EGFP level (Fig. S8H, Fig. 3G), retarded regeneration of Mi/EGFP^High^ cells from isolated Mi/EGFP^Low^ cells (Fig. 3F), reduced binding of KLF2 to the promoter (Fig. S9E), diminished extracellular CCL28 and IGFBP2 levels (Fig. 3J), and blunted the chemotolerance of three different breast cancer cell lines (Fig. 3H) and xenograft tumor (Fig. 3I) against docetaxel. Interestingly, an insect homolog of KLF2 (known as Kr-h1) has been implicated in caste-specific behavior of altruistic workers and selfish queen in *Harpegnathos saltator* ants [58], which, together with our findings, suggest that members *of* the Krüppel-like transcription factor family may play a role in regulation of plasticity of social behavior. Overall, our results not only validate the chemoprotective effect of the miR-125^High^ subpopulation, but also indicate that the KLF2-mediated epigenetic mechanism underlies the regeneration and persistence of a subpopulation of miR-125^High^ altruists within the tumor.

### Lateral inhibition maintains a stable organization of altruists and non-altruists

**Fig. 4:**
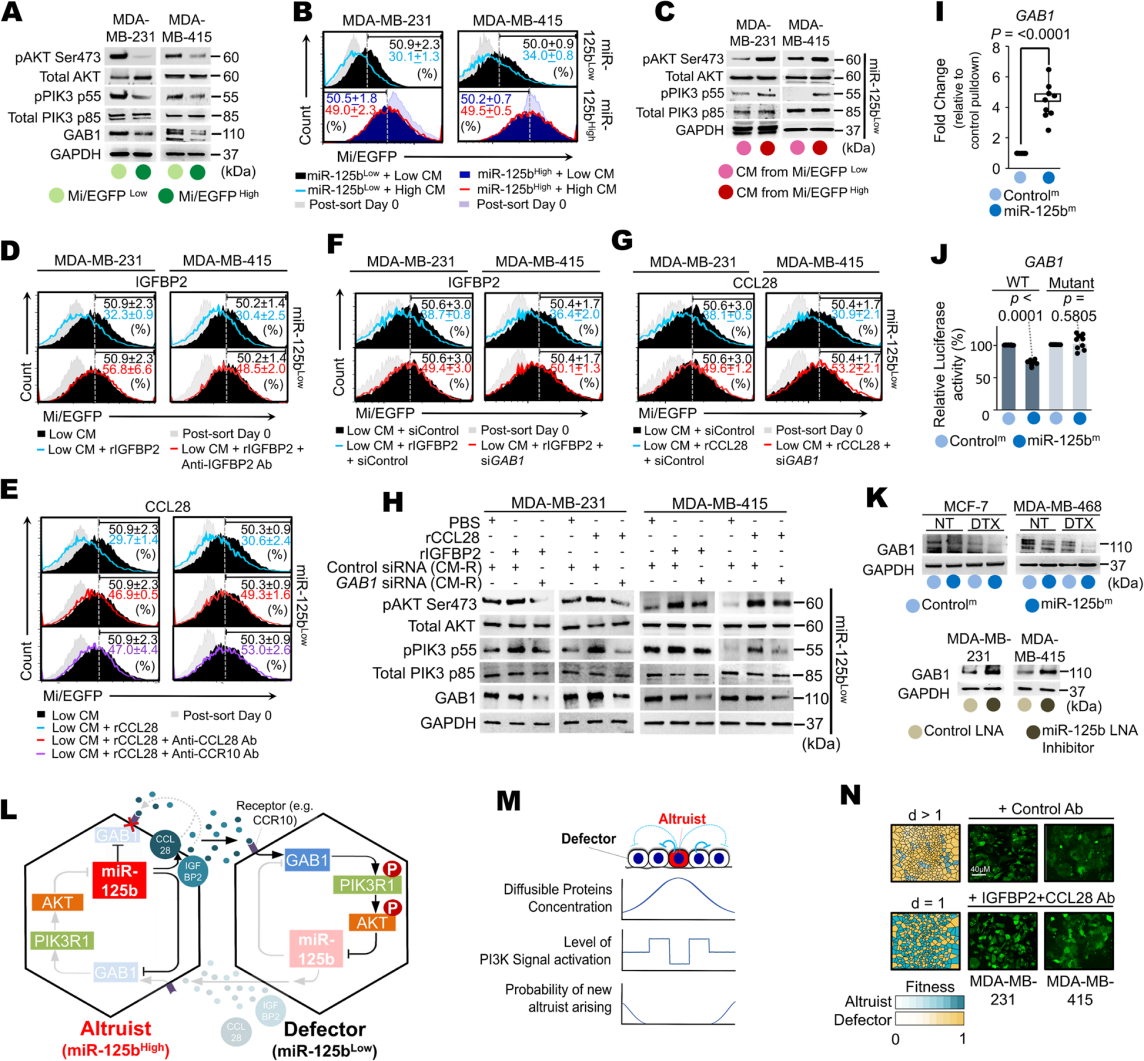
Lateral inhibition maintains a sparse spatial organization of altruists. **A** Immunoblotting to detect indicated proteins extracted from Mi/EGFP^High^ and Mi/EGFP^Low^ fractions from indicated cancer cell lines. **B,C** Mi/EGFP^Low^ and Mi/EGFP^High^ cells from indicated cancer cell lines were exposed to conditioned media harvested from separate batches of Mi/EGFP^Low^ and Mi/EGFP^High^ cells. After four days, the formers’ fluorescence levels were analyzed by FACS (B) and extracted protein of exposed Mi/EGFP^Low^ cell studied by immunoblotting (C). **D, E** Mi/EGFP^Low^ cells from indicated cell lines were treated with indicated recombinant proteins in combination with neutralizing antibodies and the fluorescence level analyzed by FACS after four days. **F-H** Mi/EGFP^Low^ cell fractions from indicated cancer cell lines, transfected with control or *GAB1* siRNA, were exposed to recombinant IGFBP2 or CCL28. After four days, their fluorescence levels were analyzed by FACS (F, IGFBP2; G, CCL28) and their extracted protein by immunoblotting (H). CM-R: Mi/EGFP^Low^ recipient cells exposed to recombinant protein. **I** Box plots of fold change in enrichment of *GAB1* mRNA pulled-down using biotinylated miR-125b mimics. **J** Relative luciferase activities of HEK293T cells following transfection of wild-type or mutant reporter construct for *GAB1* and indicated mimics. WT: wild type. **K** Immunoblotting to detect expression of indicated proteins extracted from miR-125b^m^ or Control^m^-transfected MCF7 or MDA-MB-468 cells with or without docetaxel treatment (Upper), or miR-125b LNA-inhibitor- or control-LNA-transfected MDA-MB-231 or MDA-MB-415 cells (Lower). **L,M** Schema of lateral inhibition model mediated by diffusible IGFBP2 and CCL28 secreted by miR-125b^High^ altruists. Upon exposure to IGFBP2 and CCL28, heightened PI3K-AKT signaling is induced in the recipient cells, resulting in reduction in miR-125b expression and adoption of the non-altruistic social fate (L). Hypothesized level of the diffusible proteins, extent of PI3K activation and probability of altruist arising as the distance from altruist increases is depicted, in association with the altruist’s ability to influence social fates beyond the immediate neighboring cells (M). **N** Simulation of lateral inhibition dynamics showing pattern generation when diffusion coefficient d = 1 (as in the case of Notch-Delta signaling) and d>1 (mediated by diffusible proteins such as IGFBP2 and CCL28) (Left column). Spatial patterns of Mi/EGFP^High^ and Mi/EGFP^Low^ cells in indicated cell lines, with or without IGFBP2 & CCL28 antibodies treatment, are shown (Center and Right columns). Experiments repeated two times, representative data are shown for (A-H, K, N). Mean percentage ± s.d. cells for technical triplicates of representative set are shown in same colour as corresponding histograms (B,D-G). Data are mean ± s.d. from three independent biological sets of triplicates (I, J). Statistical analysis was performed using two-tailed one sample *t*-test against 1 (I) or 100 (J). NT: no treatment; DTX: docetaxel treatment. Exact *P* values are shown

We observed that the frequency of altruistic cancer cells with high Mi/EGFP fluorescence remained consistently low with repeated purification and regeneration of Mi/EGFP^Low^ cell fraction of MDA-MB-231^miR-125bprom-EGFP^ (Fig. S10A). This begs the question of how such a biased balance between the altruists and defectors can be modulated and consistently maintained in the cancer cell population. We began this part of the study by determining if differential signaling occurs between the altruists and non-altruists. MiR-125b has been implicated in PI3K-AKT signaling [59], and we observed activation/phosphorylation of PI3K and AKT with low Mi/EGFP fluorescence, and vice versa (Fig. 4A), indicating an inverse correlative relationship between PI3K activation and miR-125b expression. We also demonstrated that purified Mi/EGFP^Low^ fraction showed greater reduction in cell viability than Mi/EGFP^High^ fraction when treated with PI3K/AKT inhibitors Ly294002 and AKT IV (Fig. S10B), validating the higher PI3K/AKT activation in miR-125b^Low^ subpopulation. We further showed that inhibiting PI3K or AKT activation using small molecule inhibitors resulted in increase in Mi/EGFP fluorescence in unsorted MDA-MB-231^miR-125bprom-EGFP^ and MDA-MB-415^miR-125bprom-EGFP^ (Fig. S10C), indicating that PI3K/AKT activation negatively modulates miR-125 expression.

Exogenous factors are known to activate PI3K/AKT signaling [60] and we have previously shown that altruistic miR-125b^High^ breast cancer cells secrete trophic factors such as CCL28 and IGFBP2 (Fig. 2G -I) – we thus hypothesized that exogenous CCL28 or IGFB2 may induce PI3K-AKT activation, thereby reducing miR-125b expression. We first demonstrated that exposure to conditioned medium (CM) collected from Mi/EGFP^High^ fraction sorted from MDA-MB-231^miR-125bprom-EGFP^ and MDA-MB-415^miR-125bprom-EGFP^ cells retarded the regeneration of Mi/EGFP^Low^ cells but not Mi/EGFP^High^ cells (Fig. 4B) when compared to those exposed to CM from Mi/EGFP^Low^ cells. We further showed increased phosphorylation of PI3K and AKT in Mi/EGFP^Low^ cells exposed to CM from Mi/EGFP^High^ donors as compared to that from the Mi/EGFP^Low^ donors (Fig. 4C). Inhibiting miR-125b in the Mi/EGFP^High^ CM donors using LNA inhibitors negated the phosphorylation of PI3K and AKT in the Mi/EGFP^Low^ recipient cells (Fig. S10D), indicating that miR-125b expression in Mi/EGFP^High^ donors is required to mediate the ability of the Mi/EGFP^High^ CM in activating PI3K/AKT in the recipient cells. Adding neutralizing antibodies against IGFBP2, CCL28 or CCR10 (receptor of CCL28) on top of CM from Mi/EGFP^High^ rescued the retardation in Mi/EGFP fluorescence (Fig. S10E-F), thus implicating IGFBP2 and CCL28 as the exogenous factors within the CM from Mi/EGFP^High^ that activated PI3K/AKT signaling in the Mi/EGFP^Low^ recipient cells. We next showed that adding recombinant IGFBP2 or CCL28 retarded the regeneration of Mi/EGFP^Low^ cells (Fig. 4D-E), while addition of neutralizing antibodies (Fig. 4D-E) or PI3K/AKT inhibitors (Fig. S10G) rescued this retardation. Together, our data indicate that altruist-secreted IGFBP2 and CCL28 trigger that activation of PI3K-AKT signaling in the recipient cells, which in turn leads to downregulation of miR-125b expression. These findings, coupled to our demonstration that PI3K/AKT inhibitors in combination with docetaxel resulted in significantly reduced cell viability in freshly sorted Mi/EGFP^Low^ subpopulation than docetaxel alone (Fig. S10H), indicate that IGFBP2/CCL28-induced PI3K-AKT activation modulates cell viability and suggest that IGFBP2/CCL28-induced chemotolerance of the miR-125b^Low^ non-altruists may be mediated via heightened PI3K-AKT activation.

We wondered what might mediate the signal transduction from the exogenous factors to PI3K/AKT signaling within the cells, and focused on GAB1, a member of the receptor-associated docking adaptor protein family that recruits and promotes activation of PI3K through interaction with the p85 subunit [61]. We showed that knock-down of *GAB1* rescued the retardation in Mi/EGFP fluorescence induced by Mi/EGFP^High^ CM (Fig. S10I), recombinant IGFBP2 or CCL28 (Fig. 4F-G), as well as negated the phosphorylation of PI3K and AKT induced by the same exogenous factors (Fig. S10J, Fig. 4H). Our results thus indicate that GAB1 mediates IGFBP2/CCL28-induced activation of PI3K/AKT in the Mi/EGFP^Low^ recipient cells. We were also interested in how miR-125b may regulate the expression of GAB1, since we observed low GAB1 protein expression in Mi/EGFP^High^ cells and vice versa (Fig. 4A). Unexpectedly, we uncovered that *GAB1* mRNA as a significant target of miR-125b using biotinylated microRNA pulldown experiment (Table S3) and validated the interaction using RT-qPCR (Fig. 4I) and luciferase assay (Fig. 4J, Fig. S10K). We further showed that heightened miR-125b expression via mimic or plasmid transfection led to downregulation of *GAB1*, while inhibiting miR-125b using LNA inhibitor in the high miR-125b expressing MDA-MB-231 and MDA-MB-415 cells resulted in increased GAB1 protein expression (Fig. 4K, Fig. S5B). Our data thus showed that miR-125b modulates the activation of PI3K and AKT via direct inhibition of GAB1. Importantly, the reduction of GAB1 by high miR-125b expression in the altruists may serve to avert self-activation of PI3K/AKT signaling in the altruists, thus avoiding self-quenching of miR-125b expression and instability in manifestation of the altruistic phenotype in the population.

Our collective data points to a model of lateral inhibition involving altruist-secreted factors and the GAB1-PI3k-AKT-miR-125b regulatory circuit in the self-organization of binary social fate (Fig. 4L). Unlike the widely-studied Notch-Delta lateral inhibition mechanism that involves cell membrane-bound ligand-receptor juxtacrine communication [62], our lateral inhibition model involves diffusible secreted factors that can influence beyond the immediate neighboring cells of the altruists (Fig. 4M). Our modeling showed that lateral inhibition mediated by diffusible proteins leads to a patterning of sparse altruistic cells amidst a majority of non-altruistic defectors, which is unlike the “salt-and-pepper” patterning of the Notch-Delta lateral inhibition [63] (Fig. 4N). Treatment of MDA-MB-231^miR-125bprom-EGFP^ and MDA-MB-415^miR-125bprom-EGFP^ cells with a mixture of antibodies against IGFBP2 and CCL28 resulted in the “salt-and-pepper” patterning in reminiscence of that produced by Notch-Delta signaling (Fig. 4N), likely due to the actions of the secreted factors being limited to tight cell-cell interface not accessible to the antibodies. Our results herein thus support a “secrete-and-sense” mechanism of lateral inhibition involving altruist-secreted diffusible factors in modulating and maintaining a sparse but stable spatial organization of different social fates within the cancer cell population.

## Discussion

**Fig. 5:**
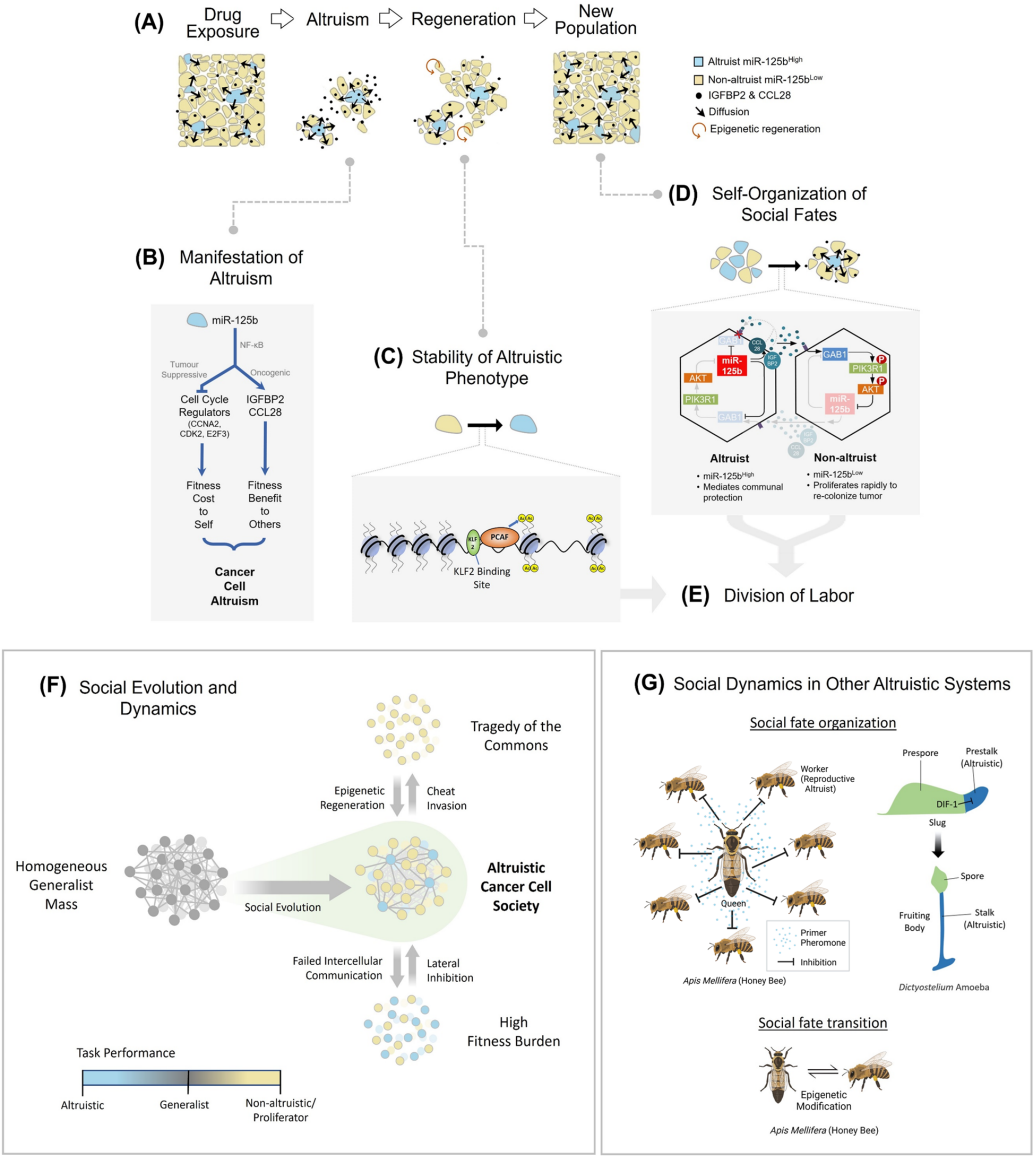
Breast tumor as a dynamic social system manifesting altruistic cooperation. **A** In altruistic cooperation, a small subpopulation of altruistic cells (blue) confers communal protection against taxane exposure by secreting trophic factors (IGFBP2 and CCL28) that activate PI3K/AKT signaling and thus leading to heightened chemotolerance in neighboring cells (yellow). During post-treatment expansion phase, the altruistic subpopulation, saddled with a fitness disadvantage due to miR-125b-mediated cell cycle impediment, risks becoming extinct due to competition from the faster growing neighboring non-altruists. **B** Conferment of survival benefits to others (an oncogenic event) and incurring of fitness cost to self (a tumor suppressive event), both of which are defining attributes of altruism, are found to be commonly mediated by heightened miR-125b expression, via differential NF-κB signaling, in the altruistic cancer cells. **C** The altruistic subpopulation persists, due to phenotypic conversion from the neighboring non-altruists via a KLF2/PCAF-mediated epigenetic mechanism acting on the promoter of the *hsa-miR-125b-1* gene. **D** The tumor cell population actively self-organizes via a lateral inhibition mechanism mediated by IGFBP2/CCL28-induced GAB1-PI3K-AKT-miR-125b signaling circuit. This limits the altruistic subpopulation to a minority presence and a sparse spatial arrangement. A closer look at the lateral inhibition model (below) shows inhibition of GAB1 by high miR-125b expression in the altruist, which prevents self-activation of PI3K/AKT by the altruists-secreted IGFBP2 and CCL28, thus averting self-benefiting and instability of the altruistic phenotype. **E** The lateral inhibition mechanism, coupled with epigenetic regenerability of the altruists, permits stable co-existence of functionally distinct subpopulations: an altruistic miR-125b^High^ minority confers costly communal protection during chemotherapeutic crisis while the miR-125b^Low^ majority undergoes aggressive proliferation post-crisis to re-colonize the tumor. Cooperation between these different phenotypes suggests the existence of division of labor, a hallmark of complex biological societies, within the breast tumor. **F** One possible explanation of the origin of altruistic tumor society is evolution from a homogeneous population of generalist cancer cells. The composition of the resulting altruistic society of cancer cells can theoretically be perturbed with varying ecological consequences. Without epigenetic regeneration of altruists, non-altruists or “cheats” would dominate and deplete existing resources, leading to a situation called the “tragedy of the commons” [53]. Conversely, without lateral inhibition, altruists would dominate the population, hence inflicting a fitness burden on the tumor. Breast tumor may thus constitute a potential model to study how tumor-specific ecological factors can affect evolution and manifestation of altruistic cooperation. **G** Examples of altruistic social systems and how the social dynamics is regulated. Above: In honeybee (*Apis Mellifera*), the queen bee secretes primer pheromone such as CHCs and other glandular compounds that suppress worker ovarian development, thus maintaining the workers as reproductive altruists [64]. In *Dictyostelium* amoeba, the pre-spores secrete differentiation-inducing factor-1 (DIF-1) to prevent the altruistic pre-stalk cells from developing into spores, thus maintaining a 80:20 spore-to-stalk cell ratio in the fruiting body that is formed eventually [65]. Such secretion-mediated regulation of cell fate is similarly observed in the altruistic breast cancer cells. Below: Epigenetic regulation is known to underlie behavioral plasticity in *Apis Mellifera* [57], and we likewise observed how epigenetic mechanism regulates social fate plasticity in breast cancer cells

Our discovery of costly but regenerable altruistic behavior and self-organization of social fates amongst breast cancer cells supports the notion of the breast tumor as a self-perpetuating and self-organizing social system that might underpin therapy refractoriness (Fig. 5A). Being a well-established experimental system for cancer study, our breast cancer cell models have permitted insights into mechanisms underlying altruistic manifestation. First, we found that concurrent activation of oncogenic and tumor suppressive effects underlie altruistic fitness benefits and cost respectively, and this was orchestrated by a single regulator RNA, miR-125b, via the involvement of different parts of the NF-κB signaling pathway (Fig. 5B). This raises the possibility that seemingly paradoxical co-activation oncogenic and tumor suppressive pathways may be required for manifestation of complex traits, such as altruism, in cancer cells. Second, we found that altruistic cells can be regenerated from the non-altruistic fate via a KLF2-mediated epigenetic mechanism (Fig. 5C), hence circumventing the Darwinian paradox that altruists should be driven to extinction by natural selection. Third, we demonstrated that the direct inhibition of GAB1 expression by miR-125b prevented self-benefiting of the altruists by its own secreted products (Fig. 5D), hence qualifying the cooperative behavior that we observed as being truly altruistic. Fourth, GAB1 was also a critical link in the PI3K-mediated “secrete-and-sense” circuit which, together with the diffusible nature of the secreted products, underlie a lateral inhibition mechanism that led to a characteristic sparse spatial patterning of altruists in the population (Fig. 5D). Our results demonstrate that this mechanism can couple the conferment of altruistic fitness benefits with social fate determination, through the common involvement of IGFBP2 and CCL28 as tools for intercellular communication. Taken together, our findings show a cohesive mechanistic system that underlies a complex social behavior like altruism.

The lateral inhibition mechanism, coupled with the regenerative capability of the altruistic subpopulation, underlies the stable co-existence of two functionally distinct phenotypes in a characteristic ratio: a minority one (altruist) that confers communal protection during chemotherapeutic crisis, and the majority one (non-altruist) that enables rapid proliferation post-crisis, as evident by RF_α_ >1 (Fig. 1K-M, P), to re-colonize the tumor. Such phenotype co-existence indicates the possibility of division of labor (Fig. 5E), which has long been appreciated as central to the evolution of complex biological societies [66]. Division of labor tends to occur in clonal groups and is fostered by cooperative (and not exploitative) relationship between different phenotypic groups [66] – prerequisites that were met in our study demonstrating altruistic interactions amongst clonal breast cancer cells of different miR-125b expression levels. Collectively, our findings point to an equilibrium in the altruistic social structure in a tumor, to which hypothetical perturbations would lead to ecological consequences (Fig. 5F). For example, without the regeneration of altruists, the resultant population will inevitably consist of only non-altruists, or “cheats” that free-ride on available resources and would be vulnerable to therapeutic challenges - in an ecological scenario known as “tragedy of the commons” [53]. On the other hand, without lateral inhibition, e.g. due to neutralization of the altruists-secreted proteins using antibodies (Fig. 4N), the appearance of too many altruistic cells would burden the tumor with a fitness penalty (Fig. 5F). The mechanisms regulating the social dynamics of breast cancer cells are in reminiscence of those observed in social organisms such as honeybees and social amoeba (Fig. 5G), suggesting that breast cancer cells could serve as a valuable model to study social processes in more complex organisms.

By elucidating the mechanisms and dynamics of altruism within well-characterized cancer cell models, we can gain insights that have broad implications across fields such as sociobiology and medicine. Our multidisciplinary framework for reconciling seemingly counterintuitive mechanisms with the altruistic phenotype can be extended to characterizing altruism in other social organisms. Furthermore, identifying and understanding the altruistic interactions among cancer cells can lead to more holistic and accurate models of tumor evolution that account for both cooperative and competitive interactions. These models can in turn contribute to a broader understanding of cooperation and competition in driving the evolution of complex social systems, including eusocial insects and microbial communities. Ultimately, our work underscores the potential of cancer cells as a model system for exploring fundamental questions in evolutionary biology and for identifying vulnerabilities in complex social interactions that can be exploited for biological control or therapeutic gain.

## Conclusion

Overall, our study revealed the mechanisms underlying the manifestation, persistence and spatial patterning of unique cooperative interactions such as altruism within the cancer cell population (Figure 5). Recognizing the phenomenon of altruism among cancer cells expands our understanding of the repertoire of tumor capabilities and their abilities to escape modern therapeutic strategies. Insights into how altruistic interactions within a tumor shape drug refractoriness may also open up a fresh paradigm of therapeutic intervention. Lastly, our report may also bear implications to the study of altruism in other social organisms. Cancer cells, which are highly amenable to experimental evolution, molecular perturbation, and comprehensive single-cell profiling, may emerge as an invaluable model system for evolutionary and mechanistic studies of social evolution.

### Supplementary Information

**Additional file 1**:** Supplementary Note 1.** Definition of altruism. **Supplementary Note 2.** Modeling based on evolutionary game theory predicts persistence of altruistic subpopulation. **Supplementary Note 3.** Dynamics in spatially structured populations hints at epigenetic mechanism in mediating persistence of altruists. **Fig. R1.** Social matrix of interaction. From Hamilton (1964) and West et al. (2006). **Fig. R2.** Survival of the fittest and the tragedy of the commons within tumors. **Fig. R3.** Frequency-dependent selection. **Fig. R4.** Dynamics of altruism. **Fig. R5.** Estimating the parameters. **Fig. R6.** The observed saturating effect leads to a stable minority of altruistic cancer cells. **Fig. R7.** Epigenetic determination enables stable altruism. **Fig. R8.** Increasing therapy leads to more altruistic cooperation. **Fig. R9.** Why epigenetic-regulated altruism can persist. **Fig. S1.** microRNA expression in CTCs from breast cancer patients undergoing taxane treatment. **Fig. S2.** Heterogeneous expression of miR-125b in patient tumor samples and breast cancer cell lines. **Fig. S3.** Stratification of breast cancer patients based on expression levels of miR-125b, IGFBP2 and CCL28. **Fig. S4.** Relative survival analysis of miR-125b-associated behavior of breast cancer cell lines and xenografted tumors. **Fig. S5.** Mechanism of miR-125b-driven fitness disadvantage of cancer cell altruism. **Fig. S6.** Mechanism of miR-125b-driven fitness benefit of cancer cell altruism. **Fig. S7.** Heightened co-expressions of oncogenes and tumor suppressors in miR-125b^High^ altruists. **Fig. S8.** MiR-125b^High^ altruists are regenerable via a KLF2-mediated acetylation. **Fig. S9.** ChIP reveals protein-chromatin interactions at two different KLF2 binding sites. **Fig. S10.** Lateral inhibition maintains a sparse spatial organization of altruists. **Table S1.** Clinical characteristics of breast cancer patients for miRNA profiling of CTCs (Supplementary Fig. 1). **Table S2.** Clinical characteristics of breast cancer patients for miR-125b *in-situ* hybridization (Fig. 1A) **Table S3.** Selected results of comparative analysis of RNA-Sequencing of biotinylated miR-125b mimics versus control mimic pulldown (Figs. 2 and 4). **Table S4.** Results of comparative proteomic analysis of conditioned media from miR-125b or control mimic-transfected MCF7 cells using iTRAQ (Fig. 2H). **Table S5.** Clinical characteristics of breast cancer patients for detection of IGFBP2 and CCL28 protein levels in plasma (Fig. S6G).

## Data Availability

All data are available in the main text or the supplementary materials. Single-cell RNA seq and ChIP-seq data are available under Gene Expression Omnibus (GEO) accession number GSE123358 and GSE123266 respectively. Source data for all figures are available from the corresponding authors upon reasonable request. pLU-Jarid1Bprom-eGFP-pBlast used for making the promoter miR-125b reporter cell lines was obtained from Wistar Institute, USA under a material transfer agreement with the National University of Singapore.

## Abbreviations

3’-UTR: 3’ Untranslated region
AKT: AKT serine/threonine-specific kinase
Bak1: BCL2 Antagonist/Killer 1
CCL28: C-C motif chemokine ligand 28
CCNA2: Cyclin A2
CCR10: C-C motif chemokine receptor 10
CDK2: Cyclin-dependent kinase 2
ChIP: Chromatin immunoprecipitation
CM: Conditioned medium
control^m^: Cells transfected with control microRNA mimics
*CRISPR*: Clustered regularly interspaced short palindromic repeats
CRISPRi: CRISPR interference
CTC: Circulating tumor cells
DAPI: 4’,6-Diamidino-2-Phenylindole
dCas9: Endonuclease deficient Cas9
DMFS: Distance metastasis-free survival
E2F3: E2F Transcription factor 3
ELISA: Enzyme-linked immunosorbent assay
EpCAM: Epithelial cellular adhesion molecule
FACS: Fluorescence-activated cell sorting
FFPE: Formalin-fixed paraffin-embedded
FOXO3: Forkhead Box O3
GAB1: GRB2 associated binding protein 1
GAPDH: Glyceraldehyde 3-phosphate dehydrogenase
GSK-3β: Glycogen synthase kinase-3 beta
H&E: Haematoxylin and eosin
H3K27Ac: Acetylation at the lysine residue at N-terminal position 27 of the histone H3 protein
HDAC3: Histone deacetylase 3
HRP: Horseradish peroxidase
IGFBP2: Insulin-like growth factor binding protein 2
IKKβ or IKBKB: Inhibitor of nuclear factor kappa B kinase subunit beta.
ISH: In-situ hybridization
iTRAQ: Isobaric tags for relative and absolute quantification
KBS-1: KLF2 consensus binding site 1
KBS-2: KLF2 consensus binding site 2
KLF2: Krüppel-like factor 2
LNA: Locked nucleic acid
Mi/EGFP: Emerald green fluorescent protein reporter driven by the *hsa-miR-125b-1* promoter sequence
miR-125b: MicroRNA-125b
miR-125b^m^: Cells transfected with miR-125b mimics
MiRNA: MicroRNA
MTS assay: 3-(4,5-Dimethylthiazol-2-yl)-5-(3-carboxymethoxyphenyl)-2-(4-sulfophenyl)-2H-tetrazolium assay
NF-κB: Nuclear factor kappa-light-chain-enhancer of activated B cells
OS: Overall survival
PCAF: Histone acetyltransferase P300/CBP-associated factor
PI3K: Phosphoinositide 3-kinase
Rb: Retinoblastoma
RF: Relative Fitness
RS: Relative Survival
RT-qPCR: Reverse transcription quantitative real-time PCR
SDS-PAGE: Sodium dodecyl-sulfate polyacrylamide gel electrophoresis
siRNA: Silencing RNA
SMO: Smoothened, frizzled class receptor
TIP60 (or KAT5): Lysine acetyltransferase 5
