# Supplementary material for: Dynamic altruistic cooperation within breast tumors
Source: Mol Cancer. 2023 Dec 14;22:206. doi: 10.1186/s12943-023-01896-7 (PMC10720132; doi:10.1186/s12943-023-01896-7)
Supplement: Supplementary file 1 — Additional file 1: Supplementary Note 1. Definition of altruism. Supplementary Note 2. Modeling based on evolutionary game theory predicts persistence of altruistic subpopulation. Supplementary Note 3. Dynamics in spatially structured populations hints at epigenetic mechanism in mediating persistence of altruists. Fig. R1. Social matrix of interaction. From Hamilton (1964) and West et al. (2006). Fig. R2. Survival of the fittest and the tragedy of the commons within tumors. Fig. R3. Frequency-dependent selection. Fig. R4. Dynamics of altruism. Fig. R5. Estimating the parameters. Fig. R6. The observed saturating effect leads to a stable minority of altruistic cancer cells. Fig. R7. Epigenetic determination enables stable altruism. Fig. R8. Increasing therapy leads to more altruistic cooperation. Fig. R9. Why epigenetic-regulated altruism can persist. Fig. S1. microRNA expression in CTCs from breast cancer patients undergoing taxane treatment. Fig. S2. Heterogeneous expression of miR-125b in patient tumor samples and breast cancer cell lines. Fig. S3. Stratification of breast cancer patients based on expression levels of miR-125b, IGFBP2 and CCL28. Fig. S4. Relative survival analysis of miR-125b-associated behavior of breast cancer cell lines and xenografted tumors. Fig. S5. Mechanism of miR-125b-driven fitness disadvantage of cancer cell altruism. Fig. S6. Mechanism of miR-125b-driven fitness benefit of cancer cell altruism. Fig. S7. Heightened co-expressions of oncogenes and tumor suppressors in miR-125bHigh altruists. Fig. S8. MiR-125bHigh altruists are regenerable via a KLF2-mediated acetylation. Fig. S9. ChIP reveals protein-chromatin interactions at two different KLF2 binding sites. Fig. S10. Lateral inhibition maintains a sparse spatial organization of altruists. Table S1. Clinical characteristics of breast cancer patients for miRNA profiling of CTCs (Supplementary Fig. 1). Table S2. Clinical characteristics of breast cancer patients for miR-125b in-situ hybrid [file 12943_2023_1896_MOESM1_ESM.docx]

**Dynamic altruistic cooperation within breast tumors**

Muhammad Sufyan Bin Masroni, Kee Wah Lee, Victor Kwan Min Lee, Siok Bian Ng, Chao Teng Law, Kok Siong Poon, Bernett Teck-Kwong Lee, Zhehao Liu, Yuen Peng Tan, Wee Ling Chng, Steven Tucker, Lynette Su-Mien Ngo, George Wai Cheong Yip, Min En Nga, Susan Swee Shan Hue, Thomas Choudary Putti, Boon Huat Bay, Qingsong Lin, Lihan Zhou, Mikael Hartman, Tze Ping Loh, Manikandan Lakshmanan, Sook Yee Lee, Vinay Tergaonkar, Huiwen Chua, Adeline Voon Hui Lee, Eric Yew Meng Yeo, Mo-Huang Li, Chan Fong Chang, Zizheng Kee, Karen Mei-Ling Tan, Soo Yong Tan, Evelyn Siew-Chuan Koay, Marco Archetti & Sai Mun Leong

Corresponding author: [patlsm@nus.edu.sg](mailto:patlsm@nus.edu.sg) (SML), [mua72@psu.edu](mailto:mua72@psu.edu) (MA), [patkoaye@nus.edu.sg](mailto:patkoaye@nus.edu.sg) (ESCK), [pattsy@nus.edu.sg](mailto:pattsy@nus.edu.sg) (SYT), [karen_ml_tan@nuhs.edu.sg](mailto:karen_ml_tan@nuhs.edu.sg) (KMLT)

**The PDF file includes:**

Supplementary Notes 1 to 3

Figs. R1 to R9

Figs. S1 to S10

Tables S1 to S5

References (*67*–*80*)

**Supplementary Note 1: Definition of altruism**

To define the nature of interaction between the miR-125b^m^ and Control^m^ cells in docetaxel-treated “heterogeneous” mixture (Fig. 1A-Q, Fig. S4A-E), we adapted a four-way classification matrix of social behavior from Hamilton [30] and West *et al*. [13], an example of which is shown in Fig. R1 below.

**Fig. R1. Social matrix of interaction.** From Hamilton (1964) [30] and West *et al*. (2006) [13].

Social behaviors are classified by Hamilton (1964) [30] depending on whether the consequences to the donor and recipient are beneficial or costly [13]. This classification has been highly useful and influential in the field of evolutionary biology, and is applied to understand, for example, adaptation in pathogenic microbes [13,67–69], spiteful behavior in polyembryonic parasitoid wasps [70] and social traits in yeast [71]. Adapting this matrix to our study, we assessed the gains (benefits) and losses (costs) using two parameters: (a) Relative Survival and (b) Relative Fitness.

(a) Relative Survival (RS): measures the number of surviving Control^m^ (RS_α_) or miR-125b^m^ (RS_β_) cancer cells in the “heterogeneous” mixture, relative to the number of surviving Control^m^ cancer cells in either of the two fractions present the “homogeneous” mixture, four days after a 12 h exposure to docetaxel (Fig. 1H-J).

Here,

RS_α_ = $\frac{Number of surviving Control\text{m} cancer cells in "heterogeneous" mixture}{Number of surviving Control\text{m} cancer cells of either fraction in "homogeneous" mixture}$

RS_β_ = $\frac{Number of surviving miR-125b\text{m} cancer cells in "heterogeneous" mixture}{Number of surviving Control\text{m} cancer cells of either fraction in "homogeneous" mixture}$

RS_α_ provides an indication of how the presence or absence of miR-125b^m^ cancer cells affects the immediate survival of Control^m^ cancer cells in the “heterogeneous” mixture after taxane treatment. RS_α_ > 1 indicates that Control^m^ cancer cells in the “heterogeneous” mixture survive better than their counterparts in the “homogeneous” mixture, and this may be attributed to the presence of miR-125b^m^ cancer cells in the “heterogeneous” mixture. Conversely, if RS_α_ < 1, this indicates that the presence of miR-125b^m^ cancer cells negatively affects the survival of partner Control^m^ cancer cells in the “heterogeneous” mixture.

(b) Relative Fitness (RF): measures the number of surviving Control^m^ cancer cells, relative to the miR-125b^m^ cancer cells cohabiting in the “heterogeneous” mixture (RF_α_) in the fully confluent population that is regrown from survivors of docetaxel treatment, and vice versa (RF_β_). Surviving cells reached 100% confluency in 1-3 weeks’ time, depending on the cancer cell lines used and docetaxel concentrations the cells were previously exposed to. In essence, RF measures the relative ability of the Control^m^ versus miR-125b^m^ cancer cells to survive taxane treatment and give rise to the subsequent generations of daughter cells.

Here,

RF_α_ = $\frac{Number of surviving Control\text{m} cancer cells in "heterogeneous" mixture}{Number of surviving miR-125b\text{m} cancer cells in "heterogeneous" mixture}$

RF_β_ = $\frac{Number of surviving miR-125b\text{m} cancer cells in "heterogeneous" mixture}{Number of surviving Control\text{m} cancer cells in "heterogeneous" mixture}$

Thus,	RF_α_ = 1/ RF_β_

An RF_α_ value of >1 indicates that Control^m^ cancer cells dominate the fully confluent population expanded from the initial “heterogeneous” mixture that survived docetaxel treatment. An RF_α_ value of <1 indicates otherwise, i.e., the miR-125b^m^ cancer cells dominate the regrown population.

We define the nature of the interaction between cohabiting miR-125b^m^ and Control^m^ subpopulations in the “heterogeneous” mixture using these two parameters (Fig. 1R):

(i) **RF_α_ = RF_β_ = 1, RS_α_ > 1, RS_β_ > 1 ⇨ “Mutually beneficial”** (Fig. 1R lower left quadrant): A “win-win” situation as both Control^m^ and miR-125b^m^ subpopulations in the “heterogeneous” mixture survive better than expected (when compared to their Control^m^ counterparts in the “homogeneous” mixture”) immediately post-chemotherapy. Neither subpopulation dominates over each other in the regrown population.

(ii) **RF_α_ = RF_β_ = 1, RS_α_ < 1, RS_β_** **< 1 ⇨ “Spiteful”** (Fig. 1R upper right quadrant): A “lose-lose” situation as both Control^m^ and miR-125b^m^ subpopulations in the “heterogeneous” mixture survive worse	than expected (when compared to their Control^m^ counterparts in the “homogeneous” mixture”) immediately post-chemotherapy. Neither subpopulation dominates over each other in the regrown population.

(iii) **RF_α_ < 1, RF_β_ > 1, RS_α_ ≤ 1, RS_β_ > 1 ⇨ “Selfish”** (Fig. 1R lower right quadrant): miR-125b^m^ subpopulation survives better than expected (when compared to its Control^m^ counterparts in the “homogeneous” mixture”) immediately post-chemotherapy. This may happen e.g. due to activation of pro-survival or anti-apoptotic pathways in miR-125b^m^ cells with heightened level of miR-125b. The cohabiting Control^m^ cells do not benefit from the presence of their miR-125b^m^ cohabitants with taxane exposure, and their survival immediately after drug exposure may even be negatively affected by the latter, thus RS_α_ is ≤ 1. The miR-125b^m^ cells eventually dominate the regrown population.

(iv) **RF_α_ > 1, RF_β_ < 1, RS_α_ ≥ RS_β_, RS_α_> 1 ⇨ “Altruistic”** (Fig. 1R upper left quadrant): Control^m^ subpopulation survives better than expected (when compared to its Control^m^ counterparts in the “homogeneous” mixture”) immediately post-chemotherapy, due to benefits offered by the cohabiting miR-125b^m^ cells, e.g. in the form of soluble proteins that induce chemotolerance in the Control^m^ recipients. Due to the fitness cost associated with high miR-125b expression, the miR-125b^m^ cells eventually lose their dominance to partner Control^m^ cells in the regrown population, thus rendering this interaction as being “altruistic”.

Fitting the data in our study (Fig. 1A-Q, Fig. S4A-E) to our matrix for classification of social behavior (Fig. 1R), it appears that the interaction between cohabiting miR-125b^m^ and Control^m^ in the “heterogeneous” mixture is in reminiscence of altruism as defined in social evolution theory. “Altruism” in our study is defined in an evolutionary sense – i.e. as “biological altruism”, a behavior that benefits others at the cost to self, with the cost and benefits measured in terms of reproductive fitness or expected number of offspring [13]. This is different from our everyday concept of “altruism”, typically defined as “an action performed with a conscious intention to help others” [72].

For altruistic interaction, if RS_β_ is also >1 (but ≤ RS_α_), this means that during early post-treatment, miR-125b^m^ cells may self-benefit from the soluble proteins they produce, as in the case for volunteer’s dilemma [73] and continuous snowdrift game [74]. This may happen due to both miR-125b^m^ and Control^m^ cells harboring the same cell surface receptor for binding the soluble proteins, as demonstrated by the presence of comparable levels of CCR10 receptor for binding of CCL28 in both Mi/EGFP^High^ and Mi/EGFP^Low^ cells in our study (Fig. S6J). It may be argued that since the miR-125b^m^ cells appear to self-benefit from the extracellular proteins they produced, the interaction between the two cohabiting fractions may alternatively be modelled as one-way by-product mutualism, in which Control^m^ cells derive “accidental” benefits from miR-125b^m^ cells due to close proximity to the latter. However, it was previously shown in a “secrete-and-sense” model that at high cell density, it is heightened secretion rate, rather than a reduced receptor expression, of the secreting cells that promotes neighborly communication over self-communication [43]. This may explain why CCR10 expression is not down-regulated in the altruistic cancer cells in order to minimize self-communication. The altruists may thus still be able to self-benefit to a certain extent, though not as much as how the cohabiting defectors benefit from the altruistic interaction during early post-treatment and at the high cell density condition typically observed in tumors. We also observed heightened levels of secreted proteins in the miR-125b^High^ cells with docetaxel exposure (Fig. 2I) and this might have helped to contribute to enhanced neighborly communication between the cohabiting altruists and defectors, as shown in the “secrete-and-sense” model [43]. We also demonstrated that while altruists-secreted IGFBP2 and CCL28 can induce GAB1-dependent PI3K/AKT activation in the neighboring recipient cancer cells and likely lead to heightened survival of these cells with docetaxel exposure, self-PI3K/AKT activation in the altruists are likely to be blunted due to direct inhibition of GAB1 by the high level of miR-125b in the altruists (Fig. 4L). In view of these evidence, and our empirical observations that the Control^m^ cells dominated the “heterogeneous” mixture in the regrown population monitored over a longer period (RF_α_ > 1), we modelled the interaction between miR-125b^m^ and Control^m^ cells as “altruistic”.

Another scenario deserving careful consideration involves the potential emergence of a miR-125b^Low^ subpopulation from miR-125b^High^ altruistic cells (and vice-versa) in response to initial exposure to docetaxel. This could constitute an independent action occurring within a heterogeneous and dynamic cell population and was not addressed by our key co-culture experiment using ectopically expressing cell lines (Fig. 1). This phenotypic transition may occur through the KLF2-mediated epigenetic mechanism (Fig. 3), enabling the newly formed miR-125b^Low^ fraction to directly benefit from trophic factors secreted by the altruistic cells. Within this context, any neighboring miR-125b^Low^ cells would only incidentally reap the rewards of the trophic factors due to their proximity, thus categorizing the relationship as commensalism rather than altruism. Commensalism signifies a relationship where one party gains benefits from the other without either party causing harm or offering any particular advantage in return. However, as shown in Fig. S8I, we observed that purified Mi/EGFP^High^ cells did not give rise to distinct subpopulations of Mi/EGFP^Low^ during the docetaxel treatment period, thus dispeling the possibility of commensalism. We also noted that purified Mi/EGFP^Low^ fraction did not give rise to its own Mi/EGFP^High^ altruists during the treatment period (Fig. S8I), suggesting that in the event of docetaxel exposure, miR-125b^Low^ cells will need to depend on pre-existing miR-125b^High^ altruists in the vicinity for their survival.

While the value of RF_α_ (if >1) alone appears to be sufficient in defining the interaction as being “altruistic”, it is not actually so. This is because the possibility that “RF_α_ > 1, RS_α_ = 1” exists. In this hypothetical scenario, the miR-125b^m^ cells exert no influence over the Control^m^ cells (and vice versa), yet the latter may still survive through sub-lethal dosage of docetaxel, though the immediate post-treatment survival rate is not better than expected when compared to its Control^m^ counterparts in the “homogeneous” mixture (resulting in RS_α_ = 1). Due to a faster growth rate, the Control^m^ cells may subsequently dominate over the miR-125b^m^ cells in the regrown population, leading to RF_α_ > 1, though there was no actual interaction of any nature occurring between the two cancer cell fractions during initial drug exposure. As such, in describing an interaction between two cell subpopulations as being “altruistic”, it is important that the two fundamental criteria (RF_α_ > 1, RS_α_ > 1) be satisfied at the same time. The interaction is adjudged to be “altruistic” regardless of whether miR-125b^m^ cells initially self-benefit or not with respect to immediate post-treatment survival – thus in our study, we only reported the values of RF_α_ and RS_α_, and omitted the reporting of RS_β_ for all cell lines and xenograft model studied (Note: RF_β_ was omitted too, as it is the reciprocal of RF_α ._ The value of RF_β_ can be deduced when value of RF_α_ is known).

**Supplementary Note 2: Modeling based on evolutionary game theory predicts persistence of altruistic subpopulation**

**Survival of the fittest or tragedy of the commons?**

Consider a population of cells in which a mutant cell produces a beneficial molecule that confers resistance against a drug. During therapy, the clonal lineage arising from this mutant will have a selective advantage and expand within the tumor by clonal selection, in a way analogous to Darwinian natural selection by “survival of the fittest” [1] (Fig. R2). If the beneficial molecule is secreted, a population of producer cells will have higher fitness than a population of non-producer cells during drug exposure. However, in this case, a non-producer arising within a population of producer cells will have a higher fitness than producer cells, because it can free-ride on the molecules secreted by its producer neighbors without paying the cost (Fig. R2). This raises a conceptual problem: why aren’t producer cells driven to extinction? This is an instance of a well-known general phenomenon with collective interactions, generally referred to as “tragedy of the commons” [48]: how can cooperation for the production of a common good evolve in a population of self-promoting individuals? The idea that clonal selection among cancer cells is driven by the maximization of tumor fitness is equivalent to the idea [75], now discredited in evolutionary ecology, that natural selection is driven by the maximization of the benefit of a group, population or a species. Instead, evolutionary dynamics is driven by the maximization of individual fitness, which in the case of tumors is the fitness of individual cancer cells. Invoking the common good of the population (in our case, the tumor) is not a tenable explanation, as in clonal selection, like in natural selection, nothing evolves for the benefit of the group or population [4,28]: non-producers have a higher fitness when surrounded by producers because they can exploit the public good at no cost; hence non-producers should increase in frequency within the population, driving producers to extinction and the population itself to eventually collapse – a tragedy of the commons at the cellular level.

**Fig. R2. Survival of the fittest and the tragedy of the commons within tumors.** When resistance to a drug is due to a cell-autonomous mutation, the drug-resistant subclone has higher fitness, and exposure to the drug leads to its expansion by clonal selection and to the extinction of the non-resistant subclone. When resistance is due to a secreted factor, instead, non-producer clones (defectors) can survive by free-riding on their producer (altruistic) neighbors without paying its production cost; hence they could outcompete the altruistic subclone leading to its extinction and to the loss of the common benefit of chemotolerance (“tragedy of the commons”). We found instead that the producer cells secreting beneficial factors persist at low frequency in the population (“altruism”), leading to a stable population-wide chemotolerance.

We use evolutionary game theory to explain why, in our system (and presumably in other analogous cases), selection leads to a stable coexistence of the two types with a minority of producer cells and why the fraction of producer cells increases with the amount of therapy (impairing the effect of the therapy). Unraveling the dynamics of the system leads to the intuition that an epigenetic mechanism is necessary to maintain cooperation, which is indeed what we observed.

**Dynamics in well-mixed populations**

A cell can be an altruist (producer: miR-125b^High^) or a defector (non-producer: miR-125b^Low^). In a large, well-mixed population, at each generation, the probability of having *i* altruists among the *n* cells with the diffusion range of the secreted public goods, given a frequency *x* of altruists in the population*,* is given by the probability mass function of a binomially distributed random variable *i* with parameters (*n*−1, *x*):

$$p_{i,n-1}(x) = \binom{n-1}{i}x^{i}\left( 1-x \right)^{n-1-i}$$

(1)

The fitnesses of altruists and defectors are therefore given by

$$W_{C} = \sum_{i = 0}^{n-1} p_{i,n-1}(x)\cdot b\left( i+1 \right)-c$$

(2)

$$W_{D} = \sum_{i = 0}^{n-1} p_{i,n-1}(x)\cdot b\left( i \right)$$

(3)

where *b*(*i*) is the benefit (payoff) for being in a group with *i* other altruists. This means that, as is standard in evolutionary game theory models, fitness is the weighted sum of the benefit achieved in different groups with different fractions of altruistic cancer cells (weighted by the frequency of each group in the population). In any given group, a non-producer cell will always find itself with 1 altruist less (itself) than a producer cell; but the producer cell will always pay a cost *c* (0<*c*<1; assuming a maximum benefit equal to 1, *c* is the cost/benefit ratio of altruistic cooperation) that the non-producer cell does not pay.

The average fitness of a mixed population is

$$\bar{W} = x\cdot W_{C}+(1-x)\cdot W_{D}$$

(4)

The replicator dynamics of this system is

$\dot{x} = x \left( 1-x \right) B\left( x \right)$

(5)

where the fitness difference *W_C_*-*W_D_* is written as

| *B*(*x*) = ${\sum_{i = 0}^{n-1} p}_{i,n-1}(x)\cdot\Delta b_{i}$ | (6) |
| --- | --- |
|  |  |

the gradient of selection, where

$$\Delta b_{i} = b\left( i+1 \right)-c-b(i)$$

(7)

If *b*(*i*) is a linear function of the number of altruists (that is, the effect of altruistic cooperation is additive), then *b*(*i*)= *rci/n* (where *r* is a multiplying enhancing factor for the effect of altruistic cooperation). It is well known [76] that, in this case, unless *r* >*n* (which implies an unrealistically high enhancing factor or an unrealistically small group size), the only stable equilibrium (found by setting *W_C_*=*W_D_*) is *x*=0, that is, the collapse of cooperation (the “tragedy of the commons”).

**Non-linear benefits**

In our case, however, the benefit *b*(*i*) is a non-linear function of the number of altruists (that is, the effect of altruistic cooperation is not simply additive) (Fig. S6A). While with linear benefits producers always have a lower fitness than non-producers, with non-linear effects the benefit for a producer can be higher than for a non-producer at intermediate frequencies of producers (if the benefit is sigmoid) or at low frequencies (if the benefit is concave) (Fig. R3).

**Fig. R3. Frequency-dependent selection.** Each plot shows the effect of altruism on a cell type (yellow: defector; blue: altruistic; dotted line: benefit for the altruistic cell if the cost is zero) as a function of the number (*i*) of altruistic cells. Altruistic cells benefit from 1 altruistic cell (itself) more than defector cells, hence they have a higher benefit *b*(*i*+1); but they also pay an extra cost *c* that defector cells do not pay.

We can model this nonlinear benefit function as

$$b\left( i \right) = \frac{\left[ l\left( i \right)-l\left( 0 \right) \right]}{\left[ l\left( n \right)-l\left( 0 \right) \right]}$$

(8)

the normalized version of a logistic function *l*(*i*) with inflection *h* and steepness *s*:

$$l\left( i \right) = \frac{1}{1+e^{s(h-i/n)}}$$

(9)

The parameter *h* defines the position of the inflection point: *h*→1 gives increasing returns (convex) and *h*→0 diminishing returns (concave); *s* defines the steepness of the function (*s*→∞ models a threshold public goods game; *s*→0 models an N-person Prisoner’s Dilemma” (NPD); the normalization in equation (9) prevents the logistic function from becoming constant for *s*→0). In this case, beyond the two trivial pure equilibria *x* = 0 and *x* = 1, further mixed equilibria can be found by setting *B*(*x*)=0. While in this case the equilibrium cannot be found analytically, the equilibrium frequency of the fraction of altruists can be approximated by:

$$x_{eq}=h-\frac{1}{s}\mathrm{Log}[-1+\frac{s-\sqrt{-(4cn-s)s}}{2cn}]$$

(10)

and we can characterize the dynamics resorting to the properties of Bernstein polynomials, which essentially enable us to analyze the coefficient $\Delta b_{i}$ rather than the full gradient of selection *B*(*x*) [77]. Games with sigmoid benefits (or any benefit function with only one inflection point) can have two internal equilibria, one of which stable, and two pure equilibria (unstable pure altruistic

**Fig. R4. Dynamics of altruism.** Different types of benefit functions (top panels) and their corresponding replicator dynamics (the difference in fitness between altruists and defectors minus the cost of altruism; bottom panels) are shown, as a function of the fraction of altruistic cells. From left to right: linear (*s*→0), concave (*h*=0.1, *s*=4), convex (*h*=0.9, *s*=4), sigmoid (*h*=0.3, *s*=10) benefits. The circles show the equilibria (full: stable; empty; unstable); the arrows show the direction of the dynamics. The horizontal dotted line shows the cost (*c*=0.03); *n*=30

cooperation; stable pure defection) [77] (Fig. R4)**;** a single stable polymorphism exists which producer and non-producer cells coexist, if the shape of the benefit function is concave, as is in our case (Fig. S6A).

**Estimating the parameters**

The cost/benefit ratio of cooperation can be estimated from the growth, after the same time, of pure populations seeded at equal densities (Fig. 1S): the cost is estimated by the difference in growth between altruistic cells and defector cells in the absence of taxane (because in that case, there is no benefit – hence the only difference in growth must be due to the cost of non-cell-autonomous secretion by the altruistic cells); the benefit is estimated by the difference in growth between defector cells in the absence */* presence of taxane (because in that case the only difference in growth must be due to the absence / presence of taxane). Hence *c/b*= (D_0-_C_0_) */* (D_0-_C_T_), where D_0_ is the number of defector cells in the absence of taxane; C_0_ is the number of altruistic cells in the absence of taxane; C_T_ is the number of altruistic cells in the presence of taxane. Using this method, the estimated cost benefit ratio (*c/b*) is 0.31. An alternative method to estimate the cost and benefit, as well as the shape of the benefit function (the parameters *s* and *h*) is by fitting the relative growth of mixed populations (Fig. S6A), to the function for the average fitness of mixed populations (equation 4). For example, with *n=*30 (*n* cannot be estimated from the model) the parameters are estimated to be *c/b*=0.31 (remarkably in line with the result obtained in pure populations using the method described above), *s*=18 and *h*=0.01. Using different values of *n* does not change the results significantly, always yielding a concave benefit function (Fig. R5). Given these parameters, we predict an equilibrium value of the fraction of altruists close to the to the empirical observations (Fig. R6).

**Fig. R5.** **Estimating the parameters.** (A) The parameters of the model (*c*, *s*, *h*; assuming *b*=1) estimated as a function of *n* (the number of cells in the diffusion range of the secreted altruistic molecules)*.* (B) The benefit function (equation 8) given by the parameters estimated for different values of *n* from 10 to 1000. (C) Average fitness of a mixed population (black curve), of altruists (blue dashed curve) and defectors (yellow dashed curve) as a function of the fraction of altruists (equation 4), fitted to the relative growth rates of mixed populations (from Fig. S6A: black dots are the average values of the individual measures, in gray); the parameters used are the ones estimated from the data (*c/b*=0.3*, s*=18, *h*=0.01; assuming *n=*20). (D) The fitness functions for different values of *n* (10, 20, 30).

Evolutionary dynamics shows, therefore, that the stability of chemotolerance induced by a minority of altruistic cancer cells is not maintained because it increases the average fitness of the tumor but can be explained by the logic of competition under clonal selection: because chemotolerance is due to a secreted molecule with non-linear effects, selection is frequency-dependent: the benefit of being an altruist is higher at low frequencies of cooperators. In that case, the altruists increase in frequency in the population; altruists do not go to fixation, however, because as their fraction increases, the benefit of altruistic cooperation declines, and defectors have a higher fitness above a critical fraction of altruists (in that case, altruists decline in frequency). Hence a stable coexistence is expected, and the equilibrium value of altruistic cooperation does not depend on the initial frequencies of the two types, as we indeed observe in our system. Clonal selection, therefore, still promotes the survival of the fittest in our system, but producer cells, which have higher fitness in pure population, are prone to being invaded by non-producer cells. On the other hand, a tragedy of the commons does not occur (altruists do not go extinct) because the effect of altruistic cooperation is a non-linear (concave) function of the fraction of altruists.

**Why increasing therapy leads to more altruistic cooperation**

Because the stable fraction of altruists at equilibrium is a decreasing function of the cost/benefit ratio (the first derivative of equation (10) with respect to *c* is always negative), we also expect the

| $-\frac{\frac{s}{c\sqrt{s(-4cn+s)}}-\frac{s-\sqrt{s(-4cn+s)}}{2c^{2}n}}{s\left( -1+\frac{s-\sqrt{s(-4cn+s)}}{2cn} \right)}$ | (11) |
| --- | --- |

equilibrium fraction of altruists to increase with the benefit of altruistic cooperation (that is, with the amount of taxane). This is true for any value of the cost and any shape of the benefit function, as long as it is increasing and monotonic.

Therefore, the theory also explains why increasing the amount of therapy may lead to relapse: because it reduces the cost*/*benefit ratio of altruistic cooperation, which leads to a higher equilibrium fraction of altruists (Fig. R6). Increasing the amount of taxane increases the benefit of secreting diffusible molecules that promote increased chemotolerance (the lower the therapy, the lower the benefit; with no therapy, there is no benefit for cooperation). Increasing the benefit of altruistic cooperation reduces the cost/benefit ratio of being an altruist (if the cost remains fixed). This leads, as we have seen, to a higher fraction of altruists at equilibrium.

**Fig. R6.** **The observed saturating effect leads to a stable minority of altruistic cancer cells.** (A) The difference in fitness between altruistic and defector cells for the parameters estimated (*c*=0.3, *s*=18, *h*=0.01 and different values of *n* (from 10 to 30); the fraction of altruistic cancer cells increases when the fitness difference is positive and decreases when negative; arrows show the direction of the dynamics; circles show stable mixed equilibria (where the fitness of the two types is equal). (B) The fitness difference and the dynamics for the same parameters (*c*=0.3; *s*=18; *h*=0.01) with *n*=30 and *b*=1 (blue curves) and for increasing or decreasing values of *b* (gray curves; *b* values, from 0.5 to 10).

**Supplementary Note 3: Dynamics in spatially structured populations hints at epigenetic mechanism in mediating persistence of altruists**

Spatial structure is known to have an effect on the evolution of cooperation [78], therefore we repeated the analysis using a model in which cells are arranged in a spatial structure resembling a monolayer. Individual cells occupy all individual nodes of a planar graph (a Voronoi network, drawn on a sphere to avoid edge effects, which has previously been used to model monolayers of cancer cells [79]), in which the interaction neighborhood and the update neighborhood are decoupled: a cell’s group (of size *n*) is not limited to its one-step neighbors but is defined by the diffusion range *D* of the molecule. Group size for a cell is given by the number of cells within *D* nodes from that cell. We assume that each cell receives contributions from other producer cells within a diffusion range *D* of the molecule, each contribution weighted by the distance *j* (the number of nodes) from that cell according to the function *G*(*j*)=1-[*g*(*j*)-*g*(0)]/[*g*(*D*)-*g*(0)], where *g*(*j*)=1/[1+*e*^-^*^z^*^(^*^j-d^*^)/^*^D^*]. The relative values of *d* and *D* determine the shape of the diffusion gradient, which is always decreasing. The parameter *z* controls the steepness of the gradient at the inflection point.

A cell’s payoff is a function of the amount of factor produced by the group it belongs to. The process starts with a number of non-producer cells placed on the graph. We use a standard stochastic update rule in the framework of evolutionary game theory [27,78,79] to model clonal selection: at each round a cell *x* with a payoff P*_x_* is selected (at random) for update (death); a cell *y* (with a payoff P*_y_*) is then chosen among *x’*s neighbors; replacement occurs with a probability given by (P_y_-P_x_)/M, where M ensures the proper normalization and is given by the maximum possible difference between the payoffs of *x* and *y*. Results are obtained averaging the final 200 of 1000 generations per cell, averaged over 10 different runs.

We found that altruistic cooperation is unstable when benefits are concave – unlike the case of linear benefits in which spatial structure promotes cooperation [78]. To understand why, we repeated the analysis using, instead of the standard update rule of evolutionary dynamics (which models a birth-death process analogous to what is observed when cooperation is determined genetically: a cell is killed and replaced with the progeny of one of its neighbors), an update rule equivalent to the best-response dynamics generally used to model interactions among rational payoff-maximizing individuals. While clearly no rational decision making occurs in cancer cells, a best-response dynamics can be used to model changes in the frequency of the two types due to a hypothetical epigenetic mechanism: the type at each node is decided at each generation based on the highest payoff a cell can obtain in that group, rather than by the standard process of evolutionary dynamics (that assumes the cooperative phenotype is genetically determined) [79]. Such optimized cancer cell dynamics is not unique to our situation - a stochastic optimal version of cancer cellular dynamics has recently been studied in detail with regards to tumor antigenicity, supporting the observation of optimal decision-making in cancer populations [80]. With this best-response update rule, we do observe convergence to stable cooperation in which altruists and defectors coexist, when benefits are concave (Fig. R7), reconciling our result with the logic we discussed in the case of well-mixed populations. Similar to what we have found for well-mixed populations, increasing the benefit of altruistic cooperation (reducing the relative cost/benefit ratio) leads to a higher proportion of altruistic cells – a response to treatment that, in the case of epigenetic determination, is even faster than in the case of genetic cooperation (Fig. R8).

**Why altruistic cooperation requires epigenetic determination**

The reason why an epigenetic mechanism enables altruistic cooperation (while genetic determination does not) becomes clear upon inspection of the fitness of clusters of altruists and defectors in a spatially structured population (Fig. R9).

In the case of genetic determination, clonal selection can produce changes in the fraction of the two types only when there is some one-step neighbor with a different genotype, which generally occurs at the edge between clusters of the two types; no changes can occur in the centre of clusters of cells of the same type. When benefits are concave, defectors always have higher fitness than altruists at the edge between clusters of producers and non-producers (where clonal selection produces changes in the fraction of the two types), which leads to the collapse of altruistic cooperation under the standard update rule of evolutionary dynamics (with sigmoid benefits, however, cooperation can persist [79]. In a best-response dynamics, however, equivalent to the epigenetic mechanism described here, changes in the relative fractions of the two types are not limited to the edges of clusters of altruists and defectors; one type can turn into the other even at the center of clusters of cells of the same type. Hence, altruists can arise within clusters of defectors – the fraction of the two types is kept approximately constant because each cell can change its type based on the prevalence of types within the diffusion range of the public goods. As a consequence, best-response dynamics or epigenetic mechanisms acting on a monomorphic population can maintain altruistic cooperation with concave benefits, as in the case we reported here.

**Fig. R7. Epigenetic determination enables stable altruism.** Altruism can be due to standard clonal selection (“genetic”) or based on a best-response dynamics (“epigenetic”). Genetic altruism can evolve only if benefits are sigmoid (*h* around 0.5) and when the cost is not too high; with concave benefits (low *h*) only epigenetic altruism is stable. (A) Changes in the fraction of altruistic cancer cells and fitness over time in simulations with the parameters estimated for the system (*h*=0.01; *s*=18; *c/b*=0.31) and D=6, *z*=30, for genetic and epigenetic altruism. (B) Stable fraction of altruistic cancer cells and fitness at equilibrium for genetic and epigenetic altruism for given pairs of *c* (the cost of altruism, assuming *b*=1) and *h* (the inflection point of the benefit function; the function is concave for low *h*) in simulations with the value of *s* (the steepness of the benefit function) estimated for the system and *d*=3. (C) An example of a monolayer of cells at equilibrium with epigenetic determination of altruism. Clusters of defectors (yellow) can persist because of the public goods secreted by a few altruistic cells (blue); clusters of too many defector cells, however, have such low fitness (pale yellow areas) that an altruistic cell will have higher fitness there.

**Fig. R8. Increasing therapy leads to more altruistic cooperation.** Change in the fraction of altruists over time in a spatially structured population (epigenetic update). The benefit of altruistic cooperation *b* is initially 1 and doubled after 100 generations (when therapy is increased); *c*=0.06; *s*=20; *h*=0.01; D=6; *z*=30. Initially, a low dosage of the drug leads to a stable fraction of altruistic cancer cells. When the amount of therapy is increased (after 100 generations), the fraction of altruists increases (because the cost/benefit ratio has declined.

**Fig. R9. Why epigenetic-regulated altruism can persist.** At the edge between clusters of altruists (blue cells) and defectors (yellow cells), there is always a mixture (approximately 50%) of the two types; if the benefit of altruistic cooperation is concave (the inflection point *h* of the fitness functions is low) at the edge (for example at position A), defectors have higher fitness than altruists, therefore they replace altruists and the defector cluster continuously advances until altruistic cooperation becomes extinct. At position B, on the other hand, an altruist would have a higher fitness than a defector due to its rarity, but there are no altruistic neighbors that can replace, by clonal selection, the defector cell at B. Only an epigenetic mechanism can enable a cell at position B to readily become an altruist when surrounded by defectors.

**Fig. S1.**

**B**

**C**

**E**

**H**

**J**

**I**

**F**

**A**

**G**

**D**

**Fig. S1. microRNA expression in CTCs from breast cancer patients undergoing taxane treatment.** (**A-J**) Fold differences in expression level of miR-125b and other microRNAs in CTCs isolated from breast cancer patients undergoing taxane-based chemotherapy were monitored.

Upper: CTC number. Red arrows indicate time point during the monitored period where relatively higher miR-125b expression was detected in CTCs (A-G). Middle : expression level of various miRNAs in CTCs. Grey dots indicate fold differences of each of the two replicates. Lower : representative images of isolated CTCs, scale bar = 15 μm. Both CTCs and white blood cells were stained with nuclear stain Hoechst 33342 (blue), while white blood cells stained positive for CD45 (green). Technical replicates = 2 for each PCR reaction of each miRNA at each time point.

**A**

**B**

**C**

**E**

**D**

**F**

**G**

**Fig. S2.**

**Fig. S2. Heterogeneous expression of miR-125b in patient tumor samples and breast cancer cell lines.** (**A**) Increased number of MDA-MB-231^miR-125bprom-EGFP^ cells with heightened Mi/EGFP expression with docetaxel exposure. Left: Images of live cells with or without docetaxel exposure. Right: bar chart depicting percentage of cells with high Mi/EGFP expression, as measured by FACS. (**B**) Immunohistochemical staining of Mi/EGFP in MDA-MB-231^miR-125bprom-EGFP^ spheroids. Black arrows indicate spheroidal region with increased staining (brown) for Mi/EGFP. (**C**) Immunohistochemical detection of Mi/EGFP (brown) in xenograft tumor from mice. inoculated with MDA-MB-231^miR-125bprom-EGFP^, without (Upper) or with (Lower) docetaxel treatment. Sections were counterstained with hematoxylin. (**D**) Levels of miR-125b expression (as visualized using miRNA *in situ* hybridization) and Mi/EGFP (as visualized using immunohistochemistry), shown here to correlate with each other. Left: images of MDA-MB-231^miR-125bprom-EGFP^ cells, after being subjected to both miRNA *in situ* hybridization and immunohistochemistry. Right: dot plot of respective staining intensities. (**E**) Histograms of MDA-MB-231^miR-125bprom-EGFP^ cells that were either untreated or exposed to 5 nM docetaxel for 12 hr (Left). Cells surviving drug exposure were allowed to grow for 8 weeks (drug holiday) before being exposed to 5 nM docetaxel again. Histograms for post-chemotherapy holiday cells that were untreated or exposed to 5 nM docetaxel are shown on the right. Docetaxel-induced increase in Mi/EGFP^High^ cells (Left bottom) diminished after the chemotherapy holiday (Right top), though the cells retained the ability to increase the proportion of Mi/EGFP^High^ cells upon re-treatment with docetaxel (Right bottom). Mi/EGFP^High^ cells remained a minority in the population, even after repeated exposure to docetaxel. (**F**) RT-qPCR was performed on various breast cancer cell lines used for this manuscript. Fold difference in miR-125b expression for each cell line was derived with respect to miR-125b expression in MCF-7. MiR-125b expression in MCF10A, non-tumorigenic breast epithelial cell line, is included here for comparison. MiR-93 expression, chosen from amongst several miRNAs due its relative stability in expression levels across all cell lines, was used to normalize expression levels of miR-125b across the difference cell lines. (**G**) Fluorescence images showing heterogeneous expression of Mi/EGFP, a reporter for miR-125b expression, in indicated cell lines. Nuclei were visualized using Hoechst 33342.

Data are mean ± s.d. from three independent biological triplicates (A). Experiment repeated three (B) or two (E) times, representative data shown. Experiment repeated with three independent mice per treatment group, representative data shown (C). Statistical analysis was performed using Pearson’s correlation (D). NT: no treatment; DTX: docetaxel treatment.

**Fig. S3.**

**B**

**C**

**D**

**E**

**I**

**G**

**A**

**F**

**H**

**Fig. S3. Stratification of breast cancer patients based on expression levels of miR-125b, IGFBP2 and CCL28.** (**A-D**) Kaplan-Meier survival curves, as generated by Kaplan Meier plotter (www.kmplot.com), for overall survival of chemotherapy-treated (A, B) or untreated (C, D) breast cancer patients, stratified by expression levels of miR125b (A, C) or miR-125a-5p (B, D). Unlike miR-125a-5p, high expression of hsa-miR-125b is associated with significantly reduced overall survival for chemotherapy-treated breast cancer patients (B). The same trend could not be observed for treatment-naïve patients for both miRNAs. Despite miR-125a-5p and miR-125b being homologues of the miR-125 miRNA family, both exhibits markedly different stratification patterns. (**E-I**) Kaplan-Meier survival curves for overall survival (OS) (E,G) and distant-metastasis-free survival (DMFS) (F,H) for neoadjuvant chemotherapy-treated breast cancer patients, stratified by levels of IGFBP2 (E,F) and CCL28 (G,H). Apart from resulting in increased secretion of CCL28 and IGFBP2 proteins from docetaxel-exposed breast cancer cells, heightened level of miR-125b also increased the expression levels of CCL28 and IGFBP2 in MCF-7 cells (Fig. S6K). This observation supports the use of gene expression of CCL28 and IGFBP2 for our analyses here. We further show in (I) that higher protein level of IGFBP2 (proteome analysis in Kaplan Meier plotter, as measured using reverse phase protein array (RPPA); data not available for CCL28) correlates with significantly shorter OS in docetaxel-treated breast cancer patients. Data not available for DMFS analysis.

(A-G) Log-rank *P* values and hazard ratios with 95% confidence interval, generated by Kaplan Meier plotter, are shown. Patients who had undergone endocrine therapy were excluded from our analysis here. We further observed that stratification survival analyses for breast cancer patients treated with endocrine therapy but not chemotherapy did not reach statistical significance (for OS & DMFS for IGFBP2) or showed a reverse trend (high CCL28 expression correlates with longer DMFS; no data available for OS) (Result not shown).

**B**

**D**

**A**

**E**

**C**

**G**

**F**

**Fig. S4.**

**Fig. S4. Relative survival analysis of miR-125b-associated behavior of breast cancer cell lines and xenografted tumors.** (**A-C**) Number of viable MCF-7 (A), SK-BR3 (B) and MDA-MB-468 (C) breast cancer cells present in “heterogeneous and “homogeneous” groups. Red bars: miR-125b^m^ cells; black/grey bars: Control^m^ cells. (**D**) Schema for determination of RS_α_ and RF_α_ using NSG mice xenograft. (**E**) Number of viable cells, as well as percentage of miR-125b^m^ and Control^m^ cells, in xenograft tumor harvested from mice with or without exposure to sub-lethal dose of docetaxel (1mg/kg). (**F,G**) Bar chart depicting RS_α_ ratio (F) and box plot of RF_α_ for “heterogeneous” group (G) for MCF7. Experiment was performed as depicted in Fig. 1D, except that pcDNA™6.2-GW/EmGFP-miR-125b or pcDNA™6.2-GW/EmGFP empty vector was transfected into the GFP-tagged subpopulation.

Experiment repeated three times, representative data shown (A-C). *n* = 3 independent mice per treatment group (E). Data are mean ± s.d. from two independent biological sets of four technical replicates (F,G). NT: no treatment; DTX: docetaxel treatment.

**L**

**O**

**A**

**K**

**G**

**H**

**E**

**B**

**C**

**D**

**I**

**J**

**M**

**N**

**F**

**Fig. S5.**

**Fig. S5. Mechanism of miR-125b-driven fitness disadvantage of cancer cell altruism.** (**A,C**) Immunoblotting to detect expression of indicated proteins extracted from mimic (A) or LNA inhibitor (C)-transfected MCF7 or MDA-MB-468 cell lines. (**B**) Immunoblotting to detect expression of indicated proteins extracted from mimic- (Left) or pcDNA™6.2-GW/EmGFP plasmid (Right)-transfected MCF7 cells. (**D**) Scatter plots showing correlation between indicated genes. Stronger anti-correlation is observed for the cell cycle genes with miR-125b (first row) or *IKBKB* (gene coding for IKKβ*)* (third row), as compared to miR-125a (second row). Data obtained from GEO dataset GSE58215 that contains microarray-based expression profiles of both microRNAs and mRNAs of 283 breast cancer samples. (**E**) Immunoblotting to detect expression of indicated proteins extracted EGFP-sorted fractions of indicated cell lines. (**F**) Upper: miR-125b binding sites in 3′UTR of mRNA of *E2F3*, *CDK2* and *CCNA2* for both wild-type and mutated (in red) sequences. Underlined sequences are seed binding sequences for miR-125b. (**G,H**) miR-125b^m^ or Control^m^ MCF7 cells were either treated with indicated small molecule drugs targeting components of NF-κB (G) or Wnt/Hedgehog/Notch (H) signaling pathways or not treated. Red circles: some of the small molecule drugs that resulted in notable percentage increase in viability in miR-125b^m^ cells (treatment vs. non-treatment) as compared to Control^m^ (treatment vs. non-treatment) cells, and whose targets were chosen for further investigation. MLN120B and BMS-345541are IKKβ inhibitors; AR-A014418 is GSK-3 inhibitor; iCRT14 inhibits the interaction between β-catenin and TCF4; and cyclopamine is SMO inhibitor. While SMO is not part of the Wnt pathway, it is selected for further study as there is substantial molecular crosstalk between Wnt and Hedgehog signaling pathway. (**I,J**) Homogeneous and heterogeneous mixtures of of MCF7 (I) or MDA-MB-468 (J) were transfected with siRNA against indicated genes, exposed to sublethal 0.8 nM docetaxel, and the RF_α_ measured (Fig. 2E)**,** and percentage rescue in RF_α_ calculated. *IKBKB* gene codes for IKKβ. (**K,L**) Bar chart depicting RS_α_ ratio for MCF-7 xenograft of “heterogeneous” group with or without knockdown of *IKKβ* in the miR-125b^m^ fraction, all with docetaxel exposure (K). Dot: single section read; dots of same colour: sections imaged from same animal. Representative images of xenograft tumor biopsy from “homogeneous” and “heterogeneous” group with or without *IKBKB* knockdown in miR-125b^m^ fraction (L). Black arrows: cells stained positive for Mi/EGFP. (**M**) Immunoblotting to detect for indicated proteins extracted from indicated cell lines transfected with combination of indicated mimics and siRNAs for *p65*. (**N,O**) Bar chart depicting NF-κB reporter activities of pure population of Control^m^ or miR-125b^m^-transfected MCF7 (N) and MDA-MB-468 (O) cells with knockdown of indicated genes. Blue dotted line indicates reporter activity for Control^m^ cells with control siRNA (blue bar); red dotted line for miR-125b^m^ cells with control siRNA (red bar). Percentage reduction in reporter activity (See “methods” for calculation) is also shown (Lower).

Experiments performed twice, representative data shown (A-C, E, M). Biological replicate = 1, technical replicate = 3 (G, H). *n* = 4 independent mice per treatment group (K,L). Data are mean ± s.d. from three independent biological triplicates, each with three technical replicates (I,J,N,O). Statistical analysis was performed using Pearson’s correlation (D), Two-tailed one sample *t*-test against 0 (I,J) or 1 (K), and one-way ANOVA with post-hoc Tukey HSD (N,O). Exact *P* values are shown.

**Fig. S6.**

**A**

**B**

**C**

**E**

**H**

**D**

**J**

**I**

**K**

**F**

**G**

**Fig. S6. Mechanism of miR-125b-driven fitness benefit of cancer cell altruism.** (**A**) Graph showing percentage benefit achieved as a function of percentage of miR-125b^m^ MCF7 altruistic cells. The relationship between percentage benefit and percentage miR-125b^m^ altruists is best described by non-linear regression (quadratic model, R^2^ = 0.8853, *p* < 0.0001). Observation of a downward concave curve here indicates that maximum benefit was attained at low miR-125b^m^ percentage. (**B**) Marginal benefit, which measures the amount of increase in percentage benefit for every per cent increase in MCF7 miR-125b^m^ altruists, was derived from (A) (See “Methods”). The highest rate of increase in percentage benefit (highest marginal benefit) was attained in the 1-10% miR-125b^m^ altruist interval. Marginal benefit markedly dropped when altruist proportion increases further. Category values for x-axis is determined as described in “Materials & Methods”. (**C-E**) MDA-MB-231^miR-125bprom-EGFP^ cells were sorted according to Mi/EGFP levels using maximum and minimum thresholds set at 10%. Single cells were isolated and lysed with total RNA extracted for RNA-seq. (C) Hierarchical and Mi/EGFP^Low^ cells. Significant differentially expressed genes with |log 2 (Fold change)|≥ 2 and adjusted *P* values ≤ 0.05 shown. (D) Principal component (PC) analysis. (E) Gene ontology (GO) enrichment analysis. GO terms with a Benjamini-Hochberg false discovery rate (FDR) corrected *P* ≤ 0.05 were considered significantly enriched by differentially expressed genes. (**F**) Immunoblotting to detect CCL28 and IGFBP2 in conditioned medium from mimic (Left) or pcDNA™6.2-GW/EmGFP-miR-125b plasmid (Right)-transfected MCF7 cells. Ponceau S was used to visualize protein load. Quantification of band intensities (relative to control mimic or plasmid) is shown. (**G**) Plasma levels of CCL28 (Left) and IGFBP2 (Right) during taxane-based chemotherapy treatment, and three months post-treatment. (**H**) Cell viability, as determined using MTS assay, of MDA-MB-231 cells with or without treatment with indicated dosage of docetaxel and/or monoclonal antibodies against IGFBP2, CCL28 or both. NT: no treatment; DTX: 2nm Docetaxel. (**I**) Cell viability, as determined using MTS assay, of MDA-MB-231 cells without (NT) or with 2 nM of docetaxel (DTX), and antibodies against indicated receptors or control IgG. (**J)** Two-color flow cytometry analysis of the cell surface expression of the CCR10 receptor on MDA-MB-231^miR-125bprom-EGFP^ and MDA-MB-415^miR-125bprom-EGFP^ cells were sorted according to Mi/EGFP levels using maximum and minimum thresholds set at 10%. The cells were either unlabelled (Left) or labelled with an Alexa Fluor 647-conjugated anti-CCR10 antibody (Right). Mi/EGFP^High^ and Mi/EGFP^Low^ cells appeared to harbor comparable levels of CCR10 receptor, as observed by their similar levels of Alexa Fluor-647 stain intensity. (**K**) RT qPCR was performed on Control^m^ or miR-125b^m^ MCF-7 cells to detect transcript levels of CCL28 and IGFBP2. Bar charts depict fold change in expression levels of CCL28 and IGFBP2 (relative to Control^m^ cells), with or without exposure to 2nM docetaxel.

Experiment repeated twice, representative data shown; data are mean ± s.d. from 5 technical replicates of one representative biological set (A,B). *n* = 9 Mi/EGFP^Low^ cells and n = 12 Mi/EGFP^High^ cells (C-E). Data are mean ± s.d. from three (H) or two (I) independent biological sets of four technical replicates, or two (K) independent biological sets of three technical replicates. Experiment repeated twice, representative data shown (F,J). *n* = 20 breast cancer patients (G). Statistical analysis was performed using two-tailed Wilcoxon signed-rank test (G), one-way ANOVA with post-hoc Tukey HSD (H) and two-tailed unpaired *t*-test (I,K). Exact *P* values are shown.

3

2

1

0

-1

**A**

**B**

**C**

**D**

**E**

**G**

**H**

**F**

**Fig. S7.**

1

2

3

**Fig. S7. Heightened co-expressions of oncogenes and tumor suppressors in miR-125b^High^ altruists.** (**A-D**) Significant DEGs (FDR<0.05) were determined for miR-125b^High^ vs miR-125b^Low^ fractions of four different breast cancer cell lines. Significant differentially expressed genes (DEGs) (FDR < 0.05) were also determined for ER+ breast cancer tissue vs corresponding normal adjacent tissue, and triple negative breast cancer (TNBC) tissue vs corresponding normal adjacent tissue in the public dataset GSE58135. The DEG status of all genes were classified as “not sig” for no significance in the comparison, “high” for over-expression and “low” for under-expression in tumor versus normal adjacent control or miR-125b^High^ vs miR-125b^Low^. The DEG status of the cell line was then compared to the corresponding tumor comparison using a Chi-square test to identify if there is an association of the DEG states between the two comparisons. Significance was detected for all computed Chi-square tests. Bubble plots of the comparisons are depicted with the DEG states of each of the comparisons in the X and Y axes - this allows one to understand the ratio of observed gene numbers over the expected gene number for each of the 9 possible combinations of DEG states. The size of the bubble is proportional to the log2 transform of the observed gene number over the expected gene number ratio (“abs(log2(observed_over_expected))” ). The bubbles are colored red by the same log2 ratios and are in red when there is an over-representation of the observed and blue when there is an under representation of the observed (“log2(observed_over_expected)”). Over-representation of opposing significant DEG states (high/low and low/high) indicates that the changes due to miR-125b status have opposing tumor effects (as represented by the ER+ and TNBC tissue comparisons against normal adjacent tissues). (A) Comparison of MCF7 miR-125b^m^ vs control^m^ DEG states against ER+ vs adjacent normal DEG states. (B) Comparison of MDA-MB-468 miR-125b^m^ vs control^m^ DEG states against TNBC vs adjacent normal DEG states. (C) Comparison of MDA-MB-231 miR-125b^High^ (Mi/EGFP^High^) vs miR-125b^Low^ (Mi/EGFP^Low^) DEG states against TNBC vs adjacent normal DEG states. (D) Comparison of MDA-MB-415 miR-125b^High^ (Mi/EGFP^High^) vs miR-125b^Low^ (Mi/EGFP^Low^) DEG states against ER+ vs adjacent normal DEG states. (**E-H**) Heat maps of selected DEGs of Mi/EGFP^High^ vs Mi/EGFP^Low^ ((G) MDA-MB-231 and (H) MDA-MB-415) and miR-125^m^ vs control^m^ ((E) MCF7 and (F) MDA-MB-468). The log2 RPKM expression values for each gene is *z*-scaled and depicted in red for higher-than-average expression and blue for lower than average expression. The DEGs were selected for significance (FDR < 0.05), over-expressed in miR-125b^High^ or miR-125b^m^ and annotated as oncogene and/or tumor suppressor gene (oncogene and tumor suppressor gene status obtained from Cancer Gene Census, Network of Cancer Genes, Cancer genome landscape and TCGA oncogenic signaling pathways). The corresponding state of expression (“er_deg_state” for comparison between ER+ breast cancer tissue vs corresponding normal adjacent tissue; “tnbc_deg_state” for comparison between TNBC tissue vs corresponding normal adjacent tissue) and the log2 fold change values (“er_logfc” for ER+/normal adjacent comparison; “tnbc_logfc” for TNBC/normal adjacent comparison) are also shown for each gene indicated. Our data demonstrate over-expression of both oncogenes and tumor suppressors, or over-expression of genes significantly up- and down-regulated in breast tumors (vs. normal adjacent tissues) in miR-125b^High^ fractions, as compared to the miR-125b^Low^ fractions for all four breast cancer cell lines investigated.

**A**

**D**

**E**

**C**

**B**

**G**

**F**

**H**

**I**

**Fig. S8.**

**Fig. S8. MiR-125b^High^ altruists are regenerable via a KLF2-mediated acetylation.** (**A**) Sub-lethal dose (2 nM) docetaxel-treated MDA-MB-231*^miR-125bprom-EGFP^* cells were sorted according to Mi/EGFP levels using maximum and minimum thresholds set at 10%. Untreated parental cells were used as background control (grey). Panel I : Typical histogram of pre-sorted cells. Panel II: Isolated Mi/EGFP^High^ or Mi/EGFP^Low^ cells 2 hours after FACS. Cells were used for xenograft establishment using NSG mice. Panel III: After tumor size reached 150 mm in diameter, tumors were excised from mice and disaggregated using trypsin. Single cells released in the process were subjected to FACS to detect the level of EGFP. *n* = 3 independent animals for each group. Tumor from each animal was cut into half, and section from each half trypsinized and cells released were examined using FACS. Representative histogram from each EGFP fluorescent group is shown here. (**B**) Immunoblotting to detect IGFBP2 and CCL28 in conditioned media from indicated cell lines (Left). Ponceau S was used to visualize protein load. Quantification of band intensities (relative to Mi/EGFP^Low^) is indicated (Right). (**C**) Principal Component Analysis (PCA) plots of gene expression of Mi/EGFP^High^, Mi/EGFP^Mid^ and Mi/EGFP^Low^ fractions on indicated days post-sort for the indicated cell lines. (**D**) Histograms for Day 14 post-sort as described in Fig. 3B. Regenerated MDA-MB-231*^miR-125bprom-EGFP^* cells were treated with 20 nM docetaxel for 12 h, after which Mi/EGFP level was measured using FACS. (**E**) Mi/EGFP fluorescence level of MDA-MB-231^miR-125bprom-EGFP^ cells treated with 20 nM of docetaxel alone, or with 20 nM of histone acetylase inhibitors anacardic acid (AA), a-methylene-g-butyrolactone-3 (MB-3) or curcumin, as determined by FACS. Only AA mitigated docetaxel-induced increase in Mi/EGFP level, indicating involvement of specific histone acetyltransferase (HATs) PCAF and/or TIP60, but not GCN5 (inhibited by MB-3) or p300/CBP (inhibited by curcumin) in the acetylation of the promoter in response to docetaxel exposure. Histogram of non-fluorescent parental line in grey; non-treated cells is shown in dark grey. (**F**) Mi/EGFP fluorescence level of MDA-MB-231^miR-125bprom-EGFP^ cells transfected with indicated siRNA as determined using FACS. These two HATs (TIP60 and PCAF) were chosen due to experimental results as obtained in (E). The transcriptional factors (SP1, KLF2, FOXO3a, TP53 & AhR) were selected based on analysis of the putative promoter of *hsa-miR-125b-*1 gene using Tfsitescan (http://www.ifti.org/Tfsitescan/) and TFBIND (http://tfbind.hgc.jp/) to identify putative binding sites of these transcription factors. We observed that siRNAs against PCAF and KLF2 decreased Mi/EGFP fluorescence, implicating these proteins in the regulation of the promoter. Histogram of non-fluorescent parental line is shown in light grey; cells treated with negative control siRNA in grey, and target siRNA-treated cells in red outline. Percentage ± s.d. cells in gated region for target or control siRNA treatment indicated in red and grey respectively. (**G**) Schema showing putative consensus sites (underlined) for KLF2 binding (CACCC or CGCCC in GC rich region, read in either direction) along the *hsa-miR-125b-1* promoter. (**H**) Unsorted MDA-MB-231^miR-125bprom-EGFP^ cells were transduced with lentivirus for expression of CRISPRi targeting the KLF2 binding site 1 (KBS-1) or binding site 2 (KBS-2) on *hsa-miR-125b-1* promoter or non-specific control sequence. Two sequences were designed and tested for each KLF2 consensus binding site. Histogram of non-fluorescent parental line shown in light grey; control CRISPRi cells in grey, and CRISPRi against KBS-1 or -2 in red outline. Percentage ± s.d. cells in gated region for KBS-1/2 or control CRISPRi indicated in red and grey respectively. (**I**) MDA-MB-231*^miR-125bprom-EGFP^* cells were sorted according to Mi/EGFP levels using maximum and minimum thresholds set at 10% and the fluorescence monitored before and after 12 h treatment with indicated concentration of docetaxel. Untreated parental cells were used as background control (grey).

Data are mean ± s.d. from *n*=3 independent animals (A). Experiments repeated two times, representative data shown (C,D,E,F,H,I). Data are mean ± s.d. from three technical replicates of representative set (D,E,F,H). Data are mean ± s.d. from three independent immunoblots (B). NT: no treatment; DTX: docetaxel treatment.

**F**

**Fig. S9.**

**A**

**B**

**E**

**D**

**C**

**Fig. S9. ChIP reveals protein-chromatin interactions at two different KLF2 binding sites.** (**A**) ChIP-seq data from ENCODE (https://screen.encodeproject.org/) showing enrichment of in H3K27Ac histone mark over the promoter region of *hsa-miR-125b-1* gene in mammary epithelial cells from adult female (accession number EH38E1577033) and four out of seven cell lines from ENCODE. The two KLF2-consensus binding sites identified are found to be located within regions of the promoter known to be enriched in histone acetylation. (**B**) MDA-MB-231^miR-125bprom-EGFP^ cells were sorted according to Mi/EGFP levels using maximum and minimum 10% thresholds. ChIP was performed on the Mi/EGFP^High^ and Mi/EGFP^Low^ cells using indicated antibodies (Left). Quantification of relative band intensities (Right). Acetylation of histone H3 and H4 was observed on the promoter for Mi/EGFP^Low^ cells, but not for Mi/EGFP^High^ cells, indicating that histone acetylation may drive regeneration of miR-125^High^ cells from miR-125^Low^ cells. (**C**) Sorted Mi/EGFP^Low^ cells were transfected with siRNA against PCAF or negative control siRNA. Percentage ± s.d. cells in gated region for target or control siRNA treatment indicated in orange and blue respectively. Knockdown of *PCAF* retarded the regeneration of Mi/EGFP^High^ cells from isolated Mi/EGFP^Low^ cells, implicating PCAF-mediated acetylation in the regeneration process. (**D**) Unsorted MDA-MB-231^miR-125bprom-EGFP^ cells were transfected with siRNA against *KLF2* or control sequence. ChIP was performed using indicated antibodies (Left). Quantification of relative band intensities (Right). Inset: Immunoblot of protein extracts. Reduced H3/H4 acetylation on KBS-1 and PCAF binding on KBS-2 with *KLF2* knockdown were observed, alongside diminished KLF2 binding on both sites. These results indicate binding of PCAF to the promoter of *hsa-miR-125b-1* and acetylation of H3 and H4 at KBS-1 is contingent on binding of KLF2 to the promoter. (**E**) ChIP was performed on unsorted MDA-MB-231^miR-125bprom-EGFP^ with control or KBS-1 CRISPRi expression, using indicated antibodies (Left). Quantification of relative band intensities (Right). Reduced binding of KLF2 with ied binding of dCas9 to KBS-1 was observed, when CRISPRi construct targeting KBS-1, and not the control CRISPRi construct, was transfected into the cells. (**F**) Using ChIP-seq, we further demonstrated specific binding of dCas9 to KBS-1 of the *hsa-miR-125b-1* promoter. MDA-MB-231*^miR-125bprom-EGFP^* cells were transduced with lentivirus leading to expression of CRISPRi targeting KBS- on *hsa-miR-125b-1* promoter or control CRISPRi and subjected to ChIP-Seq analysis. The ChIP-Seq profile shown here demonstrates binding of dCas9 to region of the promoter containing the consensus sequence for KLF2 binding (KBS-1). The postulated direction of transcription is indicated.

Non-fluorescent parental line shown in light grey; cells at Day 0 post-sort in dark grey (C). Data are mean ± s.d. from three (B,D) or four (E) independent biological replicates. Experiments repeated two times, representative data shown (C). Experiments performed once for (F). Statistical analysis was performed using two-tailed unpaired *t*-test (B, D, E). Exact *P* values are shown.

**F**

**I**

**D**

**E**

**A**

**C**

**G**

**K**

**J**

Ly294002

(PI3K Inhibitor)

AKT IV

(AKT Inhibitor)

Log Graph

DMSO

Linear Graph

**B**

**H**

**Fig. S10.**

**Fig. S10. Lateral inhibition maintains a sparse spatial organization of altruists.** (**A**) MDA-MB-231^miR-125bprom-EGFP^ cells were sorted for lowest 10% Mi/EGFP levels and the fluorescence monitored after 14 days. The same procedure was repeated for the regenerated cells for three consecutive regeneration rounds. Both Log (Left) and Linear (Right) plots for Mi/EGFP fluorescence level are presented to show regeneration of the long-tail Mi/EGFP^High^ cancer cells. (**B**) Mi/EGFP^High^ and Mi/EGFP^Low^ cell from indicated cancer cell lines were exposed to indicated concentrations of the PI3K inhibitor Ly294002 or AKT inhibitor AKT IV for 12 h, and viability measured. (**C**) Unsorted MDA-MB-231^miR-125bprom-EGFP^ cells were either exposed to DMSO, Ly294002 or AKT IV for three days, after which their fluorescence levels were analyzed by FACS. (**D**) Mi/EGFP^Low^ cell fractions from MDA-MB-231^miR-125bprom-EGFP^ cancer cells were exposed to full conditioned media harvested from a separate batch of Mi/EGFP^High^ and Mi/EGFP^Low^ cells treated with either control or miR-125b LNA inhibitor. After four days, their extracted protein was analyzed by immunoblotting against indicated proteins (Left). Right: schema of workflow. (**E,F**) Mi/EGFP^Low^ cell fractions from indicated cancer cell lines were exposed to Mi/EGFP^Low^ or Mi/EGFP^High^ conditioned media with or without antibodies against IGFBP2 (E), or CCL28 or its receptor CCR10 (F). After four days, their fluorescence levels were analyzed by FACS. (**G**) Mi/EGFP^Low^ cell fractions from MDA-MB-231^miR-125bprom-EGFP^ cancer cells were exposed to conditioned media harvested from a separate batch of Mi/EGFP^High^ cells, recombinant IGFBP2 or recombinant CCL28, in combination with Ly294002 or AKT IV for three days, after which their fluorescence levels were analyzed by FACS. (**H**) Mi/EGFP^Low^ cell fractions from indicated cancer cell lines were exposed to indicated concentrations of docetaxel alone or in combination with Ly294002 or AKT IV for 12 hours, and viability measured. (**I,J**) Mi/EGFP^Low^ cell fractions from indicated cancer cell lines, transfected with control or *GAB1* siRNA, were exposed to Mi/EGFP^Low^ or Mi/EGFP^High^ conditioned medium. After four days, their fluorescence levels were analyzed by FACS (I) and their extracted protein by immunoblotting (J, Left; schema of workflow on Right). (**K**) miR-125b binding sites in 3′UTR of *GAB1* mRNA for both wild-type and mutated (in red) sequences. Underlined sequence is the seed binding sequence for miR-125b.

Experiment performed once (A). Experiments repeated two times, representative data are shown (C,D,J). Experiments repeated two times, mean percentage ± s.d. cells for technical triplicates of representative set are shown in same color as corresponding histograms (E,F,G,I). Data are mean ± s.d. from two independent biological triplicates, each with three technical replicates (B,H). Statistical analysis was performed using two-tailed unpaired *t*-test (B,H). Exact *P* values are shown.

| Fig. No. | Gender | Age at Diagnosis | Histologic type | Grade | Estrogen Receptor | HER2 Receptor | Progesterone Receptor | Taxane-based neoadjuvant, adjuvant or metastatic Chemotherapy? |
| --- | --- | --- | --- | --- | --- | --- | --- | --- |
| S1A | Female | 42 | Ductal Carcinoma | 3 | + | + | - | Neoadjuvant & adjuvant |
| S1B | Female | 44 | Invasive Ductal Carcinoma | 3 | - | - | - | Metastatic |
| S1C | Female | 39 | Ductal and Lobular Carcinoma | 3 | + | - | + | Adjuvant |
| S1D | Female | 39 | Ductal Carcinoma | 3 | - | - | - | Adjuvant |
| S1E | Female | 55 | Infiltrative Ductal Carcinoma | 3 | + | + | + | Adjuvant |
| S1F | Female | 50 | Ductal Carcinoma | 1 | + | - | + | Adjuvant |
| S1G | Female | 50 | Invasive Ductal Carcinoma | Not available | + | - | + | Metastatic |
| S1H | Female | 57 | Ductal Carcinoma | 3 | + | + | + | Neoadjuvant |
| S1I | Female | 37 | Ductal, NOS | 3 | + | + | + | Adjuvant |
| S1J | Female | 36 | Invasive Ductal Carcinoma | 3 | - | - | - | Neoadjuvant |

**Table S1.** Clinical characteristics of breast cancer patients for miRNA profiling of CTCs (Supplementary Fig. 1)

| No. | Gender | Age at Diagnosis | Histologic type | Grade | Estrogen Receptor | HER2 Receptor | Progesterone Receptor | Neoadjuvant Chemotherapy? |
| --- | --- | --- | --- | --- | --- | --- | --- | --- |
| 1 | Female | 48 | Invasive Lobular Carcinoma | Not available | - | - | - | Yes, Taxane |
| 2 | Female | 46 | Infiltrating Ductal Carcinoma | 1 | + | + | + | Yes, Taxane |
| 3 | Female | 51 | Infiltrating ductal carcinoma | 2 | + | - | - | Yes, Taxane |
| 4 | Female | 50 | Invasive Ductal Carcinoma | 3 | **-** | + | + | Yes, Taxane |
| 5 | Female | 48 | Invasive Ductal Carcinoma | 3 | + | - | - | Yes, Taxane |
| 6 | Female | 54 | Infiltrating Ductal Carcinoma | 3 | - | + | - | Yes, Taxane |
| 7 | Female | 75 | Infiltrating Ductal Carcinoma | 2 | - | + | + | Yes, Taxane |
| 8 | Female | 67 | Infiltrating Ductal Carcinoma | Not available | - | + | - | Yes, Taxane |
| 9 | Female | 47 | Infiltrating Ductal Carcinoma | 3 | - | - | - | Yes, Taxane |
| 10 | Female | 48 | Invasive Lobular Carcinoma | 2 | + | - | - | Yes, Taxane |
| 11 | Female | 32 | Infiltrating Ductal Carcinoma | 3 | - | + | - | Yes, Taxane |
| 12 | Female | 62 | Infiltrating Ductal Carcinoma | 3 | - | + | - | Yes, Taxane |
| 13 | Female | 59 | Ductal, NOS | 1 | + | - | + | Yes, Taxane |
| 14 | Female | 29 | Infiltrating Lobular Carcinoma | Not available | + | - | + | Yes, Taxane |
| 15 | Female | 34 | Infiltrating Ductal Carcinoma, NOS | 3 | + | + | + | Yes, Taxane |
| 16 | Female | 55 | Invasive Ductal Carcinoma, NOS | 2 | + | - | + | Yes, Taxane |
| 17 | Female | 38 | Invasive Ductal Carcinoma, NOS | 1 | + | - | + | Yes, Taxane |
| 18 | Female | 47 | Ductal Carcinoma *in-situ* | 3 | - | + | - | Yes, Taxane |
| 19 | Female | 32 | Invasive Ductal Carcinoma | 3 | - | + | - | Yes, Taxane |
| 20 | Female | 51 | Invasive Ductal Carcinoma | 3 | - | + | - | Yes, Taxane |
| 21 | Female | 36 | Invasive Ductal Carcinoma | 2 | - | + | + | Yes, Taxane |
| 22 | Female | 67 | Ductal Carcinoma *in-situ* | 2 | + | - | + | Yes, Taxane |
| 23 | Female | 45 | Invasive & Ductal Carcinoma *in-situ* | 2 | + | + | + | Yes, Taxane |
| 24 | Female | 55 | Invasive Ductal Carcinoma | 3 | + | Not available | - | Yes, Taxane |
| 25 | Female | 45 | Invasive Ductal Carcinoma | 2 | + | - | + | Yes, Taxane |
| 26 | Female | 51 | Infiltrative Ductal Carcinoma | 2 | + | Not available | + | Yes, Taxane |
| 27 | Female | 45 | Infiltrating Mucinous Carcinoma | Not available | + | Not available | + | No |
| 28 | Female | 75 | Infiltrating Ductal Carcinoma, NOS | 3 | + | Not available | + | No |
| 29 | Female | 64 | Ductal Carcinoma, NOS | 2 | + | Not available | + | No |
| 30 | Female | 37 | Invasive Ductal Carcinoma with DCIS | 3 | - | Not available | - | No |
| 31 | Female | 51 | Invasive Ductal Carcinoma, NOS | 3 | - | Not available | - | No |
| 32 | Female | 64 | Invasive Carcinoma | 2 | - | Not available | + | No |
| 33 | Female | 50 | Ductal Carcinoma | 2 | + | Not available | + | No |
| 34 | Female | 66 | Ductal Carcinoma | 2 | + | Not available | + | No |
| 35 | Female | 45 | Invasive Ductal Adenocarcinoma, NOS | 3 | - | Not available | - | No |
| 36 | Female | 43 | Invasive and *in-situ* Ductal Carcinoma | 3 | - | Not available | + | No |
| 37 | Female | 52 | Invasive Ductal Adenocarcinoma, NOS | 3 | + | Not available | - | No |
| 38 | Female | 69 | Invasive Ductal Carcinoma, NOS | 2 | + | Not available | - | No |
| 39 | Female | 43 | Invasive Ductal Carcinoma, NOS | 2 | - | Not available | + | No |
| 40 | Female | 44 | Infiltrating Ductal Carcinoma | 2 | - | - | - | No |
| 41 | Female | 62 | Invasive Ductal Carcinoma | 2 | + | Not available | - | No |
| 42 | Female | 51 | Invasive Carcinoma | 2 | - | Not available | + | No |
| 43 | Female | 66 | Infiltrative Ductal Carcinoma | 3 | + | Not available | - | No |
| 44 | Female | 45 | Mucinous Carcinoma | 2 | + | Not available | + | No |
| 45 | Female | 58 | Invasive Ductal Adenocarcinoma, NOS | 2 | - | Not available | - | No |
| 46 | Female | 51 | Infiltrating Ductal Carcinoma | 2 | + | Not available | - | No |
| 47 | Female | 66 | Invasive Ductal carcinoma with DCIS | 1 | + | Not available | + | No |
| 48 | Female | 70 | Invasive Ductal Carcinoma, NOS | 3 | - | Not available | - | No |
| 49 | Female | 26 | Infiltrating Ductal Carcinoma | 3 | - | Not available | - | No |
| 50 | Female | 52 | Infiltrating Ductal Carcinoma | 2 | - | Not available | + | No |
| 51 | Female | 59 | Invasive Ductal Carcinoma, NOS | 1 | + | Not available | + | No |
| 52 | Female | 45 | Invasive Lobular Carcinoma | 2 | - | Not available | + | No |
| 53 | Female | 45 | Invasive Ductal Carcinoma | 3 | - | Not available | + | No |

**Table S2.** Clinical characteristics of breast cancer patients for miR-125b *in-situ­* hybridization (Fig. 1A)

| Gene | Log₂ fold change | Fold change | P-value | FDR p-value | Bonferroni | Docetaxel Treatment? |
| --- | --- | --- | --- | --- | --- | --- |
| *CCNA1* | 0 | 0 | - | - | - | No |
| ***CCNA2*** | **1.23** | **2.35** | **2.86E-06** | **3.76E-05** | **4.72E-02** | **No** |
| *CCNB1* | -0.89 | -1.85 | 4.38E-05 | 4.53E-04 | 7.22E-01 | No |
| *CCNB2* | -0.29 | -1.22 | 5.34E-01 | 7.18E-01 | 1.00E+00 | No |
| *CCNB3* | -0.12 | -1.08 | 9.48E-01 | 9.71E-01 | 1.00E+00 | No |
| *CCND1* | 0.58 | 1.49 | 4.49E-06 | 5.68E-05 | 7.40E-02 | No |
| *CCND2* | -2.78 | -6.88 | 7.24E-02 | 2.29E-01 | 1.00E+00 | No |
| *CCND3* | 1.44 | 2.72 | 3.65E-06 | 4.68E-05 | 6.01E-02 | No |
| *CCNE1* | -0.57 | -1.48 | 4.78E-01 | 6.82E-01 | 1.00E+00 | No |
| *CCNE2* | -0.57 | -1.48 | 1.27E-01 | 3.27E-01 | 1.00E+00 | No |
| ***GAB1*** | **1.29** | **2.45** | **2.32E-08** | **4.48E-07** | **3.82E-04** | **No** |
| *GAB2* | 0.72 | 1.64 | 2.32E-02 | 1.00E-01 | 1.00E+00 | No |
| *GAB3* | 0 | 0 | - | - | - | Yes |
| *CCNA1* | 0 | 0 | - | - | - | Yes |
| ***CCNA2*** | **0.60** | **1.52** | **2.58E-02** | **1.19E-01** | **1.00E+00** | **Yes** |
| *CCNB1* | -0.71 | -1.63 | 9.34E-04 | 8.66E-03 | 1.00E+00 | Yes |
| *CCNB2* | -0.34 | -1.27 | 3.61E-01 | 6.00E-01 | 1.00E+00 | Yes |
| *CCNB3* | 0 | 0 | - | - | - | Yes |
| *CCND1* | 0.47 | 1.38 | 4.91E-03 | 3.33E-02 | 1.00E+00 | Yes |
| *CCND2* | -0.60 | -1.52 | 6.67E-01 | 8.01E-01 | 1.00E+00 | Yes |
| *CCND3* | 0.88 | 1.84 | 2.43E-04 | 2.80E-03 | 1.00E+00 | Yes |
| *CCNE1* | -0.16 | -1.12 | 8.04E-01 | 8.91E-01 | 1.00E+00 | Yes |
| *CCNE2* | -0.18 | -1.13 | 5.89E-01 | 7.57E-01 | 1.00E+00 | Yes |
| ***GAB1*** | **1.08** | **2.12** | **3.31E-06** | **6.46E-05** | **5.52E-02** | **Yes** |
| *GAB2* | 0.91 | 1.88 | 9.40E-03 | 5.54E-02 | 1.00E+00 | Yes |
| *GAB3* | 0 | 0 | - | - | - | Yes |

Note: Genes in **bold** were studied with regards to their involvement in mechanism of altruism in breast cancer cells

**Table S3.** Selected results of comparative analysis of RNA-Sequencing of biotinylated miR-125b mimics versus control mimic pulldown (Fig. 2 & 4)

|  |  | Biological Set 1 | | Biological Set 2 | | Biological Set 3 | |  |
| --- | --- | --- | --- | --- | --- | --- | --- | --- |
| Protein | Gene | Set 1 | Set 2 | Set 1 | Set 2 | Set 1 | Set 2 | Average |
| Hemoglobin subunit beta | HBB | 1.4322 | 4.8306 | 4.0926 | 1.6444 | 1.2134 | 1.3932 | 2.4344 |
| Lactotransferrin | LTF | 1.5996 | 1.9055 | 1.3428 | 1.4454 | 2.8576 | 0.9036 | 1.6757 |
| Meteorin-like protein | METRNL | 1.4723 | 1.3677 | 1.8030 | 1.9409 | 1.2134 | 1.6144 | 1.5686 |
| **Insulin-like growth factor-binding protein 2** | **IGFBP2** | **1.7219** | **2.0324** | **1.3183** | **1.3183** | **1.1482** | **1.5560** | **1.5158** |
| Titin | TTN | 1.7865 | 1.3932 | 1.4588 | 1.3428 | 1.1803 | 1.7219 | 1.4806 |
| Alpha-fetoprotein | AFP | 1.5417 | 1.7061 | 1.2589 | 1.3932 | 1.3804 | 1.5417 | 1.4703 |
| 45 kDa calcium-binding protein | SDF4 | 1.7061 | 1.7865 | 1.5417 | 1.1588 | 1.1272 | 1.2134 | 1.4223 |
| Hemoglobin subunit alpha | HBA1 | 1.4322 | 1.7061 | 1.1695 | 1.1588 | 1.6293 | 0.9908 | 1.3478 |
| Cathepsin D | CTSD | 1.2706 | 1.4859 | 1.4723 | 1.0093 | 1.1272 | 1.6749 | 1.3400 |
| Alpha-enolase | ENO1 | 0.9817 | 0.9036 | 1.8535 | 1.7219 | 0.9120 | 0.8395 | 1.2020 |
| 14-3-3 protein zeta/delta | YWHAZ | 1.2023 | 1.1272 | 1.4859 | 1.0666 | 1.2134 | 0.9817 | 1.1795 |
| Sulfhydryl oxidase 1 | QSOX1 | 0.6668 | 1.0375 | 1.5136 | 0.9638 | 1.5996 | 0.9908 | 1.1287 |
| Triosephosphate isomerase | TPI1 | 1.0000 | 1.1912 | 1.0765 | 0.9550 | 1.2823 | 1.1376 | 1.1071 |
| Inter-alpha-trypsin inhibitor heavy chain H2 | ITIH2 | 1.2023 | 1.0666 | 1.0568 | 0.9462 | 1.0666 | 0.9550 | 1.0489 |
| Keratin, type I cytoskeletal 19 | KRT19 | 1.1066 | 1.0471 | 1.0765 | 1.0280 | 1.0280 | 0.9727 | 1.0432 |
| Metalloproteinase inhibitor 2 | TIMP2 | 1.0568 | 0.9550 | 1.0864 | 0.9817 | 1.1066 | 1.0000 | 1.0311 |
| Heat shock protein HSP 90-alpha | HSP90AA1 | 1.0093 | 0.9727 | 1.0965 | 1.0568 | 1.0568 | 0.9817 | 1.0290 |
| Tubulin beta-4B chain | TUBB4B | 1.1066 | 0.9462 | 1.2134 | 1.0471 | 0.9550 | 0.8166 | 1.0142 |
| Semaphorin-3C | SEMA3C | 1.0000 | 0.9638 | 1.0864 | 1.0471 | 0.9550 | 0.9204 | 0.9955 |
| Alpha-2-macroglobulin | ASML1 | 0.9908 | 0.9290 | 1.0471 | 0.9908 | 1.0186 | 0.9550 | 0.9886 |
| Tubulin alpha-1C chain | TUBA1C | 1.0093 | 0.9908 | 0.9550 | 0.9376 | 0.9908 | 0.9638 | 0.9746 |
| 14-3-3 protein beta/alpha | YWHAB | 1.0280 | 0.9204 | 0.9727 | 0.8790 | 0.9204 | 0.8318 | 0.9254 |
| Pyruvate kinase | PKM | 0.9290 | 0.8872 | 0.9376 | 0.8954 | 0.9550 | 0.9120 | 0.9193 |
| Keratin, type I cytoskeletal 18 | KRT18 | 1.0000 | 0.8710 | 0.9908 | 0.8630 | 0.9290 | 0.8091 | 0.9105 |
| Fibronectin | FN1 | 0.8790 | 0.8241 | 0.9290 | 0.8710 | 0.9727 | 0.9204 | 0.8994 |
| Putative heat shock protein HSP 90-beta 2 | HSP90AB2P | 0.8954 | 0.7870 | 0.8630 | 0.7586 | 0.8630 | 0.7586 | 0.8209 |
| Heat shock 70 kDa protein 1A/1B | HSPA1A | 0.5808 | 0.5754 | 0.9290 | 0.8630 | 0.7798 | 0.7447 | 0.7455 |
| Cofilin-1 | CFL1 | 0.5546 | 0.6368 | 0.5346 | 0.5808 | 0.6368 | 0.6668 | 0.6017 |
| Neuroblast differentiation-associated protein | AHNAK | 0.7656 | 0.4169 | 0.7516 | 0.4130 | 0.7943 | 0.4325 | 0.5957 |
| Cystatin-C | CST3 | 0.4130 | 0.7727 | 0.4875 | 0.9036 | 0.2208 | 0.7656 | 0.5939 |
| Fructose-bisphosphate aldolase A | ALDOA | 0.6607 | 0.4018 | 0.7047 | 0.4487 | 0.7516 | 0.4406 | 0.5680 |
| Dynein heavy chain 11, axonemal | DNAH11 | 0.6486 | 0.3342 | 0.2148 | 0.5808 | 1.0093 | 0.3342 | 0.5203 |
| Heat shock protein beta-1 | HSPB1 | 0.3698 | 0.3105 | 0.4169 | 0.5012 | 0.4446 | 0.4920 | 0.4225 |

Note: Protein in **bold** was identified for further investigation in this study.

**Table S4.** Results of comparative proteomic analysis of conditioned media from miR-125b or control mimic-transfected MCF7 cells using iTRAQ (Fig. 2H)

| No. | Gender | Age at Diagnosis | Histologic Type | Grade | Estrogen Receptor | HER2 Receptor | Progesterone Receptor | Taxane-based neoadjuvant, adjuvant or metastatic Chemotherapy? |
| --- | --- | --- | --- | --- | --- | --- | --- | --- |
| 1 | F | 46 | Ductal, not otherwise specified (NOS) | 2 | + | - | + | Adjuvant |
| 2 | F | 40 | Invasive carcinoma with lobular features | Not available | + | - | + | Neoadjuvant |
| 3 | F | 46 | Ductal, not otherwise specified (NOS) | 3 | + | + | + | Adjuvant |
| 4 | F | 38 | Ductal, not otherwise specified (NOS) | 3 | + | - | + | Adjuvant |
| 5 | F | 47 | Benign Breast Tissue with Fibrocystic change and Intraductal Papilloma | 2 | + | - | + | Adjuvant |
| 6 | F | 42 | Ductal Carcinoma | 3 | + | + | - | Neoadjuvant |
| 7 | F | 39 | Ductal and Lobular Carcinoma | 3 | + | - | + | Adjuvant |
| 8 | F | 39 | Ductal Carcinoma | 3 | - | - | - | Adjuvant |
| 9 | F | 50 | Ductal Carcinoma | 1 | + | - | + | Adjuvant |
| 10 | F | 50 | Invasive Ductal Carcinoma | 2 | - | + | - | Adjuvant |
| 11 | F | 58 | Invasive Ductal Carcinoma | 3 | + | - | + | Adjuvant |
| 12 | F | 50 | Invasive Ductal Carcinoma | 3 | - | + | - | Adjuvant |
| 13 | F | 57 | Invasive Ductal Carcinoma | 3 | + | + | + | Neoadjuvant |
| 14 | F | 41 | Invasive Ductal Carcinoma | 2 | + | + | + | Adjuvant |
| 15 | F | 50 | Invasive Ductal Carcinoma | 3 | + | + | - | Adjuvant |
| 16 | F | 45 | Invasive Ductal Carcinoma/  Invasive Lobular Carcinoma | 1 | + | - | + | Adjuvant |
| 17 | F | 49 | Infiltrative Ductal Carcinoma | 2 | + | - | + | Adjuvant |
| 18 | F | 46 | Invasive Ductal Carcinoma | 2 | + | + | + | Adjuvant |
| 19 | F | 40 | Invasive Ductal Carcinoma | 2 | + | - | + | Adjuvant |
| 20 | F | 39 | Invasive Ductal Carcinoma | 3 | + | - | + | Metastatic |

**Table S5.** Clinical characteristics of breast cancer patients for detection of IGFBP2 and CCL28 protein levels in plasma (Supplementary Fig. 6G)

References:

67. West SA, Griffin AS, Gardner A, Diggle SP. Social evolution theory for microorganisms. Nature Reviews Microbiology. 2006; 4:597–607.

68. Leggett HC, Brown SP, Reece SE. War and peace: social interactions in infections. Philos Trans R Soc Lond B Biol Sci.2014; 369:20130365–20130365.

69. McNally L, Brown SP. Building the microbiome in health and disease: niche construction and social conflict in bacteria. Philos Trans R Soc Lond B Biol Sci. 2015; 370:20140298.

70. Gardner A, Hardy ICW, Taylor PD, West SA. Spiteful soldiers and sex ratio conflict in polyembryonic parasitoid wasps. Am Nat. 2007; 169:519–33.

71. Wloch-Salamon DM. Sociobiology of the budding yeast. J Biosci. 2014;39:225–36.

72. Okasha, Samir. Biological altruism. The Stanford encyclopedia of philosophy. Metaphysics Research Lab, Stanford University; 2013. https://plato.stanford.edu/archives/fall2013/entries/altruism-biological/

73. Archetti M. Cooperation as a volunteer’s dilemma and the strategy of conflict in public goods games. J Evol Biol. 2009; 22:2192–200.

74. Doebeli M. The evolutionary origin of cooperators and defectors. Science. 2004; 306:859–62.

75. Wynne-Edwards VC. Animal dispersion in relation to social behaviour. Edinburgh: Oliver and Boyd; 1962.

76. Archetti M, Scheuring I. Review: Game theory of public goods in one-shot social dilemmas without assortment. J Theor Biol. 2012; 299:9–20.

77. Archetti M. How to analyze models of nonlinear public goods. Games. 2018; 9:17.

78. Perc M, Gómez-Gardeñes J, Szolnoki A, Floría LM, Moreno Y. Evolutionary dynamics of group interactions on structured populations: a review. J R Soc Interface. 2013; 10:20120997.

79. Archetti M. Cooperation among cancer cells as public goods games on Voronoi networks. J Theor Biol. 2016;396:191–203.

80. George JT, Levine H. Optimal cancer evasion in a dynamic immune microenvironment generates diverse post-escape tumor antigenicity profiles. eLife. 2023; 12:e82786.
